# Reversible phase separation of ESCRT protein ALIX through tyrosine phosphorylation

**DOI:** 10.1126/sciadv.adg3913

**Published:** 2023-07-14

**Authors:** Ruben D. Elias, Yingqi Zhu, Qi Su, Rodolfo Ghirlando, Jin Zhang, Lalit Deshmukh

**Affiliations:** ^1^ Department of Chemistry and Biochemistry, University of California, San Diego, La Jolla, CA 92093, USA.; ^2^ Department of Pharmacology, University of California, San Diego, La Jolla, CA 92093, USA.; ^3^ Laboratory of Molecular Biology, National Institute of Diabetes and Digestive and Kidney Diseases, National Institutes of Health, Bethesda, MD 20892, USA.; ^4^ Department of Bioengineering, University of California, San Diego, La Jolla, CA 92093, USA.

## Abstract

Cytokinetic abscission, the last step of cell division, is regulated by the ESCRT machinery. In response to mitotic errors, ESCRT proteins, namely, ALIX, CHMP4B, and CHMP4C, accumulate in the cytosolic compartments termed “abscission checkpoint bodies” (ACBs) to delay abscission and prevent tumorigenesis. ALIX contributes to the biogenesis and stability of ACBs via an unknown mechanism. We show that ALIX phase separates into nondynamic condensates in vitro and in vivo, mediated by the amyloidogenic portion of its proline-rich domain. ALIX condensates confined CHMP4 paralogs in vitro. These condensates dissolved and reformed upon reversible tyrosine phosphorylation of ALIX, mediated by Src kinase and PTP1B, and sequestration of CHMP4C altered their Src-mediated dissolution. NMR analysis revealed how ALIX triggers the activation of CHMP4 proteins, which is required for successful abscission. These results implicate ALIX’s phase separation in the modulation of ACBs. This study also highlights how posttranslational modifications can control protein phase separation.

## Teaser

Tyrosine phosphorylation-mediated phase separation of ESCRT-factor ALIX likely modulates the timing of cell division.

## INTRODUCTION

**Fig. 1.**
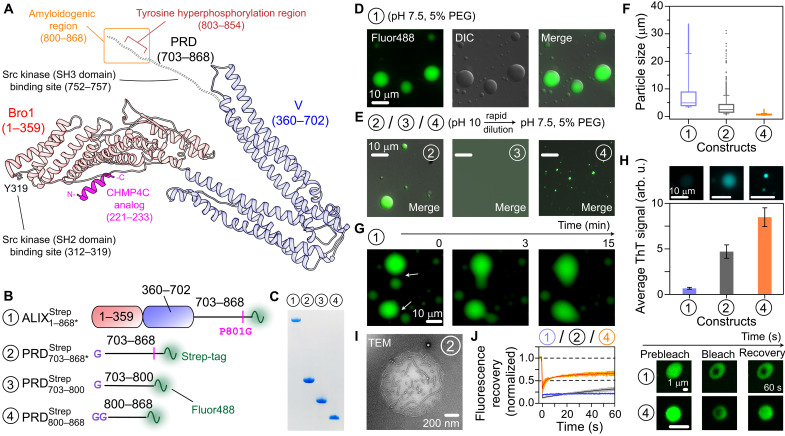
Phase separation of recombinant ALIX. (**A**) Schematic of ALIX comprising Bro1, V, and PRD (red and blue ribbons and dashed black lines, respectively), derived from the x-ray structure of Bro1-V domains (*71*); numbers in parentheses signify ALIX residues. The functional motifs of ALIX relevant to current study are marked and labeled (*19*, *22*, *23*). The model also depicts a CHMP4C analog (magenta ribbon) in its Bro1-bound form, obtained from the x-ray structure of Bro1-CHMP4C peptide complex (*10*). (**B**) ALIX constructs (fig. S6) tested in phase separation experiments. Each construct is designated by a circled number. The positions of the strep tags, the P801G mutations, and Alexa Fluor 488 labeling sites are marked. Remnant residues of the TEV protease cleavage sites are labeled in purple. (**C**) SDS–polyacrylamide gel electrophoresis analysis of ALIX constructs; throughout the figure, the circled numbers signify the constructs shown in (B). (**D** and **E**) Microscopy images of droplets made by ALIX constructs ; ${\mathrm{PRD}}_{703-800}^{\mathrm{Strep}}$ (construct 3) did not phase separate. (**F**) Box plot of the size distribution of condensates of ${\mathrm{ALIX}}_{1-868*}^{\mathrm{Strep}}$ (blue), ${\mathrm{PRD}}_{703-868*}^{\mathrm{Strep}}$ (gray), and ${\mathrm{PRD}}_{800-868}^{\mathrm{Strep}}$ (orange); *n* ≥ 1900. (**G**) Representative montage of the slow fusion of ${\mathrm{ALIX}}_{1-868*}^{\mathrm{Strep}}$ condensates. (**H**) Quantitative analysis of the co-partitioning of 20 μM ThT dye in freshly prepared condensates (*n* ≥ 25) of ALIX constructs [same color scheme as (F)]. Top: Representative images of the corresponding condensates. (**I**) Representative TEM image of an aged ${\mathrm{PRD}}_{703-868*}^{\mathrm{Strep}}$ droplet; incubation time ~2 days at room temperature. (**J**) FRAP analysis, *n* = 3; mean (solid line) and SD (shaded region). Right: Fluorescence of representative condensates of ${\mathrm{ALIX}}_{1-868*}^{\mathrm{Strep}}$ and ${\mathrm{PRD}}_{800-868}^{\mathrm{Strep}}$ at different time points. All phase separation experiments were performed with 50 μM proteins. The concentration of streptavidin Alexa Fluor 488 was 0.2 mg/ml. arb. u, arbitrary units.

Cytokinetic abscission, the final step of cell division, is tightly regulated and coordinated with chromosome segregation to ensure accurate distribution of genetic material (*1*). In animal cells, it is carried out by the endosomal sorting complex required for transport (ESCRT) machinery, which severs a membranous intercellular bridge, comprising a microtubule-rich structure called the midbody (*2*). Five functionally distinct ESCRT factors/complexes, namely, apoptosis-linked gene 2–interacting protein X (ALIX), ESCRT-I, ESCRT-II, ESCRT-III, and adenosine triphosphatase (ATPase) vacuolar protein sorting–associated protein 4 (VPS4), are sequentially recruited to the midbody with the localization of charged multivesicular body protein 4B (CHMP4B), a late-acting ESCRT-III paralog that polymerizes into membrane-constricting filaments, initiating the final separation of the daughter cells (fig. S1). The early-acting ESCRT components, tumor susceptibility gene 101 (TSG101; ESCRT-I) and ALIX, are initially recruited to the midbody by the microtubule-bundling centrosomal protein of 55 kDa (CEP55). ALIX (Fig. 1A), the central regulator of abscission, performs multiple functions. ALIX binds to TSG101, thereby initiating TSG101-mediated recruitment of CHMP4B to the midbody. ALIX also facilitates an alternative CHMP4B recruitment pathway as its Bro1 domain binds to the extreme C-terminal motifs of CHMP4 paralogs (*3*). Moreover, ALIX promotes closed-to-open transitions of inactive cytosolic CHMP4 monomers by a yet unknown mechanism, triggering their polymerization required for abscission (*4*). In response to chromosome segregation defects and mitotic errors, a conserved Aurora-B kinase–dependent mechanism known as the “abscission checkpoint” (NoCut pathway in yeast) arrests the abscission to prevent the accumulation of DNA damage (*5*–*7*). Unlike CHMP4B, which is indispensable for cytokinetic membrane fission, its paralog, CHMP4C, is instrumental in maintaining the checkpoint (*8*). The latter depends on its interactions with ALIX, as an allele of CHMP4C (with A232T point mutation) defective in binding to ALIX overrides the checkpoint, resulting in increased susceptibility to several cancers, including ovarian cancer (*9*, *10*).

The abscission timing depends on the differential spatiotemporal distribution of ALIX and CHMP4 paralogs. In these regard, cytoplasmic compartments termed “abscission checkpoint bodies” (ACBs), which form during stress conditions that activate the checkpoint, were recently discovered (*11*). These ACBs stem from cytoplasmic structures known as mitotic interchromatin granules (MIGs), which, in turn, originate from nuclear speckles, the nuclear compartments associated with active transcription sites (*12*). Phase separation of biomolecules into membraneless compartments serves numerous functions, including sequestration and storage of cellular factors (*13*–*16*). Both MIGs and nuclear speckles exhibit liquid-liquid phase separation characteristics (*17*, *18*). A prolonged abscission checkpoint induces the transition of MIGs into ACBs. The latter confine multiple abscission factors, including ALIX, CHMP4B, CHMP4C, and Aurora-B, thus delaying the localization of ALIX and, consequently, the ALIX-mediated localization of CHMP4B at the midbody (*11*). ALIX maintains the integrity of ACBs, as depletion of ALIX was shown to reduce their size substantially (*11*). In addition, the recruitment of ALIX contributes to the biogenesis of ACBs from MIGs. However, the mechanism(s) by which ALIX maintains the integrity of ACBs and orchestrates their transformation from MIGs is unclear. Moreover, although Src kinase–mediated phosphorylation of ALIX regulates its cellular functions (*19*), Src signaling is required for successful abscission (*20*), and protein tyrosine phosphatase 1B (PTP1B) targets the ESCRT machinery (*21*), the interplay between reversible tyrosine phosphorylation of ALIX and the biogenesis and stability of ACBs is not known. A mechanistic understanding of these processes will provide crucial insights into how order is achieved in the last step of cell division as well as the impact and role of posttranslational modifications (PTMs) in regulating the timing of protein compartmentalization.

Here, we describe our discovery of ALIX’s phase separation in vivo and in vitro. We show that condensates of recombinant ALIX readily confine CHMP4 paralogs, CHMP4B and CHMP4C. Nuclear magnetic resonance (NMR) measurements provided mechanistic insights into how ALIX triggers CHMP4 activation needed for membrane scission. The formation and dissolution of condensates of recombinant ALIX could be tuned by PTP1B and Src. Thus, we propose that phase separation of ALIX will play a vital role in the biogenesis of ACBs and in maintaining their integrity, and that, upon resolution of the checkpoint, Src-mediated dissolution of ALIX assemblies will reroute ALIX from ACBs to the midbody, thereby controlling the abscission timing.

## RESULTS AND DISCUSSION

### ALIX condensates and identification of its phase separation motif

We sought to determine the phase separation characteristics of recombinant ALIX because the extreme C-terminal portion of its disordered proline-rich domain (PRD; Fig. 1A) formed β sheet–rich amyloid fibrils (*22*, *23*), and amyloidogenic sequences may phase separate (*13*, *14*). Heterologous expression of ALIX in *Escherichia coli* is hampered by its PRD, which induces ribosomal stalling (*22*). We resolved these expression issues by introducing a point mutation, P801G, in its PRD, which enabled a high-yield expression of recombinant ALIX (~40 mg/1 liter of bacterial culture). This P801G substitution likely works by altering the ^800^GPP^802^ motif of ALIX, which is known to induce polyproline-mediated ribosomal stalling in bacteria (*24*). Note that we previously used this substitution to produce milligram quantities of recombinant ALIX-PRD (*23*). The P801G substitution resides in the CEP55-binding motif of ALIX-PRD, residues 797 to 808 (*25*). Mutated ALIX-PRD, however, retained its CEP55-binding ability (see below), likely because structurally dynamic PRDs can often tolerate combinations of various residues without compromising their functional integrity (*22*). The following constructs were used to test phase separation of ALIX (Fig. 1, B and C): full-length ALIX (${\mathrm{ALIX}}_{1-868*}^{\mathrm{Strep}}$), a construct representing its PRD (${\mathrm{PRD}}_{703-868*}^{\mathrm{Strep}}$), and two constructs representing the N-terminal soluble portion and the C-terminal amyloidogenic portion of its PRD (${\mathrm{PRD}}_{703-800}^{\mathrm{Strep}}$ and ${\mathrm{PRD}}_{800-868}^{\mathrm{Strep}}$, respectively); the numbers signify ALIX residues, the asterisk denotes P801G mutation, and Strep indicates a C-terminal strep tag (*26*), which facilitated rapid protein purification using affinity chromatography and Alexa Fluor 488 labeling (Materials and Methods).

${\mathrm{ALIX}}_{1-868*}^{\mathrm{Strep}}$ was monomeric by analytical ultracentrifugation (AUC; fig. S2), consistent with a prior study that reported that ALIX made using insect cells was monomeric (*27*). Its solution, however, became turbid in the presence of a molecular crowder polyethylene glycol 4000 (PEG-4000), and subsequent examination by microscopy revealed its spherical condensates (Fig. 1D). To disaggregate fibrils, lyophilized ALIX-PRD constructs were dissolved in a basic (pH 10) buffer (*28*, *29*). Upon rapid dilution to physiological pH conditions (pH 7.5) in the presence of PEG-4000, ${\mathrm{PRD}}_{703-868*}^{\mathrm{Strep}}$ and its truncated counterpart, ${\mathrm{PRD}}_{800-868}^{\mathrm{Strep}}$, also condensed into droplets (Fig. 1E). In addition, ${\mathrm{PRD}}_{800-868}^{\mathrm{Strep}}$ formed condensates without PEG-4000 (fig. S3), suggesting that it is responsible for ALIX’s phase separation. In contrast, ${\mathrm{PRD}}_{703-800}^{\mathrm{Strep}}$ did not phase separate under any conditions tested, consistent with our prior observations that this portion of ALIX-PRD is non-amyloidogenic and highly soluble (*22*). The median diameters of condensates of ${\mathrm{ALIX}}_{1-868*}^{\mathrm{Strep}}$, ${\mathrm{PRD}}_{703-868*}^{\mathrm{Strep}}$, and ${\mathrm{PRD}}_{800-868}^{\mathrm{Strep}}$ (50 μM each) were ~5, ~1.5, and ~0.5 μm, respectively (Fig. 1F), establishing that ALIX condensates were larger than those of its PRD. Corresponding ALIX constructs without strep tags also formed condensates (fig. S4), ruling out the contribution of the strep tag in ALIX’s phase separation. Although we occasionally observed a fusion of freshly prepared ${\mathrm{ALIX}}_{1-868*}^{\mathrm{Strep}}$ droplets, the corresponding time scales were in minutes and resulted in the formation of uneven oblong structures (Fig. 1G), suggesting the presence of viscous liquids in these condensates. A varying degree of co-partitioning of amyloid-sensitive dye, thioflavin T (ThT), was detected in freshly prepared condensates, with low ThT partitioning in ${\mathrm{ALIX}}_{1-868*}^{\mathrm{Strep}}$ droplets versus a robust partitioning in condensates of ALIX-PRD constructs, ${\mathrm{PRD}}_{703-868*}^{\mathrm{Strep}}$ and ${\mathrm{PRD}}_{800-868}^{\mathrm{Strep}}$ (Fig. 1H and fig. S5), indicating the presence of amyloid fibrils in the latter cases. Such fibrils could be occasionally visualized in aged droplets of ${\mathrm{PRD}}_{703-868*}^{\mathrm{Strep}}$ using transmission electron microscopy (TEM; Fig. 1I). Little to no fluorescence recoveries after photobleaching (FRAPs) were observed for the freshly prepared condensates of ALIX constructs (Fig. 1J), which established their partially solid/gel-like character. About 40% average recovery (in 60 s) was observed for ${\mathrm{PRD}}_{800-868}^{\mathrm{Strep}}$ as opposed to ≤20% recoveries for ${\mathrm{PRD}}_{703-868*}^{\mathrm{Strep}}$ and ${\mathrm{ALIX}}_{1-868*}^{\mathrm{Strep}}$, indicating that the cycling between soluble and phase-separated states was relatively more hindered in larger ALIX constructs, perhaps due to their increased gelation stemming from the presence of additional intermolecular interactions. Biological condensates often exhibit such nondynamic phases, which are implicated in cellular and pathological processes (*13*). Note that because of the spherical morphology of condensates of ALIX constructs (see Fig. 1, D and E), there exists a possibility that these droplets are initially liquid-like but then rapidly transition into nondynamic phases within a few minutes of their preparation. Collectively, these results establish that recombinant ALIX phase separates under crowding conditions and the phase separation motif likely resides in the amyloidogenic portion of its PRD.

### Phase separation of ALIX in living cells

**Fig. 2.**
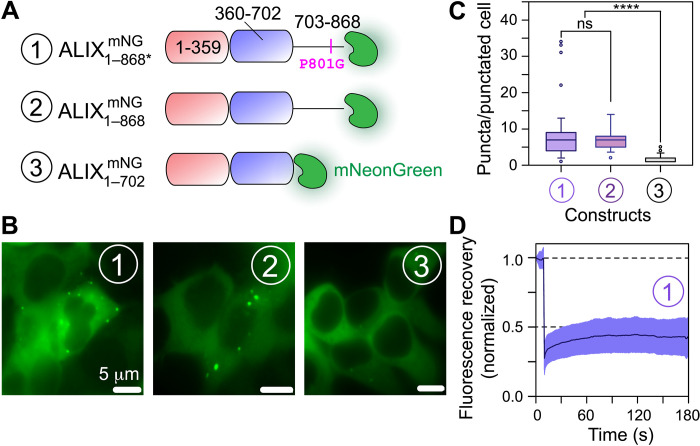
Phase separation of ALIX in HEK293T cells. (**A**) Schematic of ALIX constructs used for cellular experiments. Each construct is designated by a circled number and carries fluorescent protein, mNeonGreen (mNG), at the C terminus; all constructs lack the strep tag. (**B**) Representative microscopy images of HEK293T cells overexpressing the three ALIX constructs shown in (A) (also see fig. S7). For ${\mathrm{ALIX}}_{1-868*}^{\mathrm{mNG}}$ and ${\mathrm{ALIX}}_{1-868}^{\mathrm{mNG}}$ (constructs 1 and 2, respectively), about 20% protein population was colocalized in puncta, i.e., distinct fluorescence green spots (>300 representative cells, *n* = 5, for ${\mathrm{ALIX}}_{1-868*}^{\mathrm{mNG}}$; >200 cells, *n* = 3, for ${\mathrm{ALIX}}_{1-868}^{\mathrm{mNG}}$). For ${\mathrm{ALIX}}_{1-702}^{\mathrm{mNG}}$ lacking the PRD (construct 3), this phase separation is notably abrogated (about 10% of representative cells with puncta, >400 cells, *n* = 3). (**C**) Box plot of the number of puncta observed per cell for each ALIX construct shown in (A); only a representative population of cells containing puncta was used for analysis. (**D**) In-cell FRAP analysis of the condensates formed by ${\mathrm{ALIX}}_{1-868*}^{\mathrm{mNG}}$ (construct 1). Almost a complete lack of fluorescence recovery over time (~10% average recovery in 180 s) confirms nondynamic nature of cellular ALIX condensates. ns, not significant.

Next, we examined phase separation of ALIX in mammalian cells. Overexpression of ALIX_1–868*_ tagged with a fluorescent protein mNeonGreen (${\mathrm{ALIX}}_{1-868*}^{\mathrm{mNG}}$; Fig. 2A) (*30*) in human embryonic kidney (HEK) 293T cells resulted in the formation of submicrometer-scale puncta, which could be visualized using live-cell fluorescence microscopy (Fig. 2B and fig. S7). Puncta were observed in ~20% of the representative imaged cells, with a median value of ~8 puncta per punctated cell (Fig. 2C). To determine the impact of the P801G mutation on the phase separation of ALIX, similar experiments were performed with mNeonGreen-tagged wild-type ALIX (${\mathrm{ALIX}}_{1-868}^{\mathrm{mNG}}$), which also exhibited puncta formation with ~7 puncta per punctated cell (Fig. 2, B and C), demonstrating that ALIX’s phase separation is not influenced by the P801G mutation. In contrast, overexpression of mNeonGreen-tagged ALIX lacking the PRD (${\mathrm{ALIX}}_{1-702}^{\mathrm{mNG}}$) resulted in an almost complete loss of puncta formation (Fig. 2, B and C), confirming that the PRD of ALIX is necessary for its phase separation. In-cell FRAP experiments carried out on ${\mathrm{ALIX}}_{1-868*}^{\mathrm{mNG}}$ puncta corroborated our in vitro results with negligible fluorescence recovery (Fig. 2D), establishing their nondynamic nature. These results show that ALIX forms condensates in living cells and the PRD is required for its phase separation.

### Co-partitioning of CHMP4 paralogs in ALIX condensates

**Fig. 3.**
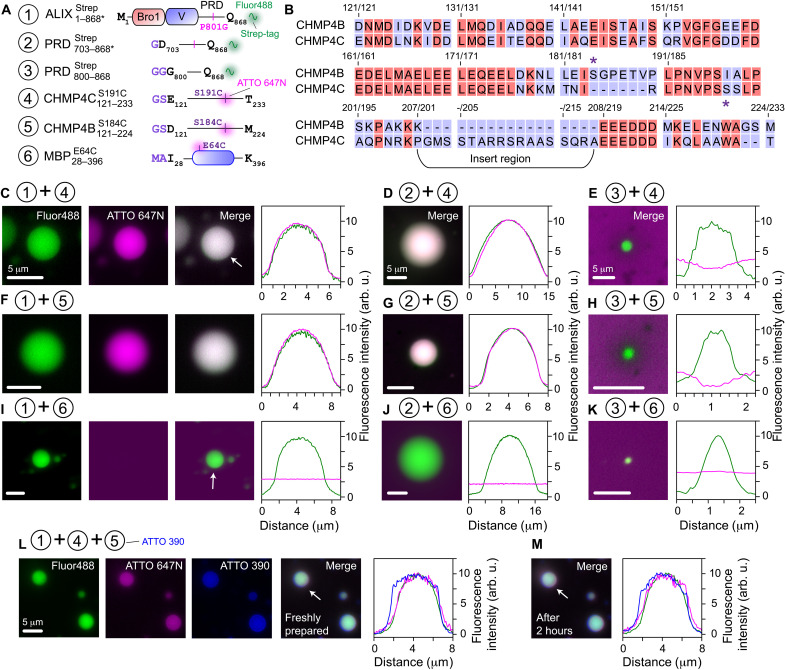
Colocalization of CHMP4 paralogs in condensates of ALIX constructs. (**A**) Scheme of constructs used for colocalization experiments. The positions of mutations and the fluorophore conjugation sites are marked. Non-native residues are in purple. ${\mathrm{MBP}}_{28-396}^{E64C}$ was used as a negative control. (**B**) Sequence comparison of the C-terminal portions of CHMP4B and CHMP4C. Asterisks denote the locations of engineered cysteine residues. The unique insert region of CHMP4C (residues 201 to 218) is marked. Representative microscopy images and fluorescence intensity profiles showing the colocalization (or the lack thereof) of ATTO-647N–labeled (**C** to **E**) $\mathrm{CHMP}4C_{121-233}^{S191C}$, (**F** to **H**) $\mathrm{CHMP}4B_{121-224}^{S184C}$, and (**I** to **K**) ${\mathrm{MBP}}_{28-396}^{E64C}$ in the condensates of Alexa Fluor 488–labeled ALIX constructs, ${\mathrm{ALIX}}_{1-868*}^{\mathrm{Strep}}$ (C), (F), and (I), ${\mathrm{PRD}}_{703-868*}^{\mathrm{Strep}}$ (D), (G), and (J), and ${\mathrm{PRD}}_{800-868}^{\mathrm{Strep}}$ (E), (H), and (K). Uniform co-partitioning of CHMP4 paralogs in condensates of ${\mathrm{ALIX}}_{1-868*}^{\mathrm{Strep}}$ and ${\mathrm{PRD}}_{703-868*}^{\mathrm{Strep}}$ was observed among all samples (*n* ≥ 3 with ≥100 condensates per sample); also see fig. S8 for panoramic images, fig. S9 for images at the respective fluorescent channels of droplets of ALIX-PRD constructs, and fig. S10 for colocalization of $\mathrm{CEP}55_{160-216}^{S215C}$ in ${\mathrm{PRD}}_{703-868*}^{\mathrm{Strep}}$ droplets. Representative microscopy images and fluorescence intensity profiles showing the colocalization of ATTO-647N–labeled $\mathrm{CHMP}4C_{121-233}^{S191C}$ and ATTO-390–labeled $\mathrm{CHMP}4B_{121-224}^{S184C}$ in (**L**) freshly prepared condensates of Alexa Fluor 488–labeled ${\mathrm{ALIX}}_{1-868*}^{\mathrm{Strep}}$ and (**M**) upon incubation at room temperature for 2 hours. White arrows mark the condensates used to generate the fluorescence intensity profiles. The individual proteins were mixed, followed by the addition of 5% (w/v) PEG-4000 to induce phase separation. Concentrations of ALIX constructs were 50 μM, and the partner proteins were at 1 μM. For ALIX constructs, the concentration of streptavidin Alexa Fluor 488 was 0.2 mg/ml. For partner proteins, the concentration of fluorescently labeled moieties was 5 mol %.

Since cellular ACBs contained CHMP4C and CHMP4B (*11*), we used their recombinant counterparts, namely, $\mathrm{CHMP}4C_{121-233}^{S191C}$ and $\mathrm{CHMP}4B_{121-224}^{S184C}$, to determine their co-partitioning in condensates of ALIX constructs (Fig. 3, A and B). Both CHMP4 constructs used here lacked their filament-forming core domains (residues 1 to 120) and carried a non-native cysteine residue that enabled labeling with fluorescent dyes. To assess co-partitioning of $\mathrm{CHMP}4C_{121-233}^{S191C}$ in ${\mathrm{ALIX}}_{1-868*}^{\mathrm{Strep}}$ condensates, the two proteins were mixed, and phase separation was induced by the addition of PEG-4000. $\mathrm{CHMP}4C_{121-233}^{S191C}$ colocalization in these condensates was immediate and readily visible by florescence microscopy (Fig. 3C and fig. S8A), suggesting that ALIX retained its structure in these condensates and could thus recruit its binding partners. Unexpectedly, $\mathrm{CHMP}4C_{121-233}^{S191C}$ also co-partitioned in ${\mathrm{PRD}}_{703-868*}^{\mathrm{Strep}}$ condensates (Fig. 3D and fig. S8B), whereas no such colocalization was observed in ${\mathrm{PRD}}_{800-868}^{\mathrm{Strep}}$ droplets (Fig. 3E and fig. S8C). Similar results, i.e., robust colocalization in ${\mathrm{ALIX}}_{1-868*}^{\mathrm{Strep}}$ and ${\mathrm{PRD}}_{703-868*}^{\mathrm{Strep}}$ condensates but no colocalization in ${\mathrm{PRD}}_{800-868}^{\mathrm{Strep}}$ droplets, were obtained using $\mathrm{CHMP}4B_{121-224}^{S184C}$ (Fig. 3, F and G, and fig. S8, A and C). These observations indicate that in addition to Bro1, ALIX harbors a second binding site for CHMP4 paralogs (see below), which likely resides in the N-terminal portion of its PRD (residues 703 to 800), and that the CHMP4 constructs tested here do not phase separate on their own under these experimental conditions. Experiments performed using an unrelated maltose-binding protein (${\mathrm{MBP}}_{28-396}^{E64C}$) that is not known to interact with ALIX demonstrated that it did not colocalize in condensates of ALIX constructs (Fig. 3, I to K), establishing that the colocalization of CHMP4 proteins in ${\mathrm{ALIX}}_{1-868*}^{\mathrm{Strep}}$ condensates stems from their specific association with ALIX. In addition, the two CHMP4 paralogs could simultaneously co-partition inside ${\mathrm{ALIX}}_{1-868*}^{\mathrm{Strep}}$ condensates (Fig. 3L). The latter showed minimal changes in respective fluorescence intensities after 2 hours (Fig. 3M), demonstrating that CHMP4 proteins remained confined within these condensates. Thus, we argue that ALIX’s ability to phase separate and selectively recruit CHMP4 paralogs will likely be vital contributing factors to the biogenesis and stability of cellular ACBs, explaining prior in vivo observations that showed that depletion of ALIX resulted in ~50% reduction in their volume (*11*).

### NMR analysis of ALIX-CHMP4 interactions

**Fig. 4.**
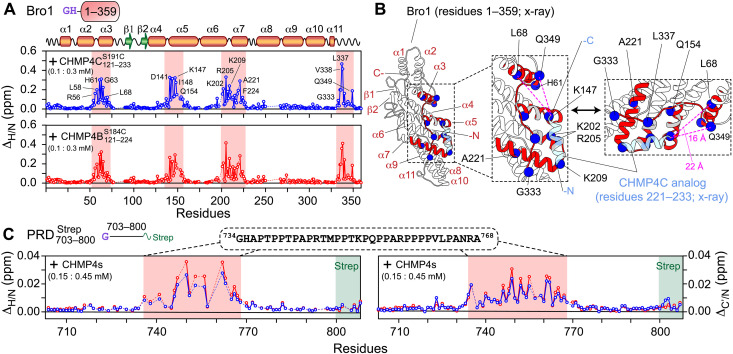
NMR analysis of ALIX-CHMP4 interactions. (**A**) ^1^H_N_/^15^N chemical shift perturbation profiles of 100 μM ^15^N/^2^H-labeled Bro1 on addition of 300 μM unlabeled CHMP4 constructs; $\mathrm{CHMP}4C_{121-233}^{S191C}$ (top, blue) and $\mathrm{CHMP}4B_{121-224}^{S184C}$ (bottom, red). Secondary structure elements and the scheme of Bro1 construct (fig. S6) used in these experiments are indicated above the panel. Red rectangles indicate regions that exhibit large chemical shift perturbations; a few of the affected residues are labeled. Dashed lines indicate proline residues or residues that could not be assigned unambiguously. (**B**) Ribbon diagram of the x-ray structure of the complex between Bro1 (white ribbons) and CHMP4C peptide analog (light blue ribbons) (*10*). Bro1 motifs that undergo large chemical shift changes in the presence of $\mathrm{CHMP}4C_{121-233}^{S191C}$ (A, top) are marked in red ribbons. A few affected residues are depicted by blue spheres. Gray ribbons indicate residues around the interaction site that could not be assigned unambiguously. Dashed magenta lines mark the distances between two representative Bro1 residues (L68 and Q349) and the C terminus of the CHMP4C peptide analog. (**C**) ^1^H_N_/^15^N and ^13^C′/^15^N chemical shift perturbation profiles of 150 μM ^15^N/^13^C-labeled ${\mathrm{PRD}}_{703-800}^{\mathrm{Strep}}$ in the presence of 450 μM unlabeled CHMP4 constructs [same color scheme as (A)]. The scheme of ${\mathrm{PRD}}_{703-800}^{\mathrm{Strep}}$ (fig. S6) and the primary sequence of the affected region (residues 734 to 768) marked by semitransparent red rectangles are shown above the graphs. The position of the C-terminal strep tag (residues 801 to 808) is denoted by semitransparent green rectangles. All NMR experiments were performed at 30°C in 20 mM sodium phosphate (pH 6.5), 1 mM TCEP, and 2 mM EDTA at a spectrometer ^1^H frequency of 800 MHz.

To investigate ALIX-CHMP4 interactions, NMR titration experiments were performed where three-molar equivalents of unlabeled $\mathrm{CHMP}4C_{121-233}^{S191C}$ or $\mathrm{CHMP}4B_{121-224}^{S184C}$ were added to ^15^N/^2^H-labeled Bro1; corresponding experiments using ${\mathrm{ALIX}}_{1-868*}^{\mathrm{Strep}}$ were not feasible owing to its large molecular size (~100 kDa), which resulted in severe line broadening. Using NMR spectroscopy, we previously established that Bro1 retains its fold in solution (*23*). On the addition of CHMP4 proteins, large ^1^H_N_/^15^N chemical shift perturbations [Δ_H/N_ ∼ 0.05 to 0.47 parts per million (ppm)] were observed for residues 54 to 72, 135 to 156, 201 to 227, and 333 to 349 of Bro1 (Fig. 4A and fig. S11). Figure 4B maps these perturbations onto the x-ray structure of the Bro1-CHMP4C peptide complex (*10*). Although the CHMP4C constructs used for these two studies were different, extended fragment for NMR (residues 121 to 233) versus a peptide analog for x-ray (residues 221 to 233), there was an excellent agreement between their results as most chemical shift perturbations were localized in and around the x-ray binding interface between Bro1 and the CHMP4C peptide. However, two Bro1 regions that showed large perturbations with $\mathrm{CHMP}4C_{121-233}^{S191C}$ (residues 54 to 72 and 347 to 349) were located ~15 to 25 Å away from the C terminus of the CHMP4C peptide (see Fig. 4B). These observations suggest that in addition to the binding site comprising the extreme C-terminal CHMP4C motif (residues 221 to 233), Bro1 binds to additional motif(s) present in residues 121 to 220 of $\mathrm{CHMP}4C_{121-233}^{S191C}$. Because ^1^H_N_/^15^N chemical shift perturbations in Bro1 resonances upon the addition of $\mathrm{CHMP}4B_{121-224}^{S184C}$ were similar to those obtained using $\mathrm{CHMP}4C_{121-233}^{S191C}$ (see Fig. 4A) and since the C-terminal motifs of CHMP4B (residues 207 to 224) and CHMP4C (residues 221 to 233) bind to the same Bro1 site as determined by x-ray crystallography (*3*), we conclude that Bro1 also binds to additional motif(s) localized in residues 121 to 206 of $\mathrm{CHMP}4B_{121-224}^{S184C}$.

To uncover the existence of interactions between the N-terminal portion of ALIX-PRD (residues 703 to 800) and CHMP4 paralogs, as suggested by our microscopy results (see Fig. 3, C to H), we performed titration experiments using unlabeled CHMP4 proteins and NMR-visible ${\mathrm{PRD}}_{703-800}^{\mathrm{Strep}}$. This region of ALIX-PRD is unstructured in its free form (*22*). The addition of three-molar equivalent of $\mathrm{CHMP}4C_{121-233}^{S191C}$ or $\mathrm{CHMP}4B_{121-224}^{S184C}$ resulted in small but noticeable ^1^H_N_/^15^N and ^13^C′/^15^N chemical shift changes in ${\mathrm{PRD}}_{703-800}^{\mathrm{Strep}}$ resonances, Δ_H/N_ and Δ_C′/N_ of 0.01 to 0.04 and 0.01 to 0.03 ppm, respectively (Fig. 4C and figs. S12 and S13). The affected PRD region encompassed residues 734 to 768, which overlaps with Src kinase binding site on PRD [residues 752 to 757; see Fig. 1A (*19*)]. This motif is highly basic owing to multiple arginine residues [theoretical isoelectric point (pI) (*31*), ~12]. In contrast, both CHMP4 proteins used in this work are anionic (theoretical pI, 4 to 5). Since NMR experiments were performed at pH 6.5 (Materials and Methods), ${\mathrm{PRD}}_{703-800}^{\mathrm{Strep}}$ likely binds to CHMP4 proteins via electrostatic interactions. Because the ^1^H_N_/^15^N chemical shift changes observed for ${\mathrm{PRD}}_{703-800}^{\mathrm{Strep}}$-CHMP4 interactions were smaller than those observed for Bro1-CHMP4 interactions, we conclude that the binding between ${\mathrm{PRD}}_{703-800}^{\mathrm{Strep}}$ and CHMP4 paralogs is weaker than Bro1-CHMP4 association, and that CHMP4-bound ${\mathrm{PRD}}_{703-800}^{\mathrm{Strep}}$ remains disordered. Together, NMR titration experiments uncovered the existence of additional interactions between ALIX domains (Bro1 and PRD) and CHMP4 constructs.

### Conformation and dynamics of CHMP4 paralogs in solution

**Fig. 5.**
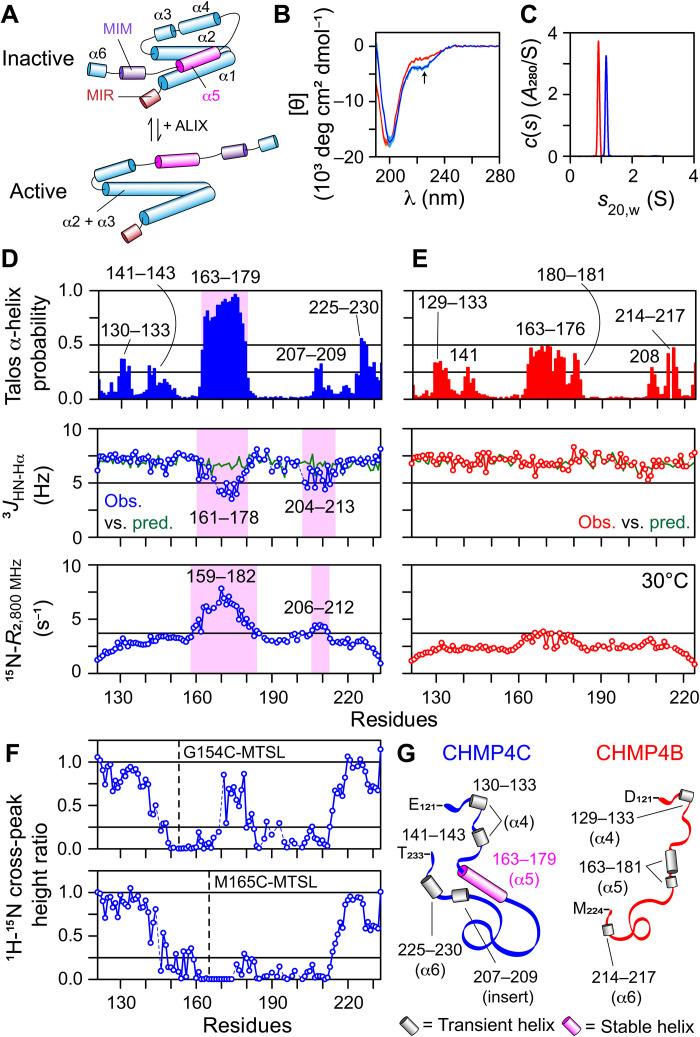
Conformation and dynamics of CHMP4 paralogs in solution. (**A**) Hypothetical models of CHMP4 (*2*, *4*, *32*, *33*), originally described by Vietri *et al.* (*2*). MIM, MIT-interacting motif, which binds to the MIT domain containing proteins (e.g., VPS4) (*72*); MIR, membrane insertion region. Helix α5 (pink) is likely responsible for CHMP4 autoinhibition. (**B**) Far-UV CD spectra (five scans), mean (solid line) and SD (shaded region), and (**C**) sedimentation profiles of $\mathrm{CHMP}4C_{121-233}^{S191C}$ (blue) and $\mathrm{CHMP}4B_{121-224}^{S184C}$ (red). Arrow points to the dip at ~222 nm in the CD spectrum of $\mathrm{CHMP}4C_{121-233}^{S191C}$. NMR analysis of (**D**) $\mathrm{CHMP}4C_{121-233}^{S191C}$ and (**E**) $\mathrm{CHMP}4B_{121-224}^{S184C}$, including TALOS-N–derived helical propensities (top), a comparison between experimental ^3^*J*_HN-Hα_ couplings against random coil values (middle), and ^15^N-*R*_2_ profiles measured at 30°C (table S1). Unlike $\mathrm{CHMP}4B_{121-224}^{S184C}$, $\mathrm{CHMP}4C_{121-233}^{S191C}$ showed greater helical propensity for residues 163 to 179, highlighted by semitransparent magenta rectangle. The corresponding drop in ^3^*J*_HN-Hα_ couplings and elevated ^15^N-*R*_2_ values of this region confirmed the presence of a stable helix. The insert region of $\mathrm{CHMP}4C_{121-233}^{S191C}$ (see Fig. 3B) exhibited deviations from random coil ^3^*J*_HN-Hα_ couplings and elevated ^15^N-*R*_2_ values (highlighted by semitransparent magenta rectangles), indicating a residual helical structure. Residues of the regions showing elevated TALOS-N–derived helical propensities (ranging between 0.25 and 0.56), indicating transient helical structures, are labeled. (**F**) Evidence of transient long-range interactions in CHMP4C using experimental PRE profiles of $\mathrm{CHMP}4C_{121-233}^{G154C}$ (top) and $\mathrm{CHMP}4C_{121-233}^{M165C}$ (bottom). The locations of paramagnetic label, MTSL, are marked with dashed lines. (**G**) Schematic of $\mathrm{CHMP}4C_{121-233}^{S191C}$ (blue) and $\mathrm{CHMP}4B_{121-224}^{S184C}$ (red) based on NMR results. Transient and stable helices are depicted as gray and magenta cylinders, respectively; numbers denote the corresponding residues. The CHMP4 helices based on the proposed models of full-length proteins [see (A)] are in parentheses.

To further explore interactions between CHMP4 paralogs and ALIX, we first analyzed $\mathrm{CHMP}4C_{121-233}^{S191C}$ and $\mathrm{CHMP}4B_{121-224}^{S184C}$ in their free forms. The structures of CHMP4 paralogs are not available as they are refractory to crystallography. On the basis of the structures of homologs and sequence-based and AlphaFold predictions, the CHMP4 paralogs are suggested to comprise six helices (α1 to α6; Fig. 5A and fig. S14), with α1 to α3 spanning their filament-forming N-terminal core domains (residues 1 to 120) and α4 to α6 localized in their C-terminal tails (*2*, *4*, *32*–*34*). Cytosolic CHMP4 proteins are hypothesized to be in a closed conformation because of intramolecular interactions between α5 and α1-α2. In the presence of activation factors (e.g., ALIX), CHMP4 paralogs likely undergo a conformational rearrangement, involving the dissociation of α5 from α1-α2, followed by merging α2 with α3, and a reorganization of their C termini, triggering their subsequent polymerization. Unexpectedly, far-ultraviolet (UV) circular dichroism (CD) spectra of $\mathrm{CHMP}4C_{121-233}^{S191C}$ and $\mathrm{CHMP}4B_{121-224}^{S184C}$ showed that both were largely disordered (Fig. 5B). However, a slight dip at ~222-nm UV wavelength indicated that $\mathrm{CHMP}4C_{121-233}^{S191C}$ exhibited a greater helical propensity than $\mathrm{CHMP}4B_{121-224}^{S184C}$. These results were confirmed by AUC experiments (Fig. 5C), which established that these two constructs yielded notably different best-fit frictional ratios (1.8 and 2.3 for $\mathrm{CHMP}4C_{121-233}^{S191C}$ and $\mathrm{CHMP}4B_{121-224}^{S184C}$, respectively), despite being different by only ~1 kDa. Consequently, both proteins were monomeric, with no indication of self-association, and although both were largely disordered, $\mathrm{CHMP}4C_{121-233}^{S191C}$ exhibited a more compact conformation than $\mathrm{CHMP}4B_{121-224}^{S184C}$.

Both $\mathrm{CHMP}4C_{121-233}^{S191C}$ and $\mathrm{CHMP}4B_{121-224}^{S184C}$ yielded high-quality ^1^H-^15^N transverse relaxation optimized spectroscopy–heteronuclear single quantum coherence (TROSY-HSQC) spectra, and a narrow dispersion of chemical shifts of their backbone amide proton resonances (7.5 to 8.8 ppm) indicated disordered conformations (fig. S15). To further explore their conformational propensities, we recorded backbone chemical shifts, ^3^*J*_HN-Hα_ couplings, and ^15^N-transverse relaxation rates (*R*_2_). Secondary chemical shifts (Δδ), the differences between experimental and the corresponding predicted random coil values (*35*, *36*), are shown in fig. S16. The region between residues 163 and 179 of $\mathrm{CHMP}4C_{121-233}^{S191C}$ that corresponds to the predicted α5 motif in full-length CHMP4C exhibited elevated Δδ(^13^C_α_) values (0.5 to 1.5 ppm), indicating a stable helical conformation in this region; Δδ(^13^C_α_) values are the best reporters of local secondary structure. In contrast, Δδ(^13^C_α_) values of $\mathrm{CHMP}4B_{121-224}^{S184C}$ were evenly distributed ~0 ppm, indicative of a random coil conformation. To further examine these conformational differences, the secondary structures of $\mathrm{CHMP}4C_{121-233}^{S191C}$ and $\mathrm{CHMP}4B_{121-224}^{S184C}$ were determined from experimental backbone chemical shifts using TALOS-N (Fig. 5, D and E, top) (*37*). The helical propensity for residues 163 to 179 of $\mathrm{CHMP}4C_{121-233}^{S191C}$ ranged between 0.6 and 0.96, confirming a stable helix in this region. In contrast, the helical propensity of residues 163 to 181 in $\mathrm{CHMP}4B_{121-224}^{S184C}$ was 0.13 to 0.49, indicative of a transient helical character, although the primary sequence of this region is nearly identical to that of $\mathrm{CHMP}4C_{121-233}^{S191C}$ (see Fig. 3B); helical propensities of residues 177 to 179 were 0.13 to 0.22, suggesting a possible bend at this location. These observations were complemented by experimental ^3^*J*_HN-Hα_ couplings (Fig. 5D, middle), which showed largely helical values ranging between 3.5 and 5.5 Hz for residues 161 to 178 of $\mathrm{CHMP}4C_{121-233}^{S191C}$ that deviated considerably from the corresponding random coil values (~7 Hz) predicted using nearest-neighbor effects (*38*). In addition, the ^15^N-*R*_2_ values of residues 159 to 182, measured at 800 MHz at 30°C, were uniformly elevated (6 to 8 s^−1^; Fig. 5D, bottom), which established an ordered conformation of this motif. In contrast, residues 163 to 181 of $\mathrm{CHMP}4B_{121-224}^{S184C}$ exhibited nonhelical ^3^*J*_HN-Hα_ couplings (~7 Hz) and slightly elevated ^15^N-*R*_2_ values (2 to 4 s^−1^), indicating a lack of stable helical configuration (Fig. 5E). Residues 130 to 133 and 140 to 143 (α4), 207 to 209 (insert), and 225 to 230 (α6) of $\mathrm{CHMP}4C_{121-233}^{S191C}$ exhibited helical propensities ranging between 0.25 and 0.56 (Fig. 5D, top), indicating that these motifs have lower propensities for spontaneous helix formation than the region between residues 163 and 179 (α5); the labels in parentheses denote the corresponding proposed regions in full-length CHMP4C. Most notable among these were a few residues of the insert region of $\mathrm{CHMP}4C_{121-233}^{S191C}$ that showed helical ^3^*J*_HN-Hα_ couplings (~5 Hz; Fig. 5D, middle), slightly elevated ^15^N-*R*_2_ values (~4 s^−1^; Fig. 5D, bottom), and Δδ(^13^C_α_) shifts (~0.3 ppm; fig. S16A), indicating a partially ordered helix. This insert is highly basic owing to the presence of multiple arginine residues (theoretical pI, ~12.5). It therefore may transiently interact with other acidic motifs of $\mathrm{CHMP}4C_{121-233}^{S191C}$, e.g., residues 163 to 179 (theoretical pI, ~3.7), stabilizing their conformation, and that the absence of this insert in $\mathrm{CHMP}4B_{121-224}^{S184C}$ may result in a lack of a stable helix in the region between residues 163 and 181. To confirm these long-range contacts, we performed intramolecular paramagnetic relaxation enhancement (PRE) experiments (*39*–*41*) on two CHMP4C mutants, $\mathrm{CHMP}4C_{121-233}^{G154C}$ and $\mathrm{CHMP}4C_{121-233}^{M165C}$ (Fig. 5F and fig. S17). The attachment of a paramagnetic nitroxide spin label, (1-oxyl-2,2,5,5-tetramethyl-Δ3-pyrroline-3-methyl) methanethiosulfonate (MTSL), at individual residues C154 and C165 resulted in local signal attenuation and a notable attenuation in the ^1^H_N_/^15^N cross-peaks of residues 200 to 212, confirming the transient long-range interactions between the acidic residues encompassing the α5 motif of CHMP4C and the basic residues of its insert. These findings are consistent with our AUC data (see Fig. 5C), which showed that among the two CHMP4 paralogs, CHMP4C is more compact. For $\mathrm{CHMP}4B_{121-224}^{S184C}$, in addition to motif 163 to 181 (α5), residues 129 to 133 (α4) and 217 to 217 (α6) showed helical propensities ranging between 0.25 and 0.5 (Fig. 5E, top), indicating transient helices. Thus, both CHMP4 paralogs tested here are largely disordered with residual helical propensities (Fig. 5G), apart from the α5 motif of CHMP4C that forms a stable helix and the presence of transient long-range interactions between the basic residues of its insert and acidic residues in and around its α5 motif. Disordered proteins or regions are vital signaling hubs, as their lack of structure facilitates dynamic protein-protein interactions of modest affinity but high specificity (*42*, *43*). We argue that disordered C-terminal tails of CHMP4 paralogs will provide multiple interaction motifs and facilitate the formation of metastable CHMP4 filaments essential for membrane scission.

### Mechanism of ALIX-mediated CHMP4 activation

**Fig. 6.**
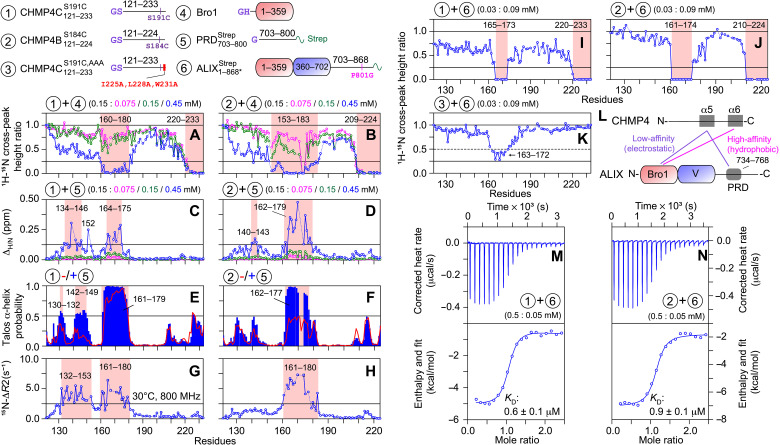
NMR and ITC analyses of CHMP4-ALIX interactions. (Top) Schemes of constructs used in these experiments. The reduction in ^1^H_N_/^15^N cross-peak heights of 150 μM ^15^N-labeled (**A**) $\mathrm{CHMP}4C_{121-233}^{S191C}$ and (**B**) $\mathrm{CHMP}4B_{121-224}^{S184C}$ as a function of increasing amount of unlabeled Bro1. The color scheme is as follows: CHMP4 fragments + Bro1 molar ratio = 1:0.5 (magenta), 1:1 (green), and 1:3 (blue). The perturbations in ^1^H_N_/^15^N chemical shifts of 150 μM ^15^N-labeled (**C**) $\mathrm{CHMP}4C_{121-233}^{S191C}$ and (**D**) $\mathrm{CHMP}4B_{121-224}^{S184C}$ as a function of increasing amount of unlabeled ${\mathrm{PRD}}_{703-800}^{\mathrm{Strep}}$ [same color scheme as (A) and (B)]. Dashed lines indicate proline residues or residues that could not be assigned unambiguously. TALOS-N–derived helical propensities of (**E**) $\mathrm{CHMP}4C_{121-233}^{S191C}$ and (**F**) $\mathrm{CHMP}4B_{121-224}^{S184C}$, in the presence (blue bars) and absence (red lines; also see Fig. 5, D and E, top) of ${\mathrm{PRD}}_{703-800}^{\mathrm{Strep}}$. Missing residues are shown as gaps. ^15^N-Δ*R*_2_ profiles measured on (**G**) $\mathrm{CHMP}4C_{121-233}^{S191C}$ + ${\mathrm{PRD}}_{703-800}^{\mathrm{Strep}}$ and (**H**) $\mathrm{CHMP}4B_{121-224}^{S184C}$ + ${\mathrm{PRD}}_{703-800}^{\mathrm{Strep}}$ samples at 800 MHz (30°C); ^15^N-Δ*R*_2_, lifetime line broadening (*44*, *45*), are the differences observed in ^15^N-*R*_2_ values of CHMP4 fragments recorded with and without ${\mathrm{PRD}}_{703-800}^{\mathrm{Strep}}$. Protein concentrations were 150 μM ^15^N-labeled CHMP4 paralogs and 450 μM unlabeled ${\mathrm{PRD}}_{703-800}^{\mathrm{Strep}}$; also see Fig. 5 (D and E, bottom) for ^15^N-*R*_2_ values of CHMP4 fragments recorded in the absence of ${\mathrm{PRD}}_{703-800}^{\mathrm{Strep}}$. The reduction in ^1^H_N_/^15^N cross-peak heights of 30 μM ^15^N/^2^H-labeled (**I**) $\mathrm{CHMP}4C_{121-233}^{S191C}$, (**J**) $\mathrm{CHMP}4B_{121-224}^{S184C}$, and (**K**) $\mathrm{CHMP}4C_{121-233}^{S191C,\mathrm{AAA}}$ in the presence of 90 μM unlabeled ${\mathrm{ALIX}}_{1-868*}^{\mathrm{Strep}}$. In all panels, the affected regions are highlighted with semitransparent red rectangles. (**L**) Scheme summarizing ALIX-CHMP4 interactions found in this study. ITC analyses of the titrations of 500 μM (**M**) $\mathrm{CHMP}4C_{121-233}^{S191C}$ and (**N**) $\mathrm{CHMP}4B_{121-224}^{S184C}$ into 50 μM ${\mathrm{ALIX}}_{1-868*}^{\mathrm{Strep}}$; also see table S2.

To further explore CHMP4-ALIX interactions, we performed NMR titration experiments where increasing concentrations of Bro1 or ${\mathrm{PRD}}_{703-800}^{\mathrm{Strep}}$ were added to NMR-visible CHMP4 constructs. Reductions in ^1^H_N_/^15^N cross-peak heights of 150 μM ^15^N-labeled CHMP4 proteins were observed with unlabeled Bro1 (Fig. 6, A and B, and fig. S18, A and B). Specifically, the cross-peaks of the α6 motifs of $\mathrm{CHMP}4C_{121-233}^{S191C}$ and $\mathrm{CHMP}4B_{121-224}^{S184C}$ (residues 220 to 233 and 209 to 224, respectively) were completely attenuated in the presence of 75 μM Bro1, indicating that these motifs represent high-affinity Bro1-binding sites. These results agree with a study that showed that residues 221 to 233 of CHMP4C and residues 207 to 224 of CHMP4B bind to Bro1 (*3*). Unexpectedly, upon a stepwise increase in Bro1 concentration (75, 150, and 450 μM of Bro1; Fig. 6, A and B), the cross-peaks of residues 160 to 180 (α5) of $\mathrm{CHMP}4C_{121-233}^{S191C}$ and residues 153 to 183 (α5) of $\mathrm{CHMP}4B_{121-224}^{S184C}$ were progressively attenuated, establishing that these α5 motifs represent low-affinity sites for Bro1. These results are in excellent agreement with the above observations that showed that residues 54 to 72 and 347 to 349 of Bro1 bind to motifs located in the N-terminal portions of CHMP4 constructs (see Fig. 4, A and B). TALOS-N–derived helical propensities obtained from the backbone chemical shifts of CHMP4 paralogs in the presence of Bro1 are shown in fig. S19 (A and B). Extensive line broadening precluded a detailed analysis of the helical propensities of the high- and low-affinity CHMP4 sites in their Bro1-bound forms, and no noticeable changes were observed in regions that did not bind to Bro1. However, a gradual increase in ^15^N-Δ*R*_2_ values of residues surrounding the high- and low-affinity sites indicates that these motifs are likely ordered in the presence of Bro1 (fig. S19, C and D). Note that the high-affinity sites (α6 motifs) of CHMP4 paralogs form ordered helices with Bro1 as evidenced by x-ray crystallography (see Fig. 1A) (*3*) and that ^15^N-Δ*R*_2_ or lifetime line broadening (*44*, *45*) are the differences observed in ^15^N-*R*_2_ values of CHMP4 fragments with and without Bro1.

The titration experiments performed on 150 μM ^15^N-labeled CHMP4 proteins and unlabeled ${\mathrm{PRD}}_{703-800}^{\mathrm{Strep}}$ (75, 150, and 450 μM) yielded negligible ^1^H_N_/^15^N cross-peak attenuation but large chemical shift perturbations, especially with 450 μM ${\mathrm{PRD}}_{703-800}^{\mathrm{Strep}}$, a manifestation of fast exchange on the chemical shift time scale (Δ_H/N_ ~ 0.13 to 0.5 ppm; Fig. 6, C and D, and fig. S18, C and D). These chemical shift changes were considerably larger than the small perturbations observed for samples comprising 150 μM ^15^N-labeled ${\mathrm{PRD}}_{703-800}^{\mathrm{Strep}}$ and 450 μM unlabeled CHMP4 paralogs (see Fig. 4C), indicating that although CHMP4-bound ${\mathrm{PRD}}_{703-800}^{\mathrm{Strep}}$ remains disordered, the CHMP4 motifs that bind to ${\mathrm{PRD}}_{703-800}^{\mathrm{Strep}}$ form ordered bound conformations. The large chemical shift changes (Δ_H/N_ ≥ 0.125 ppm) observed in samples comprising 150 μM CHMP4 proteins and 450 μM ${\mathrm{PRD}}_{703-800}^{\mathrm{Strep}}$ were localized at residues 134 to 146, 152, and 164 to 175 of $\mathrm{CHMP}4C_{121-233}^{S191C}$ and residues 140 to 143 and 162 to 179 of $\mathrm{CHMP}4B_{121-224}^{S184C}$ (see Fig. 6, C and D). In comparison, the C-terminal portions, residues 180 onward, of both CHMP4 constructs showed minimal perturbations. A noticeable increase in TALOS-N–derived helical propensities (≥0.5) for residues 130 to 132 and 142 to 149 (α4) of $\mathrm{CHMP}4C_{121-233}^{S191C}$ and residues 162 to 177 (α5) of $\mathrm{CHMP}4B_{121-224}^{S184C}$ was observed with ${\mathrm{PRD}}_{703-800}^{\mathrm{Strep}}$ (Fig. 6, E and F). Furthermore, residues of the α5 motif of $\mathrm{CHMP}4C_{121-233}^{S191C}$ that formed a stable helix in free form (see Fig. 5D, top) also displayed increased helicity in the presence of ${\mathrm{PRD}}_{703-800}^{\mathrm{Strep}}$, especially for residues 163 to 170 (helical propensity, ~1). No marked changes in helicity were observed for the C-terminal portions, residues 180 onward, of either CHMP4 constructs in the presence of ${\mathrm{PRD}}_{703-800}^{\mathrm{Strep}}$. Elevated ^15^N-Δ*R*_2_ values (≥2.5 s^−1^) were obtained for residues 132 to 153 (α4) and 161 to 180 (α5) of $\mathrm{CHMP}4C_{121-233}^{S191C}$ and residues 161 to 180 (α5) of $\mathrm{CHMP}4B_{121-224}^{S184C}$ in the presence of ${\mathrm{PRD}}_{703-800}^{\mathrm{Strep}}$ (Fig. 6, G and H), establishing an increased ordering of their bound conformations. Therefore, in contrast to $\mathrm{CHMP}4B_{121-224}^{S184C}$ where the binding to ${\mathrm{PRD}}_{703-800}^{\mathrm{Strep}}$ is localized around the α5 motif, the binding interface involves a substantially larger N-terminal portion of $\mathrm{CHMP}4C_{121-233}^{S191C}$, namely, α4 and α5 motifs, perhaps because folding of α5 helix is a prerequisite for the interactions of CHMP4 paralogs with ALIX-PRD. Because residues 734 to 768 of ${\mathrm{PRD}}_{703-800}^{\mathrm{Strep}}$ exhibit chemical shift changes with CHMP4 proteins (see Fig. 4C), we conclude that residues 734 to 768 of ALIX-PRD bind to the abovementioned N-terminal CHMP4 motifs. These results establish a large gain in helicity and structural order for CHMP4 paralogs upon their interactions with ALIX-PRD. To determine whether residual CHMP4 helices play a role in binding to ALIX-PRD, a phenomenon known as conformational selection or folding before binding (*46*–*48*) would require an extensive kinetic analysis, which is ongoing in our laboratory.

The results of titration experiments performed on samples comprising 30 μM ^15^N/^2^H-labeled CHMP4 constructs and 90 μM unlabeled ${\mathrm{ALIX}}_{1-868*}^{\mathrm{Strep}}$ are shown in Fig. 6 (I and J); unlike measurements mentioned above, these experiments were carried out at lower concentrations to account for the increase in viscosity with ${\mathrm{ALIX}}_{1-868*}^{\mathrm{Strep}}$. A complete attenuation of ^1^H_N_/^15^N cross-peak heights of residues 165 to 173 (α5) and 220 to 233 (α6) of $\mathrm{CHMP}4C_{121-233}^{S191C}$ and residues 161 to 174 (α5) and 210 to 224 (α6) of $\mathrm{CHMP}4B_{121-224}^{S184C}$ was observed with ${\mathrm{ALIX}}_{1-868*}^{\mathrm{Strep}}$. These results complement the above findings and confirm that ALIX binds to both α5 and α6 motifs of CHMP4 paralogs. To gain mechanistic insights, we generated an additional CHMP4C construct, $\mathrm{CHMP}4C_{121-233}^{S191C,\mathrm{AAA}}$, that carried three alanine substitutions in its α6 motif (I225A, L228A, and W231A; Fig. 6, top). A single alanine substitution for each of these three highly conserved hydrophobic residues was previously shown to abolish α6 (CHMP4)–Bro1 (ALIX) interactions (*3*). The results of NMR titration experiments carried out using 30 μM ^15^N/^2^H-labeled $\mathrm{CHMP}4C_{121-233}^{S191C,\mathrm{AAA}}$ with and without 90 μM unlabeled ${\mathrm{ALIX}}_{1-868*}^{\mathrm{Strep}}$ are shown in Fig. 6K and fig. S20. As expected, the cross-peaks of the α6 motif of $\mathrm{CHMP}4C_{121-233}^{S191C,\mathrm{AAA}}$ did not show any signal attenuation or chemical shift perturbations in the presence of ${\mathrm{ALIX}}_{1-868*}^{\mathrm{Strep}}$. Resonances of residues 163 to 172 (α5), however, underwent signal attenuation, albeit to a lower extent as compared to $\mathrm{CHMP}4C_{121-233}^{S191C}$ + ${\mathrm{ALIX}}_{1-868*}^{\mathrm{Strep}}$ titration. These observations establish that, in the absence of α6-${\mathrm{ALIX}}_{1-868*}^{\mathrm{Strep}}$ hydrophobic interactions, the anionic α5 motif of CHMP4C can independently bind to ALIX. Thus, we propose that ALIX and full-length CHMP4 interactions will comprise hydrophobic (high-affinity) and electrostatic (low-affinity) associations, involving the α6 and α5 motifs of CHMP4 paralogs and Bro1 and PRD of ALIX (Fig. 6L). These intermolecular interactions will, in turn, prevent the intramolecular association between α5 and the N-terminal CHMP4 motifs (α1 and α2), thereby triggering the cascade of conformational changes resulting in CHMP4 activation (see Fig. 5A). The thermodynamics of binding between CHMP4 paralogs and ${\mathrm{ALIX}}_{1-868*}^{\mathrm{Strep}}$ were characterized by isothermal titration calorimetry (ITC; Fig. 6, M and N). Excellent agreement was obtained by fitting the experimental isotherms to an independent site binding model in the software NanoAnalyze (TA Instruments), yielding the equilibrium dissociation constants, *K*_D_, of 0.6 ± 0.1 μM for $\mathrm{CHMP}4C_{121-233}^{S191C}$-${\mathrm{ALIX}}_{1-868*}^{\mathrm{Strep}}$ and 0.9 ± 0.1 μM for $\mathrm{CHMP}4B_{121-224}^{S184C}$-${\mathrm{ALIX}}_{1-868*}^{\mathrm{Strep}}$ interactions (table S2). Experimentally determined values of the parameter *n* (where *n* is the number of binding sites) were very close to unity, which established a 1:1 stoichiometry of these interactions. Similar *K*_D_ values were obtained for the interactions of Bro1 with $\mathrm{CHMP}4C_{121-233}^{S191C}$ and $\mathrm{CHMP}4B_{121-224}^{S184C}$ (0.9 ± 0.1 and 1.0 ± 0.2 μM, respectively; table S2), implying that the association of CHMP4 paralogs and ALIX is primarily governed by the Bro1 domain and that the interactions with ALIX-PRD are considerably weaker, consistent with the above NMR results. To determine the impact of salt on these interactions, we carried out additional ITC measurements in the presence of 150 mM NaCl, yielding a *K*_D_ of 6.1 ± 0.7 μM for $\mathrm{CHMP}4C_{121-233}^{S191C}$-${\mathrm{ALIX}}_{1-868*}^{\mathrm{Strep}}$ interactions (table S2). Similar ITC measurements carried out in the presence of 150 mM NaCl did not detect any noticeable $\mathrm{CHMP}4C_{121-233}^{S191C,\mathrm{AAA}}$-${\mathrm{ALIX}}_{1-868*}^{\mathrm{Strep}}$ interactions, which further confirmed the electrostatic association between the anionic α5 motif of CHMP4C and ALIX (table S2). Overall, the above measured *K*_D_ values are notably stronger than those reported in a previous study (*3*), which used surface plasmon resonance to characterize the binding between CHMP4 analogs mimicking the α6 motifs and Bro1 (*K*_D_ ~ 40 μM), further corroborating that both α5 and α6 motifs of CHMP4 paralogs bind to ALIX (and Bro1), and that this larger binding interface is responsible for the increased binding affinity observed here.

### Modulation of ALIX condensates by tyrosine phosphorylation and the impact of CHMP4C sequestration

**Fig. 7.**
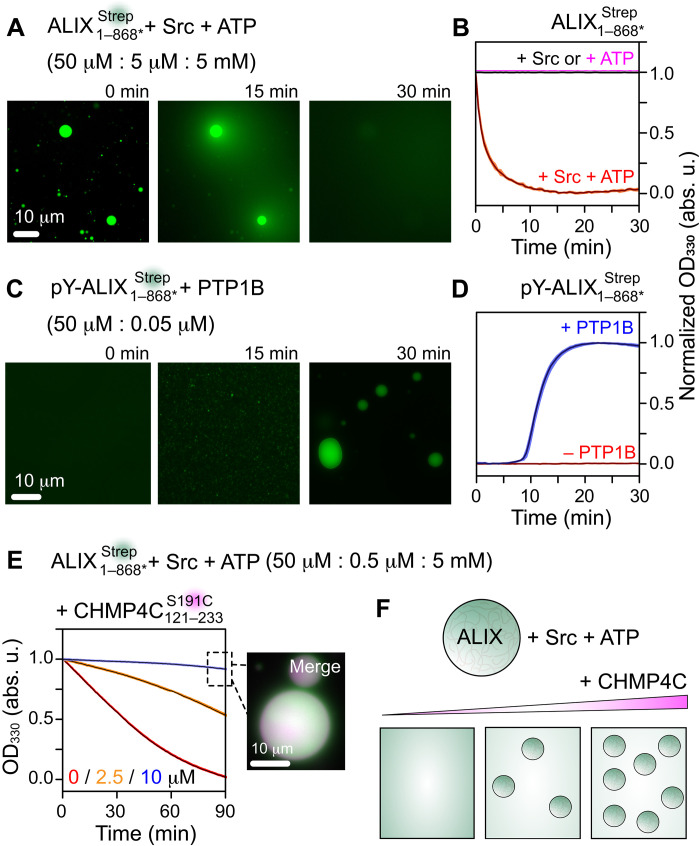
Dissolution and formation of ALIX condensates upon reversible tyrosine phosphorylation. (**A**) Time course of the dissolution of ${\mathrm{ALIX}}_{1-868*}^{\mathrm{Strep}}$ condensates upon Src-mediated phosphorylation, monitored by fluorescence microscopy. The concentrations of ${\mathrm{ALIX}}_{1-868*}^{\mathrm{Strep}}$, Src, and ATP are in parenthesis. (**B**) Dissolution kinetics of ${\mathrm{ALIX}}_{1-868*}^{\mathrm{Strep}}$ condensates monitored by turbidity assay; *n* = 3, mean (solid line), SD (shaded region). ${\mathrm{ALIX}}_{1-868*}^{\mathrm{Strep}}$ condensates were incubated with Src + ATP (red), only Src (black), and only ATP (magenta); proteins and ATP concentrations were the same as in (A). (**C**) PTP1B-mediated dephosphorylation of hyperphosphorylated ${\mathrm{ALIX}}_{1-868*}^{\mathrm{Strep}}$ (labeled as pY) resulted in the formation of ${\mathrm{ALIX}}_{1-868*}^{\mathrm{Strep}}$ condensates. Representative microscopy images at three time points are shown. (**D**) Kinetics of the formation of ${\mathrm{ALIX}}_{1-868*}^{\mathrm{Strep}}$ condensates monitored by turbidity assay. $\mathrm{pY}-{\mathrm{ALIX}}_{1-868*}^{\mathrm{Strep}}$ (50 μM) was incubated with (blue) and without (red) PTP1B (50 nM), *n* = 3. (**E**) Changes in dissolution of ${\mathrm{ALIX}}_{1-868*}^{\mathrm{Strep}}$ condensates as a function of increasing concentration of $\mathrm{CHMP}4C_{121-233}^{S191C}$, monitored by turbidity assay (*n* = 3). The concentrations of ${\mathrm{ALIX}}_{1-868*}^{\mathrm{Strep}}$, Src, and ATP are in parenthesis. The concentrations of $\mathrm{CHMP}4C_{121-233}^{S191C}$ were 0 μM (red), 2.5 μM (orange), and 10 μM (blue). Inset: A representative microscopy image of ${\mathrm{ALIX}}_{1-868*}^{\mathrm{Strep}}$ droplets with 10 μM $\mathrm{CHMP}4C_{121-233}^{S191C}$ upon 90-min incubation; see fig. S22 for images at the respective fluorescent channels, and fig. S23 for Western blot analysis of changes in Src-mediated phosphorylation of ${\mathrm{ALIX}}_{1-868*}^{\mathrm{Strep}}$ droplets with and without $\mathrm{CHMP}4C_{121-233}^{S191C}$. (**F**) Schematic of modulation of Src-mediated dissolution of ALIX condensates by CHMP4C. All experiments were carried out with 5% (w/v) PEG-4000. For fluorescence microscopy experiments, ${\mathrm{ALIX}}_{1-868*}^{\mathrm{Strep}}$ and $\mathrm{CHMP}4C_{121-233}^{S191C}$ were labeled with Alexa Fluor 488 and ATTO-647N, respectively. The concentration of Alexa Fluor 488 was 0.02 mg/ml. The concentration of ATTO-647N–labeled $\mathrm{CHMP}4C_{121-233}^{S191C}$ was 5 mol %.

Cellular ACBs are regulated by PTMs, specifically by Aurora-B–mediated (serine) phosphorylation of CHMP4C (*11*). Furthermore, ALIX-PRD fibrils dissolve and reform upon reversible tyrosine phosphorylation mediated by Src and PTP1B (*22*, *23*). Hence, we asked whether these PTMs can modulate ${\mathrm{ALIX}}_{1-868*}^{\mathrm{Strep}}$ condensates and the subsequent impact of the confinement of CHMP4 paralogs. Src-mediated hyperphosphorylation of ${\mathrm{ALIX}}_{1-868*}^{\mathrm{Strep}}$ was confirmed by Western blotting and mass spectrometry (MS; fig. S21, A and B). To explore the impact of tyrosine phosphorylation on ALIX’s phase separation, 5 μM Src and 5 mM adenosine triphosphate (ATP) were mixed with ${\mathrm{ALIX}}_{1-868*}^{\mathrm{Strep}}$ condensates (50 μM), and the resultant changes in droplet morphologies were visualized by fluorescence microscopy (Fig. 7A). After ~10 min, bursts of fluorescent light were seen emanating from larger ${\mathrm{ALIX}}_{1-868*}^{\mathrm{Strep}}$ droplets (≥5 μm in diameter), likely due to the disintegration of condensates into soluble species upon phosphorylation (movie S1). After 30 min, no droplets were visible. Similar experiments performed without Src or ATP revealed minimal alterations in droplet morphologies with time (fig. S21, C and D), thereby ruling out photobleaching as the cause of the droplet disappearance observed for samples mixed with both Src and ATP. These results were confirmed by turbidity assays (Fig. 7B). Unlike negative controls, ${\mathrm{ALIX}}_{1-868*}^{\mathrm{Strep}}$ droplets without Src or ATP that displayed no changes in turbidity, a rapid loss of turbidity was observed in ${\mathrm{ALIX}}_{1-868*}^{\mathrm{Strep}}$ droplets mixed with Src and ATP with a half-time (*t*_1/2_) of ∼5 min, confirming the breakdown of higher-order ALIX multimers into soluble species upon phosphorylation. Because condensates of ALIX constructs displayed gel-like properties (e.g., slow fusion and little FRAP recovery), we propose that Src accesses and phosphorylates the outer edges of these droplets, causing the phosphorylated species to slowly leach out, as evidenced by a gradual decrease in the droplet size with time; movie S2 was captured using total internal reflection fluorescence (TIRF) microscopy. Hyperphosphorylated ${\mathrm{ALIX}}_{1-868*}^{\mathrm{Strep}}$ (50 μM) did not phase separate with PEG-4000 (Fig. 7C). The addition of 50 nM PTP1B, however, resulted in the reformation of condensates as dephosphorylation proceeded in real time; the latter was confirmed by Western blotting (fig. S21E). The corresponding sigmoidal increase in turbidity (*t*_1/2_ ∼ 15 min) corroborated the restoration of condensates upon dephosphorylation, whereas samples without PTP1B showed no noticeable increase in turbidity (Fig. 7D). These results establish that reversible tyrosine phosphorylation modulates ALIX’s phase separation.

To assess the changes in Src-mediated dissolution of ALIX condensates comprising CHMP4 proteins, we performed microscopy experiments and turbidity assays on ${\mathrm{ALIX}}_{1-868*}^{\mathrm{Strep}}$ condensates containing increasing concentrations of $\mathrm{CHMP}4C_{121-233}^{S191C}$. These measurements were performed using 0.5 μM Src (as opposed to 5 μM Src that was used in the above experiments). Under these conditions, a gradual loss of turbidity was observed in ${\mathrm{ALIX}}_{1-868*}^{\mathrm{Strep}}$ condensates in the absence of $\mathrm{CHMP}4C_{121-233}^{S191C}$ (*t*_1/2_ ∼ 35 min; Fig. 7E). In the presence of 2.5 and 10 μM $\mathrm{CHMP}4C_{121-233}^{S191C}$, the corresponding changes in turbidity were progressively minimal. Specifically, 10 μM $\mathrm{CHMP}4C_{121-233}^{S191C}$ in ${\mathrm{ALIX}}_{1-868*}^{\mathrm{Strep}}$ condensates resulted in minimal alterations in droplet morphology and turbidity over a 90-min time course (Fig. 7E and fig. S22). Therefore, the sequestration of CHMP4C in ALIX droplets modulates their Src-mediated dissolution (Fig. 7F), perhaps because both CHMP4C and Src compete for overlapping binding sites on ALIX-PRD (see Figs. 1A and 4C). To test this, we analyzed the changes in Src-mediated phosphorylation of ${\mathrm{ALIX}}_{1-868*}^{\mathrm{Strep}}$ condensates in the absence and presence of equimolar amount of $\mathrm{CHMP}4C_{121-233}^{S191C}$ using Western blotting (fig. S23). Unlike the sample without $\mathrm{CHMP}4C_{121-233}^{S191C}$, a large decrease in band intensities of phosphorylated ${\mathrm{ALIX}}_{1-868*}^{\mathrm{Strep}}$ was observed in the presence of $\mathrm{CHMP}4C_{121-233}^{S191C}$, establishing that colocalization of CHMP4C in ALIX droplets inhibits ALIX’s phosphorylation by Src. We thus propose that the dissolution of ALIX aggregates confined within cellular ACBs can be tuned by changes in Src kinase expression and activity, further supporting the important role of Src family kinases in cytokinetic abscission.

**Fig. 8.**
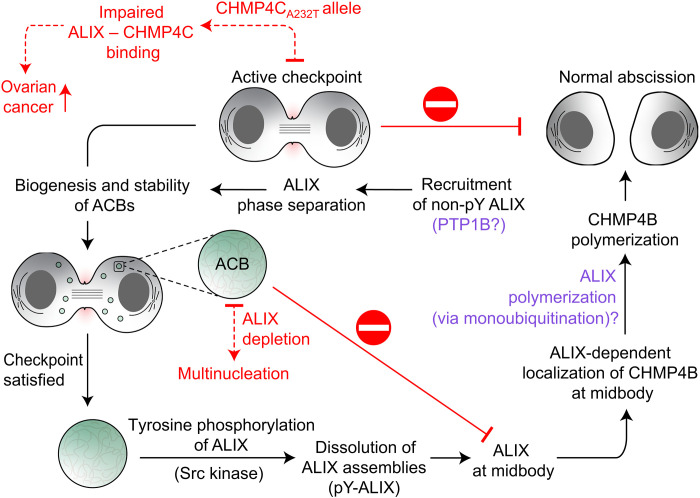
Proposed role of ALIX’s phase separation in regulation of abscission. On the basis of the findings of current study and prior results (*10*, *11*, *20*, *73*), we hypothesize that phase separation of nonphosphorylated (non-pY) ALIX will play a vital role in the biogenesis and stability of ACBs during the active abscission checkpoint (although PTP1B was used in the current study, the exact identity of the tyrosine phosphatase that dephosphorylates ALIX in vivo is not known; noted with a question mark). The sequestration of ALIX in ACBs will delay the localization of ALIX and subsequently ALIX-mediated localization of CHMP4B at the midbody. We propose that upon the resolution of the checkpoint, hyperphosphorylation (pY) of ALIX by Src kinase will dissolve ALIX assemblies into monomers. Subsequent localization of hyperphosphorylated ALIX monomers at the midbody would result in ALIX-mediated localization of CHMP4B. ALIX, like other early-acting ESCRT-I factors that assemble into a scaffolding platform (*74*), may also polymerize into higher-order assemblies (marked by a question mark), facilitating the polymerization of CHMP4B filaments and thereby CHMP4B-mediated membrane scission. We recently showed that monoubiquitination of ALIX via NEDD4-family E3 ligases, namely, WWP2 and NEDD4L, promoted its fibrilization in vitro (*75*). Note that, like many ESCRT proteins, ALIX is monoubiquitinated in vivo, which is important for its cellular functions (*76*, *77*). Red dashed arrows represent known pathological aberrations in the shown processes, including the ovarian cancer–predisposing CHMP4C mutation that overrides the checkpoint due to its defective association with ALIX (*10*) and ALIX depletion that results in multinucleated cells (*73*).

In summary, ALIX phase separates into gel-like condensates in vitro and in vivo, mediated by the amyloidogenic portion of its PRD. Recombinant CHMP4B and CHMP4C readily colocalize in ALIX condensates. Both CHMP4 paralogs used in this study are largely disordered, except for the α5 motif of the CHMP4C, which forms a stable helix. ALIX binds to α5 and α6 motifs of CHMP4 proteins, elucidating the molecular basis of ALIX-mediated CHMP4 activation as the intermolecular ALIX-CHMP4 interactions will likely relieve intramolecular autoinhibitory interactions between α5 and α1-α2 motifs in full-length CHMP4 proteins, thereby triggering their activation. ALIX condensates are modulated by PTMs, where tyrosine hyperphosphorylation and dephosphorylation of ALIX via Src and PTP1B lead to their dissolution and reformation. On the basis of these results, we argue that PTM-mediated phase separation of ALIX will contribute to the biogenesis and stability of cellular ACBs and propose a model to illustrate how ALIX’s aggregation can control abscission timing (Fig. 8). In addition, we note that the formation of phase-separated cellular compartments is primarily driven by the multivalent interactions of the sequestered biomolecules, often involving associations of their disordered regions (*13*). Eukaryotic PRDs frequently form such multivalent complexes due to their disordered nature and favorable binding properties (*22*). PRDs are also known to modulate cellular phase separation. For example, a recent report established that the PRD of microtubule-associated protein Tau drives its phase separation in vivo (*49*). Moreover, aromatic residues that are interspersed in the disordered protein regions promote phase separation through π-π stacking. In these regard, the importance of tyrosine residues was established by mutation and deletion studies (*50*–*52*) as well as by solution NMR analyses (*53*). PTMs, including tyrosine phosphorylation, are also known to regulate the formation and dissolution of biomolecular condensates. For example, phosphorylation of tyrosine residues in RNA binding proteins, namely, cell cycle–associated protein 1 (CAPRIN1) (*54*) and heterogeneous nuclear ribonucleoprotein 2 (hnRNPA2) (*55*), was shown to modulate their phase separation properties. The results presented here are in excellent agreement with these known observations. This is because the amyloidogenic portion of ALIX is enriched with proline and tyrosine residues (29 and 20%, respectively), which likely promote its phase separation via hydrophobic interactions, and that the introduction of negatively charged phosphoryl groups on tyrosine residues creates charge-charge repulsions, leading to condensate disassembly upon Src-mediated phosphorylation. Hence, the condensates of recombinant ALIX can likely be described as simple coacervates, driven by the intermolecular associations of the amyloidogenic portion of its PRD. In addition, the above observations suggest how the stability and composition of cellular condensates can be modulated by reversible tyrosine phosphorylation. While a detailed structural investigation of these intermolecular associations in vitro and their dynamic interplay with the corresponding PTMs will provide vital physical insights and is a topic of ongoing investigation in our laboratory, we note that the mechanism of the formation and dissolution of such condensates in vivo will be staggeringly more complex. Together, the results presented in this study uncover the phase separation of ALIX and its plausible role in regulating the timing of cell division.

## MATERIALS AND METHODS

### Materials

PEG-4000 was purchased from Sigma-Aldrich (catalog no. 81240). Streptavidin Alexa Fluor 488 conjugate was purchased from Thermo Fisher Scientific (catalog no. S32354). ATTO-390 maleimide, ATTO-488 *N*-hydroxysuccinimide (NHS) ester, and ATTO-647N maleimide were purchased from ATTO-TEC GmbH (catalog nos. AD 390-41, AD 488-31, and AD 647N-41, respectively) and were dissolved in dimethylformamide (DMF) at a concentration of 10 mM. ATP was purchased from Sigma-Aldrich (catalog no. A2383) and was dissolved and buffered in 100 mM tris (pH 7.5) at a concentration of 100 mM. The phospho-tyrosine mouse monoclonal antibody was purchased from Cell Signaling Technology (catalog no. 9411). The secondary antibodies goat anti-mouse immunoglobulin G and IRDye 800CW were purchased from Thermo Fisher Scientific (catalog nos. G-21040 and NC9401841, respectively). Gels for SDS–polyacrylamide gel electrophoresis (SDS-PAGE; 4 to 12% bis-tris) and Pierce bicinchoninic acid (BCA) assay kit were purchased from Thermo Fisher Scientific (catalog nos. NW04122BOX and 23227, respectively). Reagents for NMR isotopic enrichment were obtained from Cambridge Isotope Laboratories and Sigma-Aldrich. MTSL and its acetylated diamagnetic analog, (1-acetoxy-2,2,5,5-tetramethyl-δ-3-pyrroline-3-methyl) methanethiosulfonate, were purchased from Toronto Research Chemicals Inc. (catalog nos. O875000 and A167900, respectively).

### Methods

#### Recombinant protein expression and purification

Codon-optimized constructs, ${\mathrm{ALIX}}_{1-868*}^{\mathrm{Strep}}$, ALIX_1–868*_, PRD_703–868*_, ${\mathrm{PRD}}_{800-868}^{\mathrm{Strep}}$, $\mathrm{CHMP}4C_{121-233}^{S191C}$, $\mathrm{CHMP}4C_{121-233}^{G154C}$, $\mathrm{CHMP}4C_{121-233}^{M165C}$, $\mathrm{CHMP}4C_{121-233}^{S191C,\mathrm{AAA}}$, $\mathrm{CHMP}4B_{121-224}^{S184C}$, and $\mathrm{CEP}55_{160-216}^{S215C}$, were custom-synthesized from Azenta Life Sciences. The remaining constructs, ${\mathrm{PRD}}_{703-868*}^{\mathrm{Strep}}$, ${\mathrm{PRD}}_{703-800}^{\mathrm{Strep}}$, and PRD_800–868_, were reported in our previous works (*22*, *23*). ALIX-Bro1 and PTP1B were obtained from the Addgene repository, accession nos. 80641 (*3*) and 102719 (*56*), respectively. Constructs of Src kinase, ${\mathrm{MBP}}_{28-396}^{E64C}$, and tobacco etch virus (TEV) protease were gifts from A. van der Vliet (University of Vermont), G. M. Clore [National Institutes of Health (NIH)], and D. S. Waugh (NIH), respectively. See fig. S6 for the design and subcloning of each recombinant construct tested in this study.

Src kinase, PTP1B, ${\mathrm{PRD}}_{703-868*}^{\mathrm{Strep}}$, ${\mathrm{PRD}}_{703-800}^{\mathrm{Strep}}$, TEV protease, and ${\mathrm{MBP}}_{28-396}^{E64C}$ were expressed as described previously (*22*, *23*, *45*). All remaining constructs were expressed at 16°C. Cells were grown at 37°C in 1 liter of Luria-Bertani (LB) medium (MP Biomedicals, catalog no. 3002-036) at natural isotopic abundance or minimal M9 medium (*22*, *23*) for isotopic labeling. About 30 min before induction, the temperature of the cell culture was reduced to 16°C. Cells were induced with 1 mM isopropyl-β-d-thiogalactopyranoside (IPTG) at an absorbance of ∼0.8 at 600 nm and harvested after ~24 hours.

The purification schemes of ALIX-Bro1, Src kinase, PTP1B, and TEV protease are described previously (*22*, *23*, *45*). For ${\mathrm{ALIX}}_{1-868*}^{\mathrm{Strep}}$, cells were resuspended in a lysis buffer containing 50 mM tris (pH 8.0), 250 mM NaCl, 1 mM EDTA, and 1 mM dithiothreitol (DTT). Cells were lysed using EmulsiFlex-C3 (Avestin) before being cleared by centrifugation (48,380*g*, 30 min). The resultant supernatant was loaded onto a Strep-Tactin Sepharose column (Cytiva), preequilibrated with lysis buffer, and eluted in the same buffer containing 2.5 mM d-desthiobiotin. Eluted protein was diluted with a running buffer containing 50 mM tris (pH 8.0) and 5 mM β-mercaptoethanol (BME) and loaded onto a Q-Sepharose HP column (Cytiva) with a 0 to 1 M NaCl gradient in the same running buffer. Eluted protein was concentrated (Amicon Ultra-15, 30-kDa cutoff, EMD Millipore) and loaded onto a HiLoad 26/600 Superdex 200 pg column (Cytiva) preequilibrated with 50 mM tris (pH 8.0), 50 mM NaCl, 1 mM EDTA, and 1 mM DTT. Relevant fractions were pooled, flash-frozen, and stored at −80°C. For the corresponding construct without the strep tag, ALIX_1–868*_, a similar protocol was used. The cell lysate was initially loaded onto a HisTrap column (Cytiva) preequilibrated with the lysis buffer mentioned above (sans DTT) and eluted with a 0 to 1 M imidazole gradient. Eluted protein was further purified using a Q-Sepharose HP column as described above. Relevant fractions were mixed with TEV protease (molar ratio of 5:1) to cleave off the N-terminal B1 domain of protein G (GB1) fusion tag (*57*). Proteolysis was performed at room temperature (~12 hours) and assessed for completion using SDS-PAGE. The reaction mixture was loaded back onto a HisTrap column. The flow-through fractions of the hydrolyzed product were pooled, concentrated, further purified using the abovementioned sizing column, and subsequently stored at −80°C.

A purification scheme like the one used for ALIX_1–868*_ was used for $\mathrm{CEP}55_{160-216}^{S215C}$ and the CHMP4 constructs. Proteins were initially purified by nickel affinity chromatography (HisTrap column, Cytiva) followed by size exclusion chromatography (HiLoad 26/600 Superdex 75-pg column, Cytiva). The resultant fractions were mixed with TEV protease to cleave the N-terminal GB1 fusion tag. After completion of proteolysis, the reaction mixture was purified using nickel affinity and size exclusion chromatography.

ALIX-PRD constructs, ${\mathrm{PRD}}_{703-868*}^{\mathrm{Strep}}$, ${\mathrm{PRD}}_{703-800}^{\mathrm{Strep}}$, and PRD_800–868_, were purified by a combination of affinity chromatography and reversed-phase high-performance liquid chromatography (HPLC), as described previously (*22*, *23*). A protocol similar to the one used for ${\mathrm{PRD}}_{703-868*}^{\mathrm{Strep}}$ was used for purification of ${\mathrm{PRD}}_{800-868}^{\mathrm{Strep}}$. Cells resuspended in 50 mM tris (pH 8.0) and 6 M guanidine hydrochloride (GdmCl) were lysed by heat shock (80°C for 5 min, followed by 10 min on ice) and cleared by centrifugation. The cell lysate was filtered through a 0.45-μm vacuum-driven filtration device (Stericup, Sigma-Aldrich) and loaded onto a HisTrap column, preequilibrated with lysis buffer. The bound protein was washed with 10 column volumes of refolding buffer containing 50 mM tris (pH 8.0) and 250 mM NaCl and eluted in the same buffer using a 0 to 1 M imidazole gradient. Eluted fractions were pooled and mixed with recombinant TEV protease (molar ratio 50:1) to cleave off the N-terminal GB1 fusion tag. Upon completion of proteolysis (assessed using SDS-PAGE gel), the precipitated product was solubilized in 3 M GdmCl and 5% (v/v) dimethyl sulfoxide (DMSO) and further purified using reversed-phase HPLC (Jupiter 10 μm C18 300 Å column) using a 25 to 37% acetonitrile gradient comprising 0.1% trifluoroacetic acid. Pooled eluted fractions were lyophilized and stored at −80°C.

Protein concentrations were measured using UV absorbance at a 280-nm wavelength. Note that because of the lack of tyrosine and tryptophan residues, the concentrations of $\mathrm{CHMP}4C_{121-233}^{S191C,\mathrm{AAA}}$ were measured using the colorimetric BCA assay (*58*). All protein constructs were verified by MS as described before (*22*, *23*, *45*).

#### Sedimentation velocity AUC

Sedimentation velocity experiments on ${\mathrm{ALIX}}_{1-868*}^{\mathrm{Strep}}$ and CHMP4 paralogs were carried out at 50,000 rpm and 30°C on a Beckman Coulter ProteomeLab XL-I analytical ultracentrifuge and An50-Ti rotor following standard protocols (*59*). Stock solutions of proteins, dialyzed into a buffer containing 20 mM sodium phosphate (pH 6.5), 1 mM tris(2-carboxyethyl)phosphine (TCEP), and 2 mM EDTA, were diluted to ~40 μM and loaded into 12-mm two-channel centerpiece cells. Sedimentation data were collected using optical detection systems for absorbance (280 nm) and interference (655 nm) and analyzed using our published protocols (*22*, *60*–*62*).

#### Fluorophore labeling

$\mathrm{CHMP}4C_{121-233}^{S191C}$, $\mathrm{CHMP}4B_{121-224}^{S184C}$, and ${\mathrm{MBP}}_{28-396}^{E64C}$ were mixed with a four-molar equivalent of ATTO-647N maleimide in 20 mM Hepes (pH 7.5), 50 mM NaCl, and 1 mM EDTA. The reaction was allowed to proceed for ~30 min before being quenched by the addition of 50-molar equivalent of BME. Unreacted dye was removed using a PD MidiTrap G-25 desalting column (Cytiva). A similar procedure was used to conjugate ATTO-390 to $\mathrm{CHMP}4B_{121-224}^{S184C}$. For TIRF microscopy, ${\mathrm{PRD}}_{703-868*}^{\mathrm{Strep}}$ was conjugated to ATTO-488 NHS ester. ${\mathrm{PRD}}_{703-868*}^{\mathrm{Strep}}$ was mixed with a four-molar equivalent of ATTO-488 NHS ester in 50 mM Hepes (pH 7.5) and 20% (v/v) DMSO. The reaction was allowed to proceed for ~30 min, followed by the addition of 6 M GdmCl. The unreacted dye was removed by dialyzing the reaction mixture in 20 mM 3-(cyclohexylamino)-1-propanesulfonic acid (CAPS) (pH 10) and 50 mM NaCl (Slide-A-Lyzer G2 dialysis cassettes, Thermo Fisher Scientific). The fluorophore labeling efficiencies of $\mathrm{CHMP}4C_{121-233}^{S191C}$, $\mathrm{CHMP}4B_{121-224}^{S184C}$, and ${\mathrm{PRD}}_{703-868*}^{\mathrm{Strep}}$ were determined by UV-visible (UV-Vis) absorbance (~95% efficiency in all samples). For Alexa Fluor 488 labeling, streptavidin Alexa Fluor 488 conjugate (0.2 mg/ml) was added to ALIX constructs carrying a C-terminal strep tag (the concentrations of ALIX constructs were 50 μM each). To monitor Src-mediated dissolution of ${\mathrm{ALIX}}_{1-868*}^{\mathrm{Strep}}$ condensates, the concentration of Alexa Fluor 488 conjugate was lowered to 0.02 mg/ml.

#### Phase separation of ALIX constructs

ALIX constructs (${\mathrm{ALIX}}_{1-868*}^{\mathrm{Strep}}$ and ALIX_1–868*_) were dialyzed in 20 mM Hepes (pH 7.5), 50 mM NaCl, 1 mM DTT, and 1 mM EDTA. Phase separation was initiated by the addition of 5% (w/v) PEG-4000. In all samples, the protein concentration was maintained at 50 μM. For ALIX-PRD constructs, ${\mathrm{PRD}}_{703-868*}^{\mathrm{Strep}}$, ${\mathrm{PRD}}_{703-800}^{\mathrm{Strep}}$, and ${\mathrm{PRD}}_{800-868}^{\mathrm{Strep}}$, lyophilized samples were reconstituted in 20 mM CAPS (pH 10) and 50 mM NaCl and concentrated to ~2 mM stock solutions. Phase separation was induced by a rapid dilution of corresponding samples in the abovementioned Hepes buffer comprising 5% (w/v) PEG-4000 to a final protein concentration of 50 μM.

#### Microscopy

Condensate samples were applied to microscope slides (Thermo Fisher Scientific, catalog no. 12-550-003) and sandwiched between coverslips (VWR, catalog no. 48366-227). Slides were incubated for 5 min at room temperature before imaging to allow condensates to adhere to the glass surface. Differential interference contrast (DIC) microscopy was performed on a Nikon Ti2 widefield microscope equipped with a DS-Qi2 complementary metal-oxide semiconductor (CMOS) camera and a 100×/1.49 numerical aperture (NA) oil DIC N2 objective. The sample was excited by a 395/470/640-nm laser controlled by Lumencor SpectraX for imaging of ATTO-390, Alexa Fluor 488, and ATTO-647N, respectively.

To determine the particle size, the condensates of ${\mathrm{ALIX}}_{1-868*}^{\mathrm{Strep}}$, ${\mathrm{PRD}}_{703-868*}^{\mathrm{Strep}}$, and ${\mathrm{PRD}}_{800-868}^{\mathrm{Strep}}$ were imaged in multiple 3 × 3 tile sets, where each tile spanned an area of 100 × 100 μm^2^. Particles of an area ≥ 0.08 μm^2^ (with fluorescence above background) were identified using the analyze particles function in Fiji/ImageJ (*63*), and their diameters were determined assuming circular morphologies. For the corresponding constructs without the strep tag, particles were identified using condensate edges. To quantify ThT colocalization within the condensates, condensates of ${\mathrm{ALIX}}_{1-868*}^{\mathrm{Strep}}$, ${\mathrm{PRD}}_{703-868*}^{\mathrm{Strep}}$, and ${\mathrm{PRD}}_{800-868}^{\mathrm{Strep}}$ were prepared with 20 μM ThT and imaged immediately using a 470-nm laser. Images for each construct were collected under identical laser power, gain, and exposure time to facilitate a quantitative comparison of ThT fluorescence.

In vitro FRAP experiments were performed on a Nikon point scanning confocal C2 with 2 GaAsP photomultiplier tubes using a Plan Apo λ 60×/1.4 NA oil objective. Data collection consisted of six pre-photobleaching frames excited at 0.5% 488-nm laser power, followed by photobleaching with two iterations of 50% 488-nm laser power directed at the bleaching area for 10 s, and subsequently excited at 0.2% 488-nm laser power at 0.5-s intervals for 120 frames as post-photobleaching frames. Images were corrected for background fluorescence, and intensity from the bleached region was normalized against an unbleached region on a nearby condensate of similar size.

Time-lapse images of ${\mathrm{ALIX}}_{1-868*}^{\mathrm{Strep}}$ + Src + ATP and pY-${\mathrm{ALIX}}_{1-868*}^{\mathrm{Strep}}$ + PTP1B reactions were acquired using an EVOS-M5000 imaging system (Thermo Fisher Scientific) equipped with a PlanApo N 60× oil objective and green fluorescent protein (GFP) and TagBFP light-emitting diode (LED) cubes. All the above microscopy images were collected at ambient temperature and analyzed with Fiji/ImageJ (*63*).

TIRF microscopy for time-lapse imaging of Src-mediated dissolution of ${\mathrm{PRD}}_{703-868*}^{\mathrm{Strep}}$ condensates was performed on a Nikon Eclipse Ti2-E equipped with an iXon Ultra 897 electron multiplying charge-coupled device camera (Andor) and Apo TIRF 60×/1.49 NA oil DIC N2 objective. The TIRF angle was controlled by the N-STORM illumination arm in the Nikon Elements software. The sample was excited by a 488-nm laser (10% laser power) controlled by an Agilent laser box. The excitation light is reflected by a quad dichroic, and the emission is filtered by a quad emission filter (emission window, 502 to 549 nm). Images were acquired at 15-s intervals for 1.5 hours. The spinning disk pinhole was 50 μm.

#### Living cells

Plasmid of mNeonGreen in pcDNA3.1 was generated by polymerase chain reaction (PCR) amplification from pmNeonGreen-N1 (Allele Biotechnology) using appropriate primers (table S3). The Bam HI/Eco RI–digested PCR product was then ligated into Bam HI/Eco RI–digested pcDNA3.1 backbone. Plasmids of ${\mathrm{ALIX}}_{1-868*}^{\mathrm{mNG}}$ and ${\mathrm{ALIX}}_{1-702}^{\mathrm{mNG}}$ were generated from ${\mathrm{ALIX}}_{1-868*}^{\mathrm{Strep}}$ plasmid by amplifying the respective segments using PCR and appropriate primers (table S3). The resultant products were inserted into pcDNA3.1-mNG vector using Gibson assembly protocol and HiFi DNA assembly master mix (New England Biolabs, catalog no. E2621S). Plasmid of ${\mathrm{ALIX}}_{1-868}^{\mathrm{mNG}}$ was generated from ${\mathrm{ALIX}}_{1-868*}^{\mathrm{mNG}}$ using PCR and appropriate primers containing the point mutation primers (table S3). All three ALIX constructs were verified by Sanger sequencing (Azenta Life Sciences).

HEK293T cells (American Type Culture Collection, catalog no. CRL-3216) were cultured in Dulbecco’s modified Eagle’s medium (Thermo Fisher Scientific, catalog no. 11-885-084) containing glucose (4.5 g/liter), 10% fetal bovine serum (Thermo Fisher Scientific, catalog no. 26-140-079), and 1% (v/v) penicillin-streptomycin (Thermo Fisher Scientific, catalog no. 15-140-122). Cells were grown in humidified incubators (Heracell150) at 37°C and with 5% carbon dioxide. Cells were checked for mycoplasma using Hoechst staining (Thermo Fisher Scientific, catalog no. 62249). For microscopy imaging, cells were plated on 35-mm glass-bottomed dishes (Cellvis, catalog no. D35-14-1.5-N). Cells were transfected 2 to 24 hours with 500 ng of plasmid after plating using 1.5 μl of PolyJet in vitro DNA transfection reagent (SignaGen Laboratories, catalog no. SL100688) and imaged ~24 hours after transfection. Cell density was 50 to 80% confluent on the day of transfection.

For all live-cell imaging experiments, cells were washed and incubated in Hanks’ balanced salt solution (Thermo Fisher Scientific, catalog no. 14065056) buffered with 20 mM Hepes (pH 7.4) and supplemented with glucose (2 g/liter). Images were acquired on a Zeiss AxioObserver Z7 microscope equipped with a 40 × 1.4 NA oil immersion objective, Prime 95B sCMOS camera (Photometrics) controlled by MetaFluor fluorescence ratio imaging software (Molecular Devices LLC). Imaging was performed using an ET480/30× excitation filter with a T505dcxr dichroic mirror, and an ET535/50× emission filter. All filters were alternated by a Lambda 10-2 filter-changer (Sutter Instruments). Exposure times ranged between 50 and 500 ms. Raw fluorescence images were corrected by subtracting the background fluorescence intensity of a cell-free region from the emission intensities of ALIX-expressing cells.

In-cell FRAP experiments were performed on a Nikon Ti2 C2 confocal microscope equipped with a CSU-X1 spinning disc (Yokogawa), a 100 × 1.49 NA oil objective (Nikon), and 405/488/561/640-nm laser lines, dual Prime 95B sCMOS camera (Teledyne photometrics), Okolabs stage-top incubator, and OptiMicroscan FRAP box (Mad City Labs Inc.). The selected condensates were bleached for 500 ms with 405-nm laser (100% power) and observed using 488-nm laser and a single band-pass filter (525/36 nm) every 1 s for 3 min after bleaching. Intensity data were collected using NIS-Elements and normalized.

#### CD spectroscopy

CD measurements (178 to 280 nm, 1-nm data pitch, continuous scanning with 1-nm bandwidth, 60 nm/min, and 5 accumulations) with ~10 μM CHMP4 paralogs in 10 mM sodium phosphate (pH 6.5) were carried out in 1-mm quartz cuvettes (Thermo Fisher Scientific, catalog no. NC9651589) using an Aviv model 215 spectrometer. CD data were analyzed as described previously (*23*).

#### Nuclear magnetic resonance

Samples of ^15^N- or ^15^N/^13^C-labeled or ^15^N/^2^H- or ^15^N/^2^H/^13^C-labeled CHMP4 constructs, ^15^N/^13^C-labeled ${\mathrm{PRD}}_{703-800}^{\mathrm{Strep}}$, and ^15^N/^2^H- or ^15^N/^13^C/^2^H-labeled Bro1 were prepared in a buffer comprising 20 mM sodium phosphate (pH 6.5), 1 mM TCEP, and 2 mM EDTA. An identical buffer (sans TCEP) was used for intramolecular PRE experiments. All NMR samples contained 7% (v/v) deuterium oxide (D_2_O).

NMR experiments were carried out at 30°C on Bruker 600- and 800-MHz spectrometers equipped with z-gradient triple resonance cryoprobes. Spectra were processed using NMRPipe (*64*) and analyzed using the CCPN software suite (*65*). Sequential ^1^H, ^15^N, and ^13^C backbone resonance assignments of CHMP4 constructs, in the absence and presence of Bro1/${\mathrm{PRD}}_{703-800}^{\mathrm{Strep}}$, were carried out using TROSY-based three-dimensional (3D) triple resonance experiments (*66*). ^3^*J*_HN-Hα_ couplings were measured on uniformly ^15^N-labeled CHMP4 paralogs (0.5 mM each) using a WATERGATE-optimized 2D TROSY pulse sequence (*67*). ^15^N-*R*_1_ and *R*_1ρ_ measurements (*68*) were carried out on uniformly ^15^N-labeled CHMP4 paralogs (0.5 mM each) at 800 MHz using our previously described protocol (*45*). Intramolecular PREs were measured on two CHMP4C constructs ($\mathrm{CHMP}4C_{121-233}^{G154C}$ and $\mathrm{CHMP}4C_{121-233}^{M165C}$) from the peak height ratios between two 2D ^1^H-^15^N TROSY-HSQC spectra with paramagnetic (MTSL-labeled) and diamagnetic (labeled with acetylated diamagnetic analog of MTSL) samples, 0.2 mM each. The procedure for site-specific spin labeling is described in our previous work (*23*). Lifetime line-broadening (Δ*R*_2_) values of CHMP4 constructs are given by the difference in ^15^N-*R*_2_ values obtained in the presence and absence of ALIX domains, Bro1 and ${\mathrm{PRD}}_{703-800}^{\mathrm{Strep}}$. NMR chemical shift perturbation experiments were performed using 0.15 mM ^15^N-labeled CHMP4 constructs and unlabeled Bro1 and ${\mathrm{PRD}}_{703-800}^{\mathrm{Strep}}$ (0.075 to 0.45 mM). Similar experiments were carried out using 30 μM ^15^N/^2^H-labeled CHMP4 constructs, namely, $\mathrm{CHMP}4C_{121-233}^{S191C}$, $\mathrm{CHMP}4C_{121-233}^{S191C,\mathrm{AAA}}$, and $\mathrm{CHMP}4B_{121-224}^{S184C}$, and 90 μM unlabeled ${\mathrm{ALIX}}_{1-868*}^{\mathrm{Strep}}$. Perturbations were calculated as follows: Δ_H/N_ = [(Δδ_HN_)^2^ + (0.154 × Δδ_N_)^2^]^1/2^, where Δδ_HN_ and Δδ_N_ are the ^1^H_N_ and ^15^N chemical shift differences in parts per million, respectively, between free and bound states. In addition, chemical shift perturbation experiments were performed using 0.1 mM ^15^N/^2^H-labeled Bro1 domain and 0.3 mM unlabeled CHMP4 constructs. The backbone resonance assignments for Bro1 were taken from our previously published work (*23*) and further confirmed using TROSY-based 3D HNCA and HNCO experiments performed on samples comprising 0.5 mM ^15^N/^2^H/^13^C-labeled Bro1 and 1 mM CHMP4 paralogs, $\mathrm{CHMP}4C_{121-233}^{S191C}$ and $\mathrm{CHMP}4B_{121-224}^{S184C}$. Similar experiments were carried out using 0.15 mM ^15^N/^13^C-labeled ${\mathrm{PRD}}_{703-800}^{\mathrm{Strep}}$and 0.45 mM unlabeled CHMP4 constructs. Because of its high proline content (108 amino acids, ~28% proline residues), identical measurements were performed using 2D ^13^C-^15^N CON correlation experiments (*69*). The backbone resonance assignments for ${\mathrm{PRD}}_{703-800}^{\mathrm{Strep}}$were obtained from our published work (*22*). Δ_H/N_ perturbations were calculated using the above-described formula. Δ_C′/N_ perturbations were calculated using Δ_C′/N_ = [(0.3 × Δδ_C′_)^2^ + (0.154 × Δδ_N_)^2^]^1/2^, where Δδ_C′_ and Δδ_N_ are the ^13^C′ and ^15^N chemical shift differences in parts per million, respectively, between free and bound states.

#### Isothermal titration calorimetry

ITC measurements were performed using a low-volume Affinity ITC calorimeter (TA Instruments). Aliquots (1.6 to 2.6 μl) of solutions containing between 300 and 500 μM CHMP4 paralogs, $\mathrm{CHMP}4C_{121-233}^{S191C}$ and $\mathrm{CHMP}4B_{121-224}^{S184C}$, were individually injected (20 injections) into a cell containing 30 to 50 μM ${\mathrm{ALIX}}_{1-868*}^{\mathrm{Strep}}$ or Bro1. The experiments were performed at 25°C in the same buffer that was used for NMR measurements. Additional measurements of interactions between $\mathrm{CHMP}4C_{121-233}^{S191C}$ or $\mathrm{CHMP}4C_{121-233}^{S191C,\mathrm{AAA}}$ and ${\mathrm{ALIX}}_{1-868*}^{\mathrm{Strep}}$ were carried out in the presence of 150 mM NaCl (the remaining buffer composition was the same as above). Note that for $\mathrm{CHMP}4C_{121-233}^{S191C,\mathrm{AAA}}$-${\mathrm{ALIX}}_{1-868*}^{\mathrm{Strep}}$ interactions, the protein concentrations were 1500 μM $\mathrm{CHMP}4C_{121-233}^{S191C,\mathrm{AAA}}$ and 300 μM ${\mathrm{ALIX}}_{1-868*}^{\mathrm{Strep}}$. Results were analyzed using NanoAnalyze software (TA Instruments).

#### Transmission electron microscopy

The solution comprising condensates of 50 μM ${\mathrm{PRD}}_{703-868*}^{\mathrm{Strep}}$ was incubated at room temperature for 2 days. TEM sample was prepared using our published protocol (*70*). TEM images were acquired using a JEM-1400Plus transmission electron microscope (JEOL) and recorded on a OneView digital camera (Gatan).

#### Reversible tyrosine phosphorylation of ALIX

To produce hyperphosphorylated (pY) ${\mathrm{ALIX}}_{1-868*}^{\mathrm{Strep}}$, 50 μM ${\mathrm{ALIX}}_{1-868*}^{\mathrm{Strep}}$, 5 μM Src, and 5 mM ATP were mixed in a buffer containing 50 mM tris (pH 7.5), 50 mM NaCl, 5 mM MgCl_2_, and 2 mM DTT. The reaction was allowed to proceed for ∼18 hours at 30°C. Src was removed from the reaction mixture using a HisTrap column (Cytiva) preequilibrated with 50 mM tris (pH 8) and 50 mM NaCl. The resultant flow-through fractions of pY-${\mathrm{ALIX}}_{1-868*}^{\mathrm{Strep}}$were pooled, and excess ATP and adenosine diphosphate (ADP) were removed using a HiPrep 26/10 Desalting column (Cytiva) preequilibrated with 20 mM Hepes (pH 7.5), 50 mM NaCl, 1 mM DTT, and 1 mM EDTA.

The reaction mixtures comprising ${\mathrm{ALIX}}_{1-868*}^{\mathrm{Strep}}$ + Src + ATP and pY-${\mathrm{ALIX}}_{1-868*}^{\mathrm{Strep}}$ + PTP1B (and corresponding control samples) were loaded into 1-mm quartz cuvettes (Starna Cells Inc.), and turbidity measurements were recorded at an optical density at 330 nm every 30 s using an Agilent Cary 50 Bio UV-Vis spectrophotometer. Reversible tyrosine phosphorylation of ${\mathrm{ALIX}}_{1-868*}^{\mathrm{Strep}}$ was also assessed using Western blotting using our published protocol (*22*, *23*).

## Supplementary Materials

### This PDF file includes:

Figs. S1 to S23 Tables S1 to S3 Legends for movies S1 and S2 References

## Notes

View/request a protocol for this paper from *Bio-protocol*.
