# Supplementary material for: Reversible phase separation of ESCRT protein ALIX through tyrosine phosphorylation
Source: Sci Adv. 2023 Jul 14;9(28):eadg3913. doi: 10.1126/sciadv.adg3913 (PMC10348681; doi:10.1126/sciadv.adg3913)
Supplement: Supplementary file 1 — Figs. S1 to S23 Tables S1 to S3 Legends for movies S1 and S2 References [file sciadv.adg3913_sm.pdf]

Science Advances
AAAS

Supplementary Materials for

Reversible phase separation of ESCRT protein ALIX through tyrosine phosphorylation

Ruben D. Elias *et al.*

Corresponding author: Lalit Deshmukh, ldeshmukh@ucsd.edu

Sci. Adv. **9**, eadg3913 (2023)
DOI: 10.1126/sciadv.adg3913

The PDF file includes:

Figs. S1 to S23
Tables S1 to S3
Legends for movies S1 and S2
References

Other Supplementary Material for this manuscript includes the following:

Movies S1 and S2

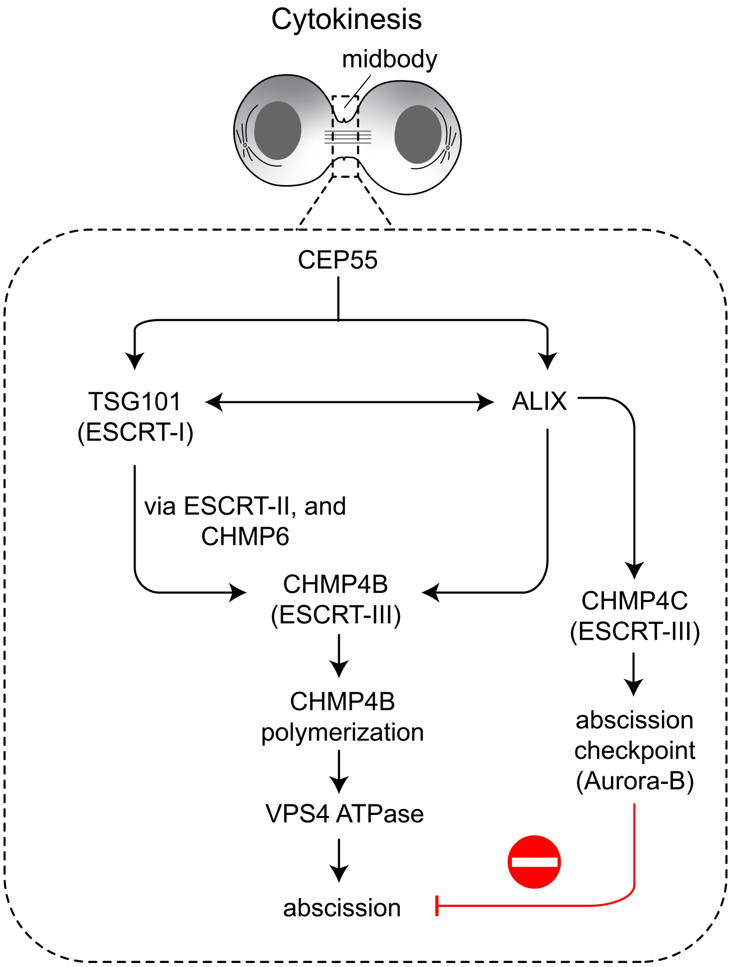

Figure S1. The ESCRT machinery in cytokinetic abscission. Schematic of the recruitment pathways of ESCRT-factors to the midbody, derived from prior biochemical, cellular, and functional results (*6, 7*). ALIX and TSG101 are recruited to the midbody by CEP55. However, abscission can also proceed via CEP55-independent pathways (*78, 79*); not shown.

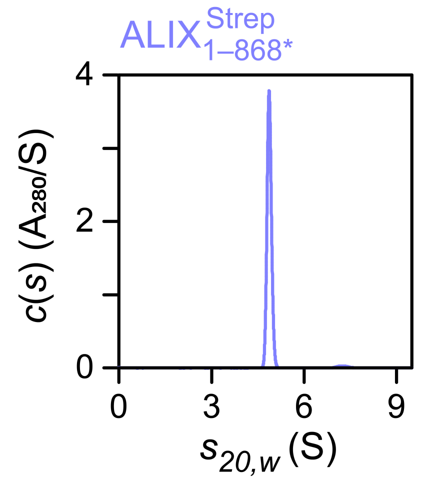

Figure S2. Recombinant ALIX is monomeric in solution. Absorbance sedimentation c(s) profile of $ALIX^{Strep}_{1–868*}$ with a single species at 5.92 S and ~88 kDa (calculated monomer mass: 96.891 kDa; cf. Fig. S6B), establishing the presence of a monodispersed monomer. The protein concentration was ~40 µM. AUC measurements were carried out at 30 ºC in 20 mM sodium phosphate, pH 6.5, 1 mM TCEP, and 2 mM EDTA.

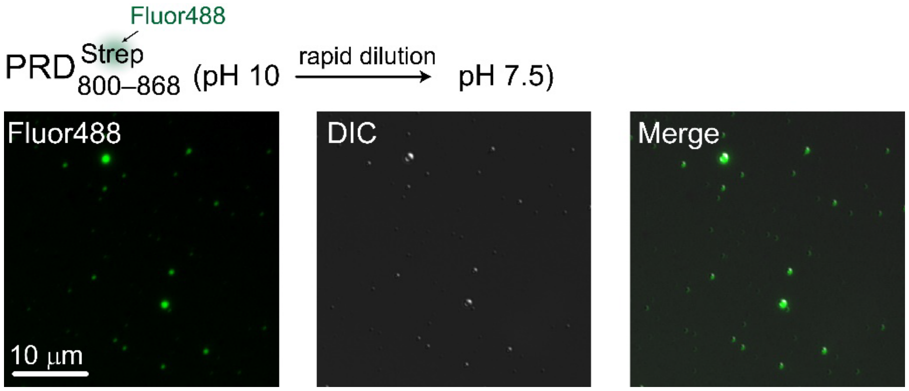

Figure S3. Phase separation of $PRD^{Strep}_{800–868}$ in the absence of PEG-4000. Representative microscopy images of Alexa-Fluor488-labeled $PRD^{Strep}_{800–868}$ droplets; DIC = differential interference contrast. The concentrations of $PRD^{Strep}_{800–868}$ and Streptavidin Alexa-Fluor488 were 50 μM and 0.2 mg/mL, respectively.

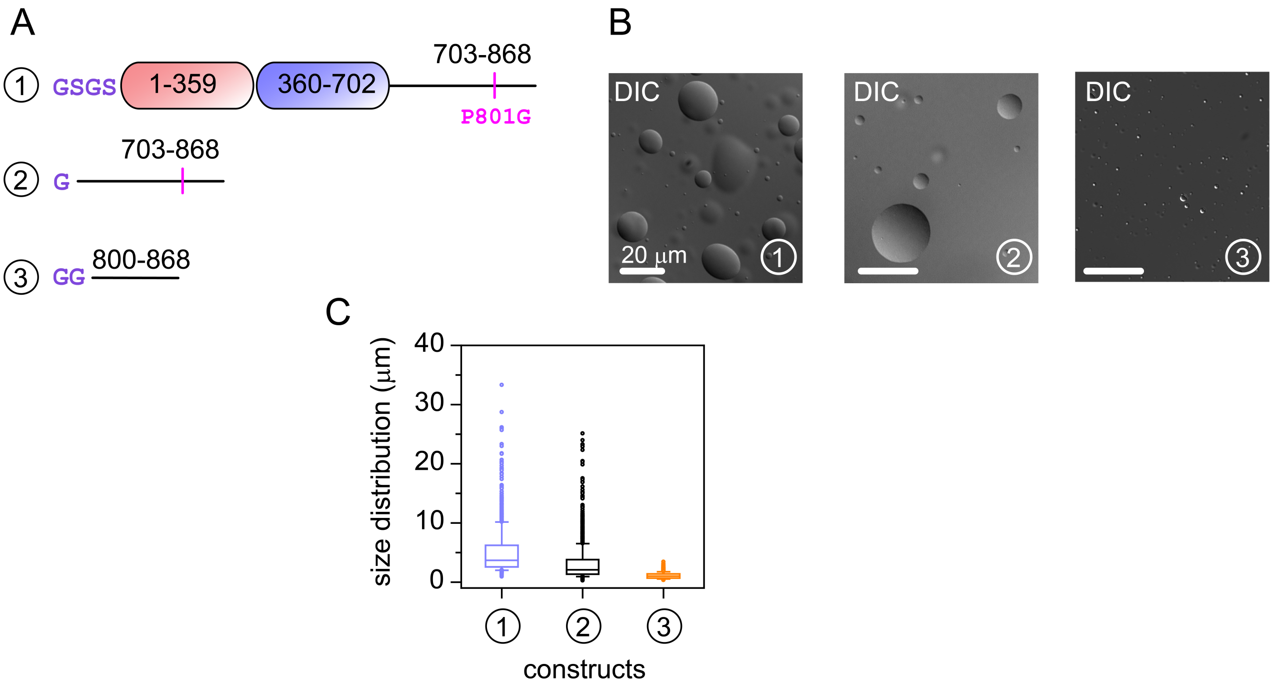

Figure S4. Phase separation of ALIX constructs in the absence of strep tags. (**A**) List of ALIX constructs used to rule out the contribution of the strep tag in its phase separation, namely $ALIX_{1–868*}$, $PRD_{703–868*}$, and $PRD_{800–868}$ (constructs 1, 2, and 3, respectively). Remnant non-native residues of the TEV protease cleavage sites are labeled in purple. The location of P801G mutation is marked with pink vertical line. (**B**) DIC images of droplets made by each ALIX constructs. (**C**) Box plot of the size distribution of condensates made by each construct, n ≥ 950. Median diameters of the condensates were as follows: ~4 μm ($ALIX_{1–868*}$), ~2 μm ($PRD_{703–868*}$), and 0.5 μm ($PRD_{800–868}$). All experiments were performed at room temperature in 20 mM HEPES, pH 7.5, 50 mM NaCl, 1 mM DTT, 1 mM EDTA, and 5% (w/v) PEG-4000, with 50 μM proteins. For ALIX-PRD constructs, lyophilized polypeptides were dissolved in a buffer containing 20 mM CAPS, pH 10, and 50 mM NaCl, followed by a rapid dilution in the above-mentioned buffer.

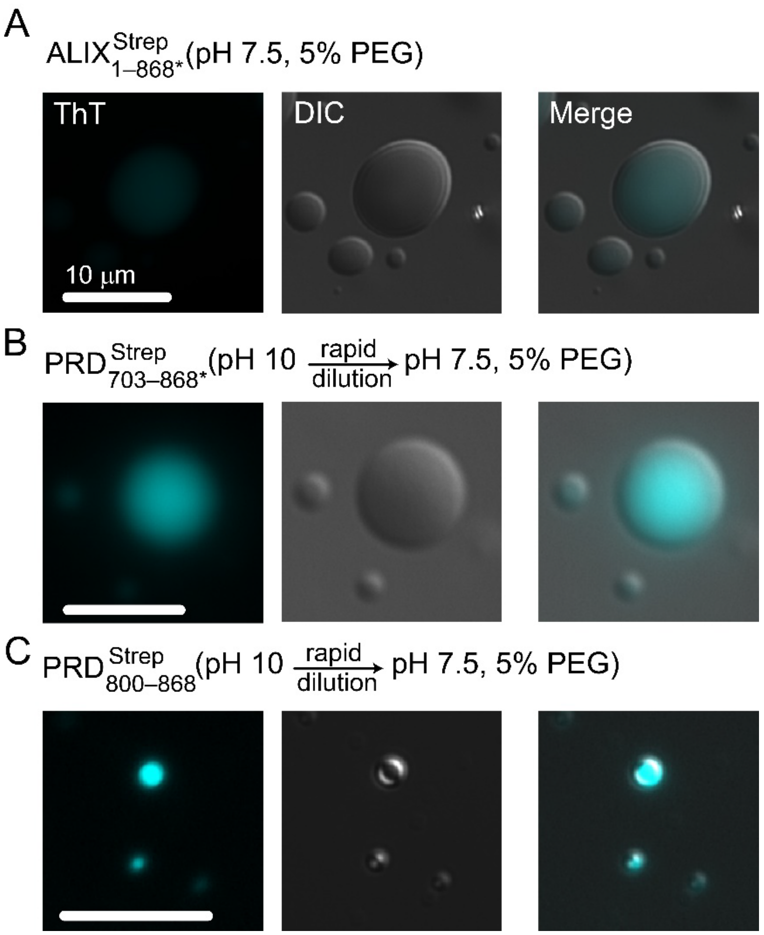

Figure S5. Differential co-partitioning of Thioflavin T (ThT) in ALIX condensates. Representative microscopy images depicting a varying degree of co-partitioning of the amyloid-sensitive dye, ThT, in condensates made by (**A**) ALIX$^{Strep}_{1–868*}$, (**B**) PRD$^{Strep}_{703–868*}$, and (**C**) PRD$^{Strep}_{800–868}$. Experimental conditions and protein concentrations were the same as those described in Fig. S4 caption. The concentration of ThT was 20 μM. Images were taken immediately after the formation of condensates.

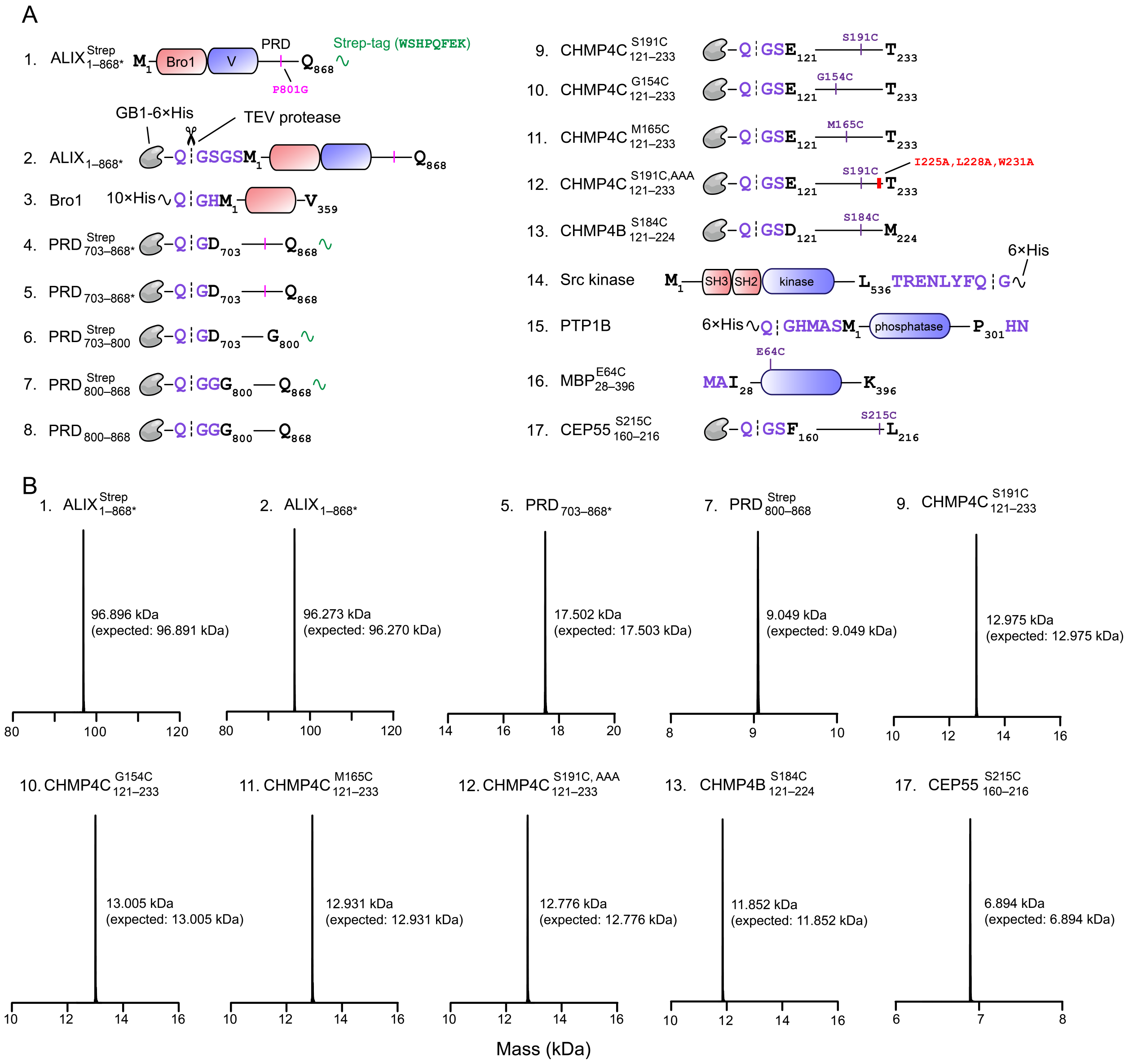

Figure S6. Constructs used in current study. (**A**) List of constructs used in current study. Constructs 1, 2, 4–12, and 16 were custom-synthesized from Azenta Life Sciences. Note that GB1-6xHis denotes B1 domain of protein G, GB1 (*57*), used to enhance protein expression levels, followed by a spacer sequence, a polyhistidine (6xHis) affinity tag, and a TEV protease cleavage site. Constructs 1–12 and 16 were subcloned in pET11a and expressed in BL21(DE3) competent cells (Agilent). Constructs 3 and 14 were obtained from the Addgene repository [accession no. 80641 (*3*) and 102719 (*56*), respectively]. Constructs 1, 2, 5, 7, 9–13, and 17 were deposited in the Addgene repository as a part of the current study (accession no. 180024, 180025, 180029, 180023,

180027, 190783, 190784, 199242, 180026, and 186793 respectively). Constructs 4, 6, and 8 were deposited in the Addgene repository as a part of our published works (*22, 23*) [accession no. 164444, 141344, and 141345, respectively]. Constructs 14 and 16 were generous gifts from Albert van der Vliet (University of Vermont), and G. Marius Clore (NIH). Src kinase (construct 14) was expressed in BL21-AI cells (Thermo Fisher Scientific). (**B**) Analysis of recombinant proteins using liquid chromatography–electrospray ionization–time-of-flight mass spectrometry (LC–ESI–TOFMS). Masses of constructs 3, 4, 6, 8, and 14–16 were reported in our previously published works (*22, 23*).

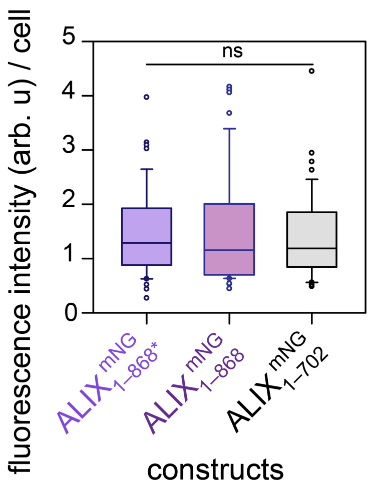

Figure S7. Expression levels of three ALIX constructs in HEK293T cells determined by fluorescence microscopy. To quantify fluorescence intensities stemming from the expression of ALIX constructs, namely $ALIX^{mNG}_{1–868*}$, $ALIX^{mNG}_{1–868}$, and $ALIX^{mNG}_{1–702}$ (see Fig. 2A, main text, for construct design), the background fluorescence intensity of a cell-free region was subtracted from the emission intensities of ALIX expressing cells.

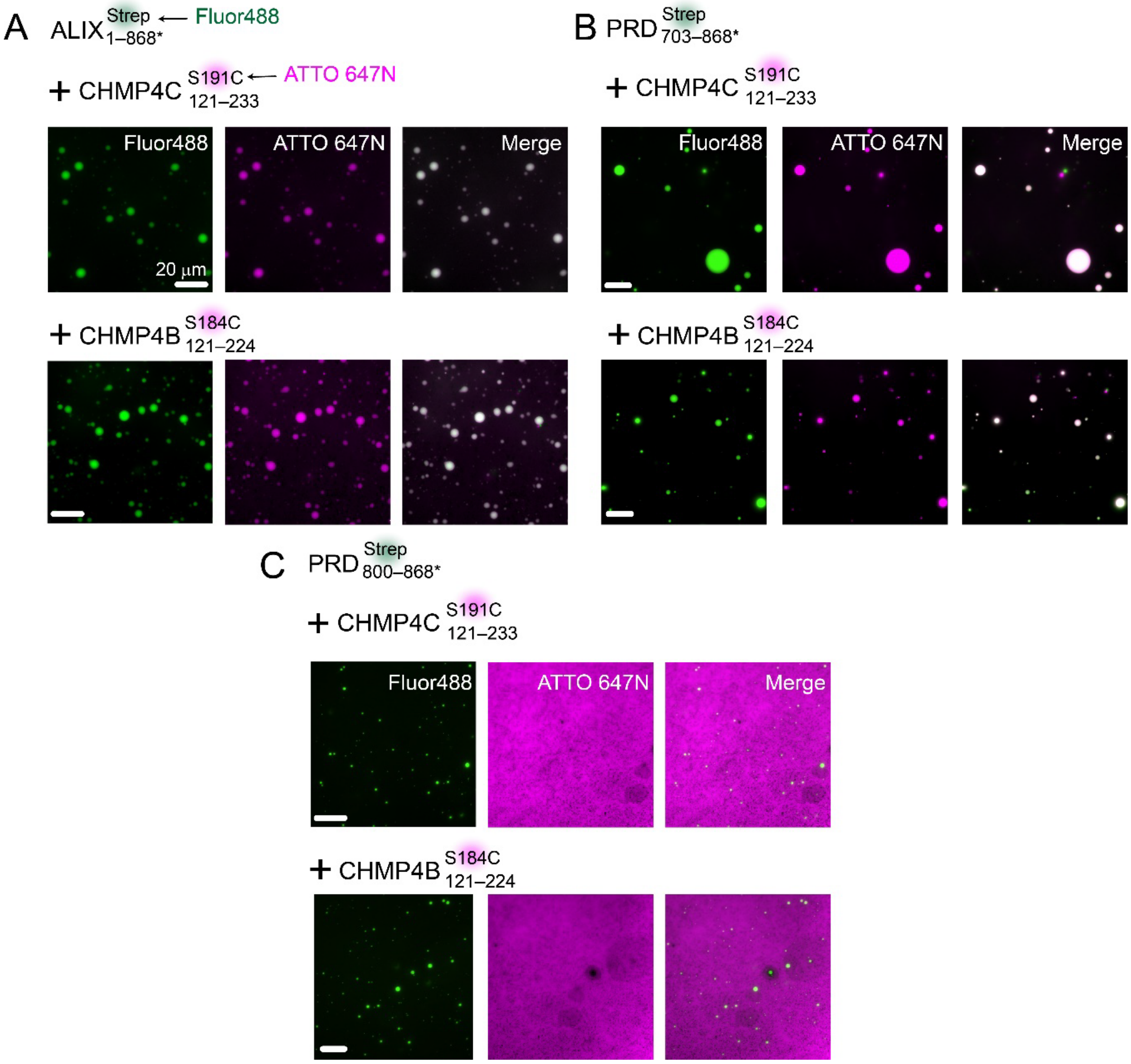

Figure S8. Panoramic images showing co-partitioning (or the lack thereof) of CHMP4 paralogs in condensates made by ALIX constructs. Representative fluorescence microscopy images showing colocalization of ATTO-647N labeled CHMP4 paralogs, $CHMP4C^{S191C}_{121-233}$ and $CHMP4B^{S184C}_{121-224}$, in Alexa-Fluor488-labeled droplets of (**A**) $ALIX^{Strep}_{1-868*}$, (**B**) $PRD^{Strep}_{703-868*}$, and a lack of their colocalization in Alexa-Fluor488-labeled $PRD^{Strep}_{800-868}$ droplets (panel **C**). Co-localization or lack thereof of CHMP4 paralogs was verified over n ≥ 3 replicates (≥ 100 condensates per sample). Protein and fluorophore concentrations were the same as those described in Fig. 3 (main text) caption.

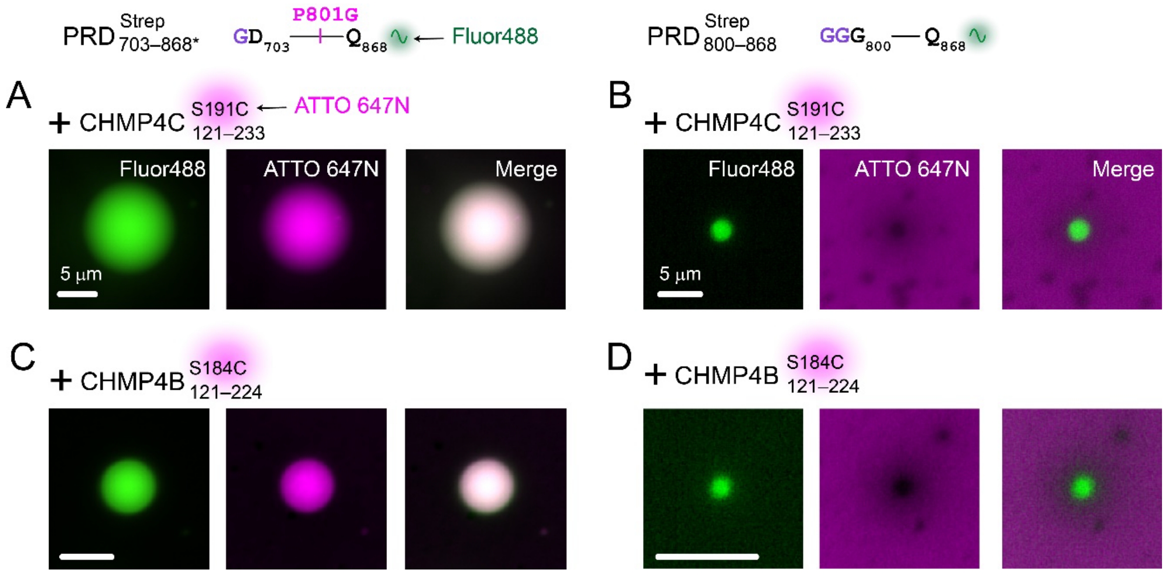

Figure S9. Co-partitioning of CHMP4 paralogs in condensates made by ALIX-PRD constructs. Representative fluorescence microscopy images showing colocalization of ATTO-647N labeled CHMP4 paralogs, $CHMP4C^{S191C}_{121-233}$ and $CHMP4B^{S184C}_{121-224}$, in Alexa-Fluor488-labeled $PRD^{Strep}_{703-868*}$ droplets (panels **A** and **C**), and a lack of their colocalization in Alexa-Fluor488-labeled $PRD^{Strep}_{800-868}$ droplets (panels **B** and **D**).

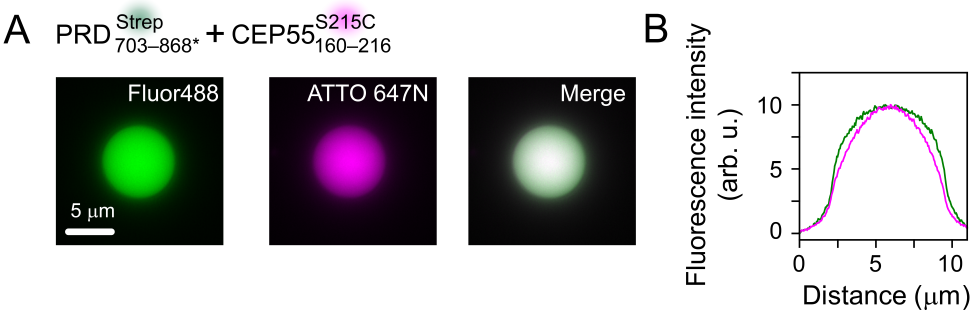

Figure S10. Co-partitioning of CEP55 in PRD$^{Strep}_{703–868*}$ condensates (**A**) Representative fluorescence microscopy images showing colocalization of CEP55$^{S215C}_{160–216}$ in PRD$^{Strep}_{703–868*}$ droplets. (**B**) Corresponding fluorescence intensity profiles. The binding interface between the two proteins was mapped by X-ray crystallography [PDB entry: 3E1R (*25*)] and comprises residues 178–195 of CEP55 and 797–808 of ALIX-PRD. A ready colocalization of CEP55$^{S215C}_{160–216}$ in PRD$^{Strep}_{703–868*}$ droplets, indicates that P801G mutation did not interfere with these interactions. Protein concentrations were the same as those described in Fig. 3 (main text) caption. In the case of PRD$^{Strep}_{703–868*}$, the concentration of Streptavidin Alexa-Fluor488 was 0.2 mg/mL. For CEP55$^{S215C}_{160–216}$, the concentration of ATTO 647N-labeled moeties were 5 molar percent.

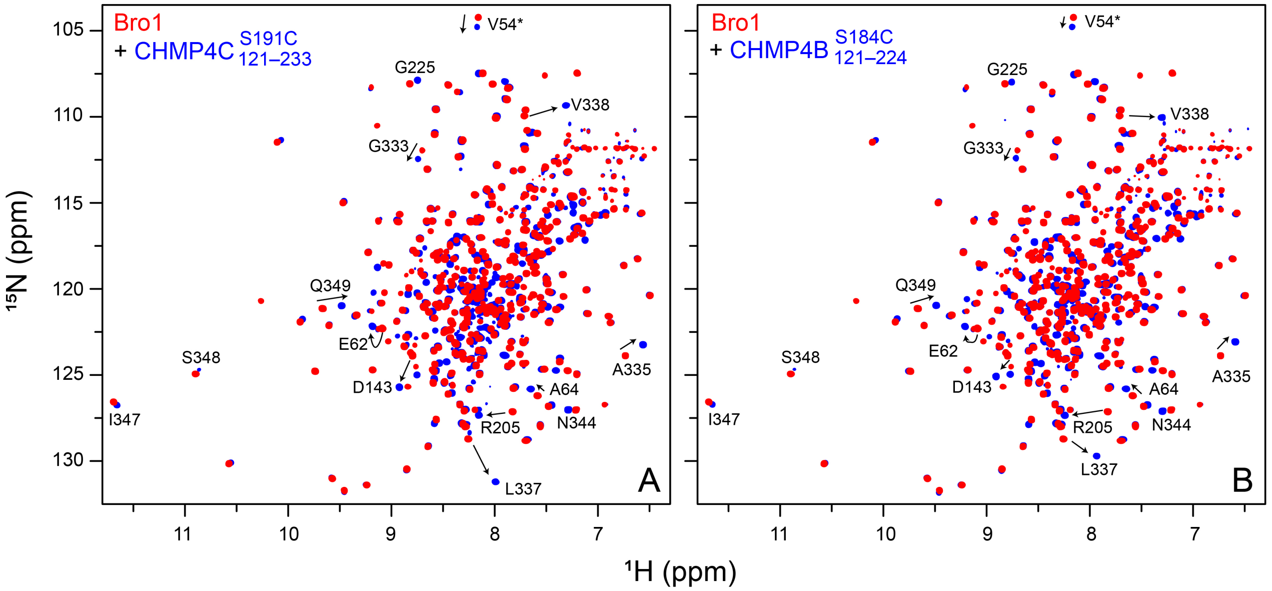

Figure S11. NMR analysis of interactions between ALIX-Bro1 and CHMP4 paralogs. Overlay of expanded region of the ^{1}H-^{15}N TROSY-HSQC correlation spectra of ^{15}N/^{2}H-labeled 100 μM Bro1 in the absence (red) and presence (blue) of 300 μM CHMP4 paralogs, namely (**A**) CHMP4C$^{S191C}_{121–233}$ and (**B**) CHMP4B$^{S184C}_{121–224}$. Some isolated cross-peaks of Bro1 that exhibit changes in chemical shifts upon addition of CHMP4 paralogs are labeled. Folded cross-peaks of residue V54 are marked by asterisks. Buffer and experimental conditions were as follows: 20 mM sodium phosphate, pH 6.5, 1 mM TCEP, and 2 mM EDTA at 30 °C.

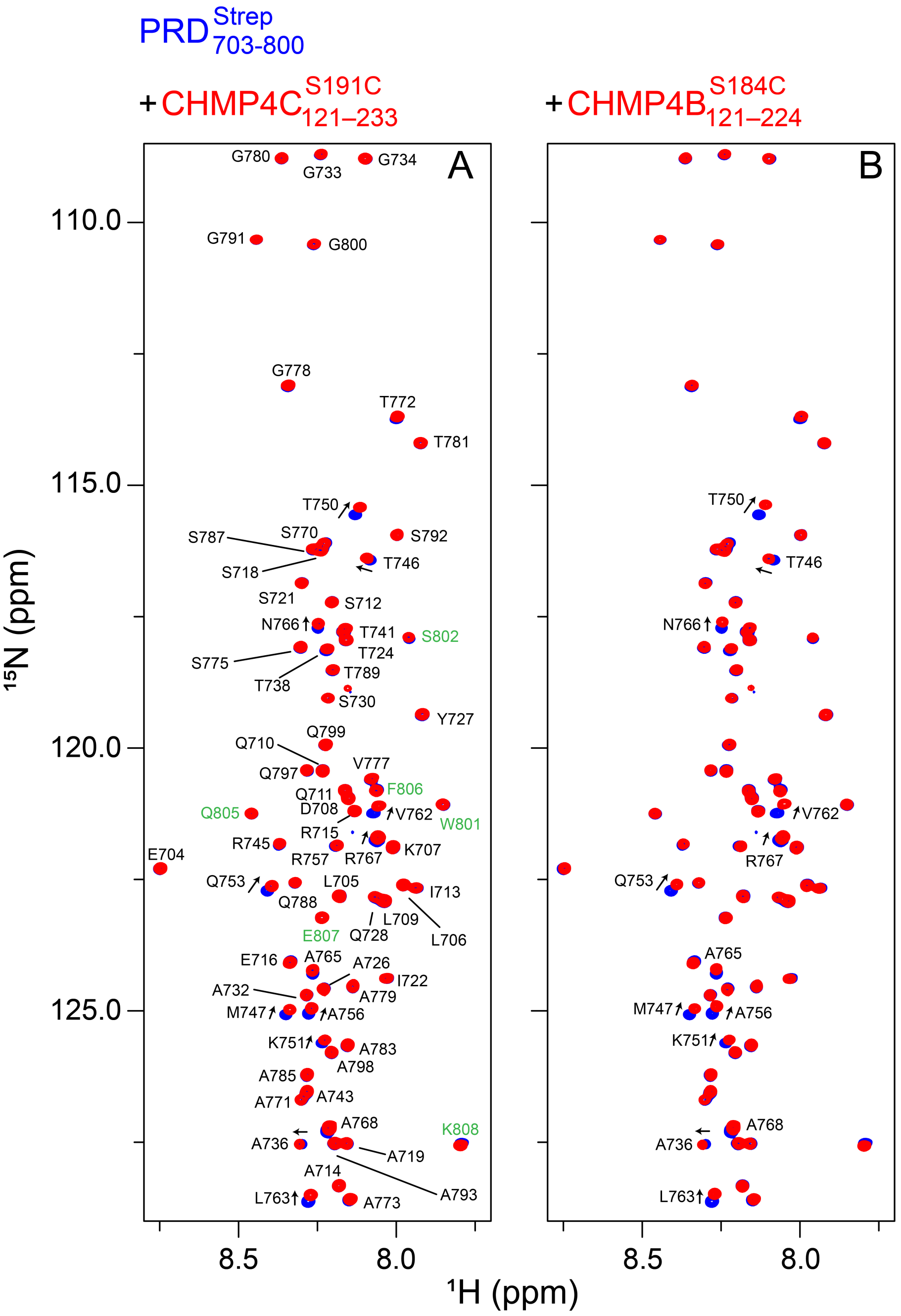

Figure S12. NMR analysis of interactions between PRD$^{Strep}_{703–800}$ and CHMP4 paralogs. Overlay of expanded region of the ^{1}H-^{15}N TROSY-HSQC correlation spectra of ^{15}N/^{13}C-labeled 150 µM PRD$^{Strep}_{703–800}$ in the absence (blue) and presence (red) of 450 µM CHMP4 paralogs, namely (**A**) CHMP4C$^{S191C}_{121–233}$ and (**B**) CHMP4B$^{S184C}_{121–224}$; also see Fig. S13 caption for the rationale used to select these protein concentrations. Assignments of each cross-peaks are shown in panel (**A**). Cross-peaks of the residues that undergo chemical shift changes upon addition of CHMP4 constructs are

marked by arrows. Non-native residues of the C-terminal Strep tag are labeled in green. Buffer and experimental conditions were as follows: 20 mM sodium phosphate, pH 6.5, 1 mM TCEP, and 2 mM EDTA at 30 °C.

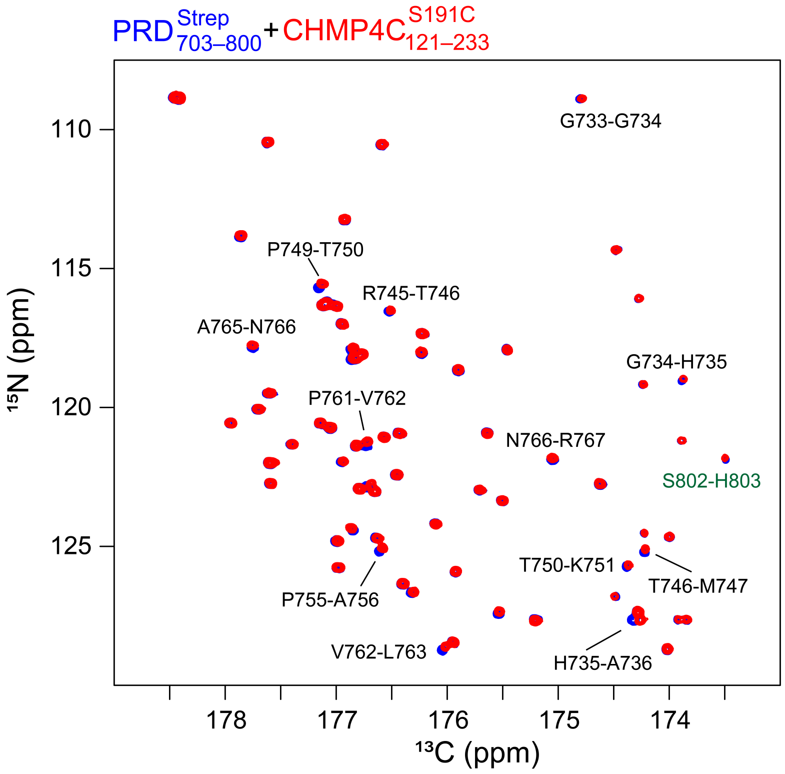

Figure S13. NMR analysis of interactions between PRD$^{Strep}_{703–800}$ and CHMP4C$^{S191C}_{121–233}$. Overlay of expanded region of the ^{13}C-^{15}N CON correlation spectra of ^{15}N/^{13}C-labeled 150 µM PRD$^{Strep}_{703–800}$ in the absence (blue) and presence (red) of 450 µM CHMP4C$^{S191C}_{121–233}$. Note that the above-mentioned concentrations were chosen because of the following: (1) poor sensitivity of the carbon-detected ^{13}C-^{15}N CON experiment as compared to its proton-detected counterpart (*69*); and (2) the addition of > 450 µM CHMP4 paralogs to 150 µM PRD$^{Strep}_{703–800}$ led to sample precipitation. Cross-peaks of the residues that undergo chemical shift changes upon addition of CHMP4C$^{S191C}_{121–233}$ are marked. Cross-peaks of non-native residues of the C-terminal strep tag are labeled in green. Buffer and experimental conditions were as follows: 20 mM sodium phosphate, pH 6.5, 1 mM TCEP, and 2 mM EDTA at 30 °C.

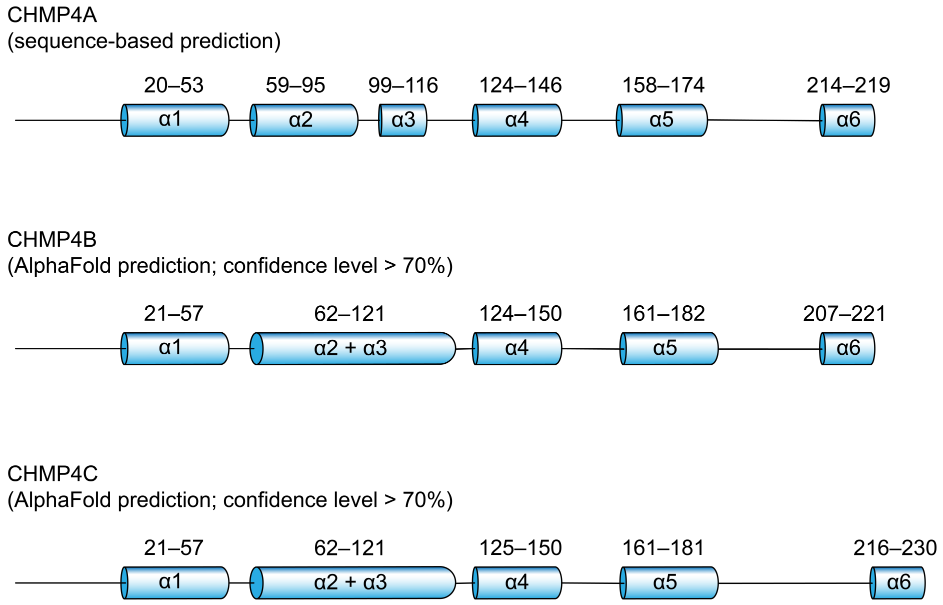

Figure S14. Predicted structural organization of human CHMP4 paralogs. Predictions based on the primary sequence [CHMP4A, upper (*33*)] or AlphaFold (*34*) [CHMP4B, middle, and CHMP4C, lower]. Cylinders and labels represent the corresponding CHMP4 helices (α1–α6) whereas numbers above the cylinders represent corresponding CHMP4 residues. Note that among the three CHMP4 paralogs, the cellular functions of CHMP4A are not known.

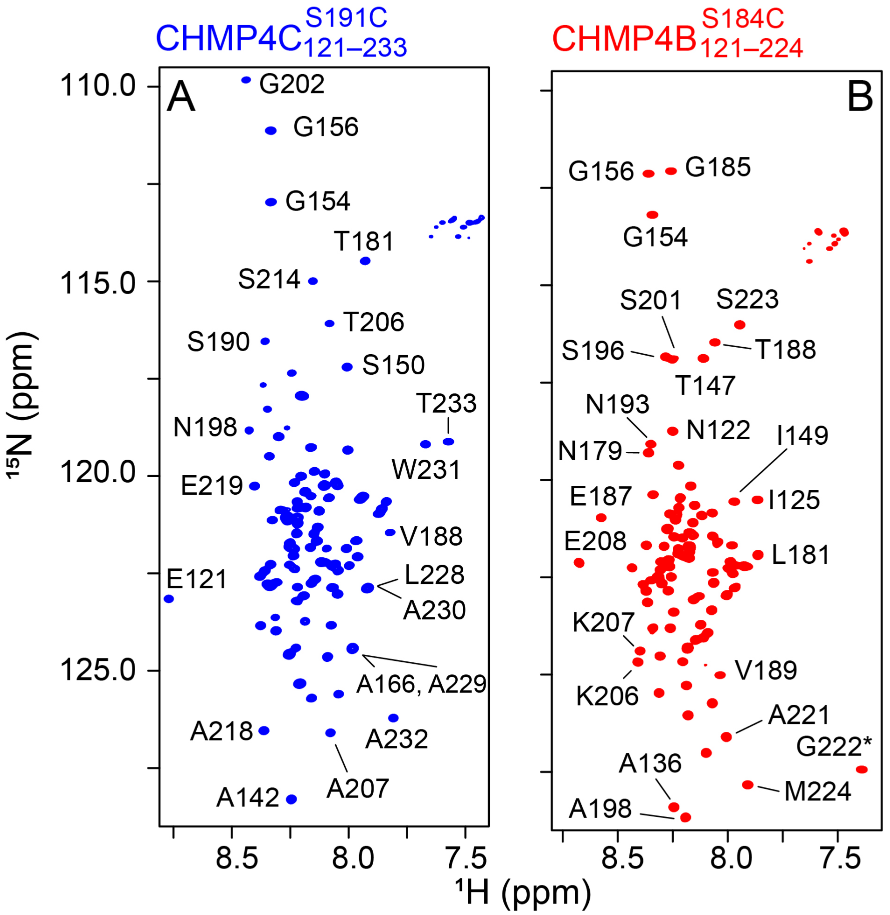

Figure S15. NMR spectra of CHMP4 paralogs. Expanded regions of the ^{1}H-^{15}N TROSY-HSQC correlation spectra of ^{15}N-labeled (**A**) CHMP4C$^{S191C}_{121-233}$ (blue) and (**B**) CHMP4B$^{S184C}_{121-224}$ (red). Some of the isolated cross-peaks are marked. Folded cross-peak of residue G222 (CHMP4B$^{S184C}_{121-224}$) is marked with an asterisk. Buffer and experimental conditions were as follows: 20 mM sodium phosphate, pH 6.5, 1 mM TCEP, and 2 mM EDTA at 30 °C. Protein concentrations were 0.5 mM each.

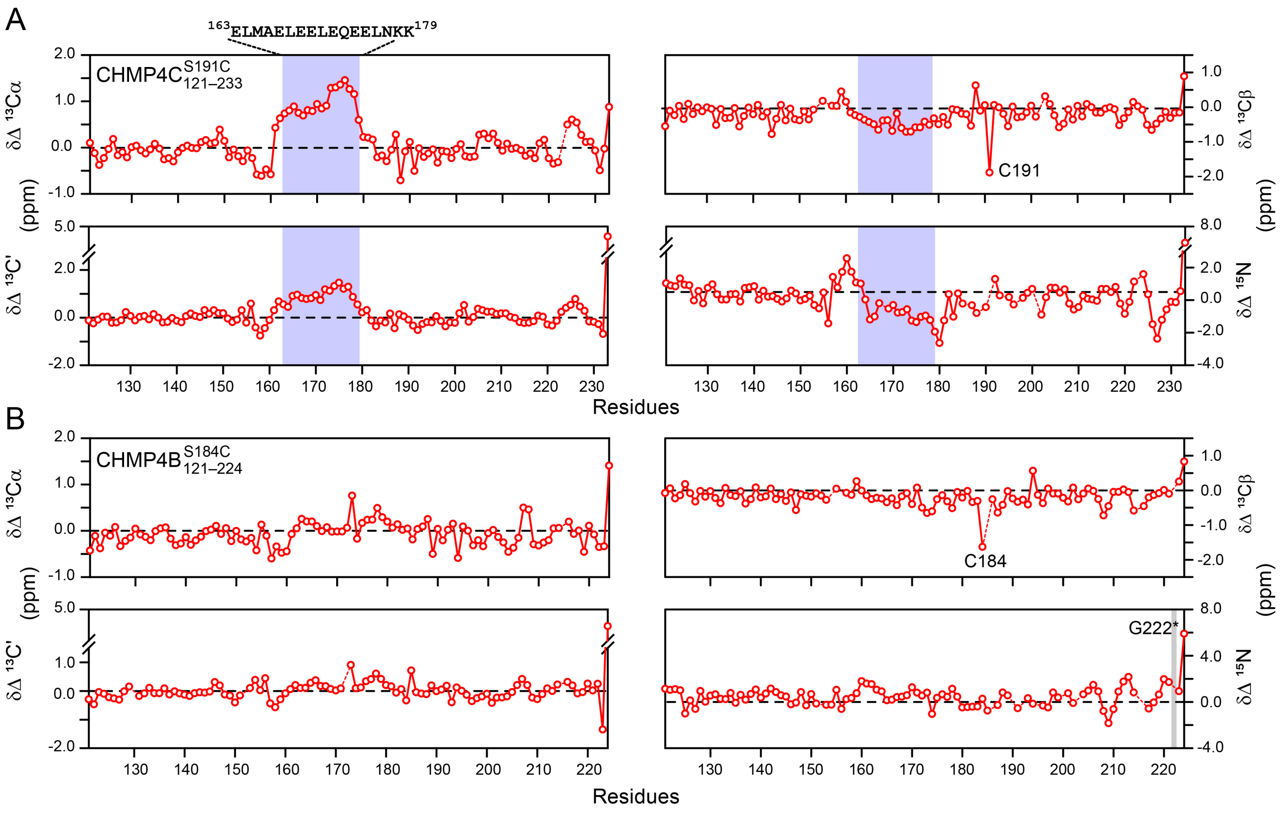

Figure S16. NMR analysis of CHMP4 paralogs. Secondary chemical shifts (Δδ), $^{13}C_{\alpha}$, $^{13}C'$, $^{13}C_{\beta}$ and ^{15}N, of (**A**) CHMP4C$^{S191C}_{121-233}$, and (**B**) CHMP4B$^{S184C}_{121-224}$, derived from assigned backbone chemical shifts and the corresponding random coil values and correction factors of Poulsen and co-workers (*35, 36*). The region between residues 163–179 of CHMP4C$^{S191C}_{121-233}$, which forms a stable helix, is highlighted in semi-transparent blue rectangles. The C_{β} chemical shifts of engineered cysteine residues, namely C191 of CHMP4C$^{S191C}_{121-233}$ and C184 of CHMP4B$^{S184C}_{121-224}$, were ca. 28 ppm, indicating that these two residues were reduced in solution (*80*). Δδ(^{15}N) of G222 of CHMP4B$^{S184C}_{121-224}$ was not considered because of its folded cross-peak (cf. Fig. S15).

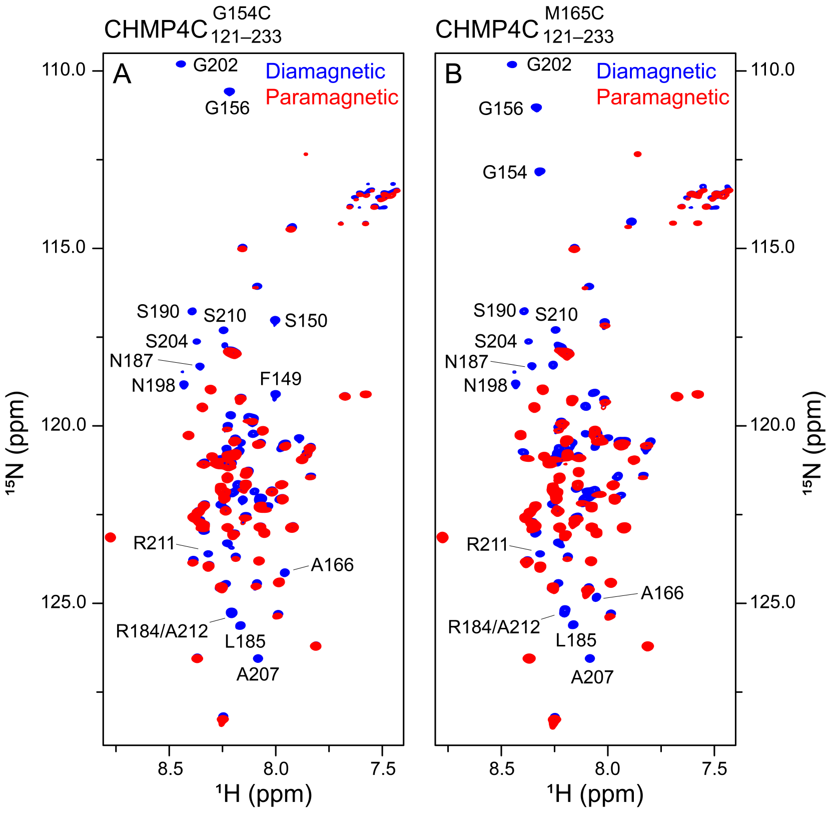

Figure S17. NMR-PRE analysis of the transient long-range interactions in CHMP4C constructs. Overlay of expanded regions of ^{1}H-^{15}N TROSY-HSQC spectra of MTSL-labeled (red, paramagnetic) and its acetylated analog-labeled (blue; diamagnetic) (**A**) CHMP4C$^{G154C}_{121-233}$ and (**B**) CHMP4C$^{M165C}_{121-233}$. A few of the isolated cross-peaks that undergo signal attenuation (^{1}H-^{15}N cross-peak heights ratio < 0.25) due to the PRE effect are labeled; note that intramolecular PREs are exquisitely sensitive and have thus been used to identify transient long-range interactions in disordered proteins (*39-41, 81, 82*). All spectra were recorded at 800 MHz at 30 °C. Buffer conditions were as follows: 20 mM sodium phosphate, pH 6.5, and 2 mM EDTA. Protein concentrations were 0.2 mM each.

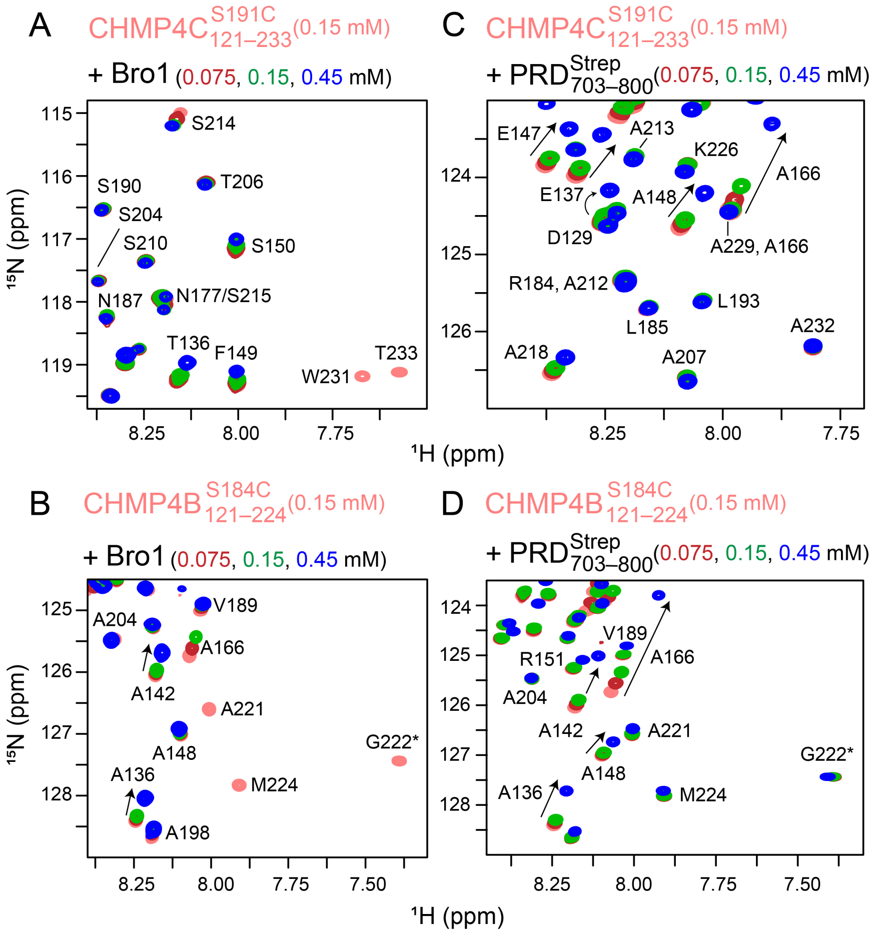

Figure S18. NMR analysis of interactions between CHMP4 paralogs and ALIX domains. Overlay of expanded regions of the ^{1}H-^{15}N TROSY-HSQC correlation spectra of ^{15}N-labeled (**A** and **C**) $CHMP4C^{S191C}_{121–233}$ and (**B** and **D**) $CHMP4B^{S184C}_{121–224}$ in the absence and presence of unlabeled ALIX domains, namely Bro1 and $PRD^{Strep}_{703–800}$. The concentrations of CHMP4 paralogs were 150 μM. The color scheme is as follows: CHMP4 paralogs in free form = light red, in the presence of 75, 150, and 450 μM of Bro1 / $PRD^{Strep}_{703–800}$ = dark red, green, and blue, respectively. A few of cross-peaks are marked. Cross-peaks of the residues that undergo chemical shift changes upon addition of $PRD^{Strep}_{703–800}$ are marked by arrows. Folded cross-peaks of residue G222 ($CHMP4B^{S184C}_{121–224}$) are marked with asterisks.

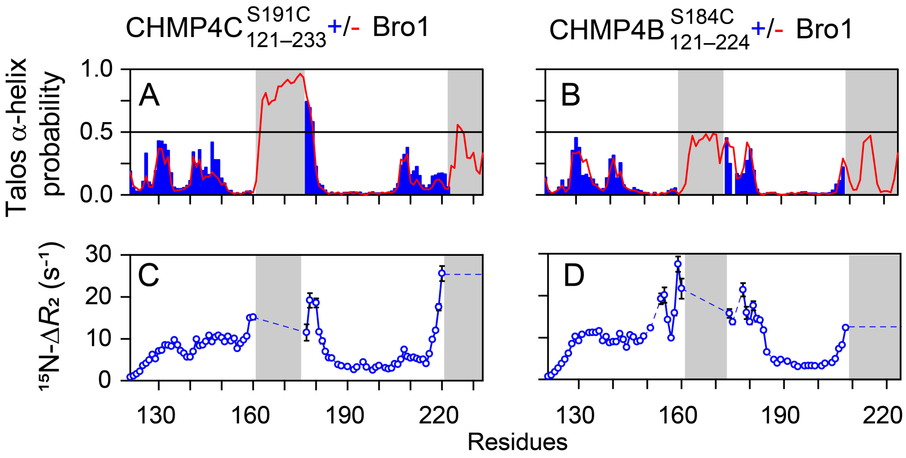

Figure S19. NMR analysis of CHMP4–Bro1 interactions. TALOS-N (*37*) derived helical propensities of (**A**) CHMP4C$^{S191C}_{121\text{-}233}$ and (**B**) CHMP4B$^{S184C}_{121\text{-}224}$, in the presence (blue bars) and absence (red lines) of Bro1; obtained from the corresponding assigned backbone chemical shifts, namely $^{13}C_{\alpha}$, $^{13}C_{\beta}$, $^{13}C'$, ^{15}N, and $^{1}H_{N}$. Residues that undergo resonance-line broadening in the presence of Bro1 are marked by semi-transparent grey rectangles. $^{15}N\text{-}\Delta R_2$ profiles of (**C**) CHMP4C$^{S191C}_{121\text{-}233}$ + Bro1 and (**D**) CHMP4B$^{S184C}_{121\text{-}224}$ + Bro1 samples at 800 MHz (30 ºC). Protein concentrations were as follows: 150 µM ^{15}N-labeled CHMP4 paralogs and 450 µM non-labeled Bro1.

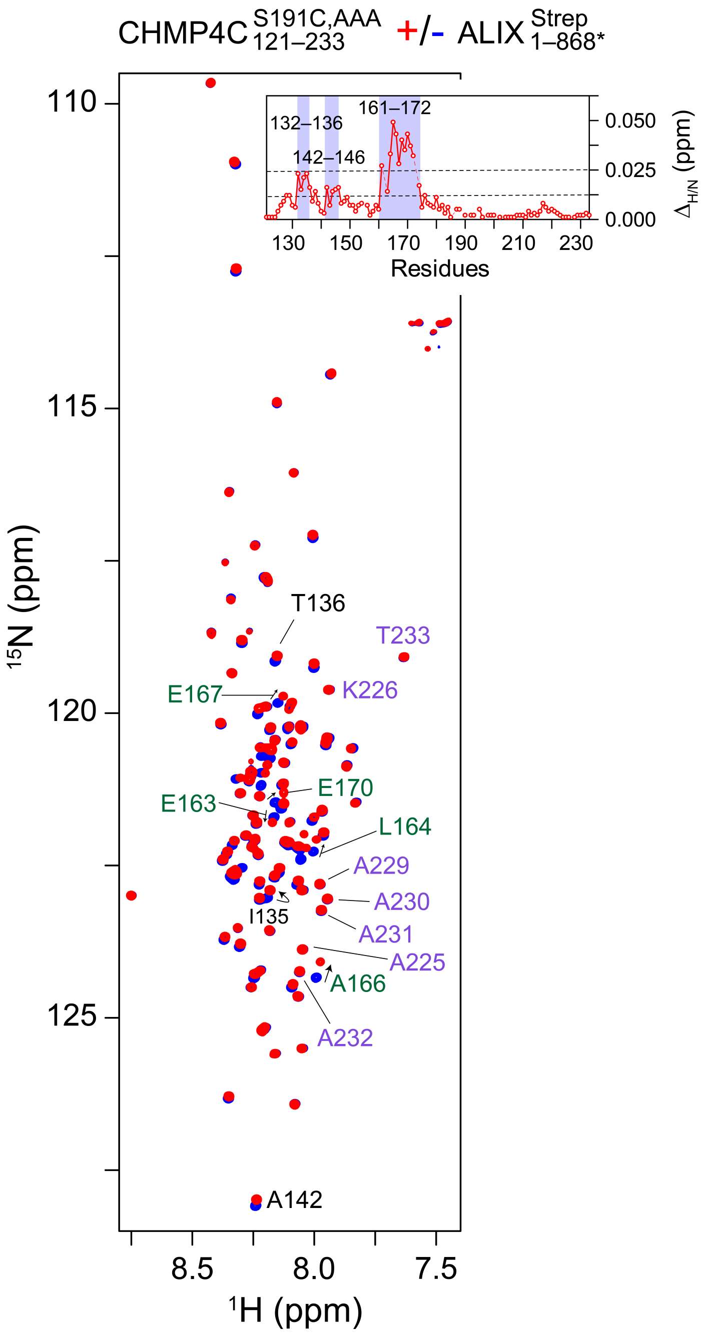

Figure S20. NMR analysis of CHMP4C$^{S191C,AAA}_{121-233}$–ALIX$^{Strep}_{1-868*}$ interactions. Overlay of expanded regions of the ^{1}H-^{15}N TROSY-HSQC correlation spectra of 30 μM $^{15}N/^{2}H$-labeled CHMP4C$^{S191C,AAA}_{121-233}$ in the absence (blue) and presence (red) of 90 μM unlabeled ALIX$^{Strep}_{1-868*}$. A few of the cross-peaks of α5 motif that show signal attenuation upon addition of ALIX$^{Strep}_{1-868*}$ are marked in green (^{1}H-^{15}N cross-peak heights ratio = 0.25–0.5). Cross-peaks of α6 motif that did not show any signal attenuation are marked in magenta. A few other cross-peaks that show chemical

shift perturbations are marked in black. **Inset:** Corresponding $^{1}H_{N}/^{15}N$ chemical shift perturbation profile; semi-transparent blue rectangles indicate regions that exhibit chemical shift perturbations ($\Delta_{H/N} \geq 0.0125$ ppm). Spectra were acquired at a spectrometer ^{1}H frequency of 800 MHz (30 °C). Buffer conditions were the same as those described in Fig. 4 (main text) caption.

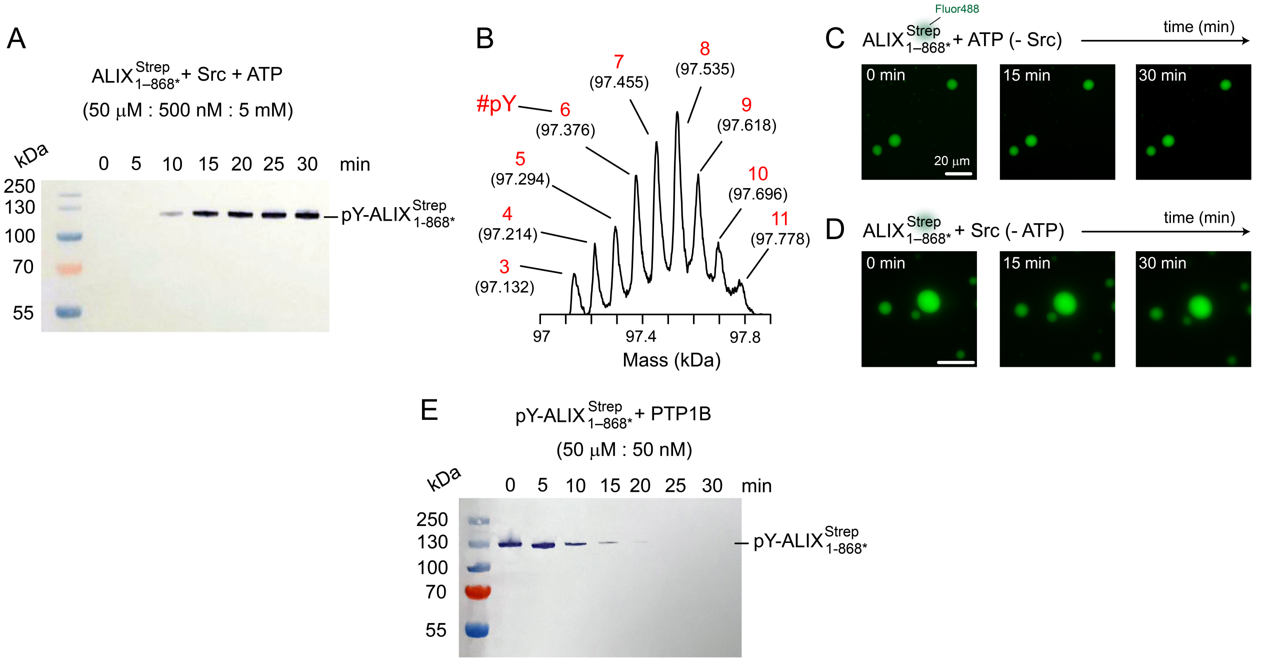

Figure S21. Reversible tyrosine phosphorylation of $\mathrm{ALIX}^{\mathrm{Strep}}_{1\text{–}868^*}$. (**A**) Time course of Src-mediated in vitro phosphorylation of $\mathrm{ALIX}^{\mathrm{Strep}}_{1\text{–}868^*}$ by western blotting (the primary and secondary antibodies were phospho-tyrosine mouse monoclonal and goat anti-mouse IgG, respectively). The numbers in parenthesis represent the concentrations of $\mathrm{ALIX}^{\mathrm{Strep}}_{1\text{–}868^*}$, Src, and ATP used for this experiment, namely 50 µM, 500 nM, and 5 mM, respectively. The gel-band for phosphorylated (pY) $\mathrm{ALIX}^{\mathrm{Strep}}_{1\text{–}868^*}$ is marked. Note that the corresponding band of phosphorylated Src is not detected because of its low nanomolar concentration, and that $\mathrm{ALIX}^{\mathrm{Strep}}_{1\text{–}868^*}$ migrates anomalously on 4–12% Bis-Tris SDS-PAGE gel, likely due to its disordered PRD. (**B**) LC–ESI–TOFMS analysis of phosphorylated $\mathrm{ALIX}^{\mathrm{Strep}}_{1\text{–}868^*}$ revealed hyperphosphorylated state of $\mathrm{ALIX}^{\mathrm{Strep}}_{1\text{–}868^*}$ [ALIX carries 33 native tyrosine residues among which 11 are localized in the C-terminal portion of its PRD, residues 803 to 846 (*23*)]. The numbers in red represent the number of phosphorylated tyrosine residues, labeled as pY. The numbers in parentheses represent the corresponding masses in kDa. Representative microscopy images establishing the lack of dissolution of Alexa-Fluor488-labeled $\mathrm{ALIX}^{\mathrm{Strep}}_{1\text{–}868^*}$ condensates in the absence of (**C**) Src, and (**D**) ATP. (**E**) Time course of dephosphorylation of 50 µM hyperphosphorylated $\mathrm{ALIX}^{\mathrm{Strep}}_{1\text{–}868^*}$ by 50 nM PTP1B monitored by western blotting. The buffer conditions were as follows: 50 mM Tris, pH 7.5, 50 mM NaCl, 2 mM DTT, 5 mM $MgCl_2$ for the kinase reaction, and 20 mM HEPES, pH 7.5, 50 mM NaCl, 1 mM DTT, 1 mM EDTA for the phosphatase reaction. Both reactions were carried out at room temperature.

Additionally, the phase-separation experiments shown in panels (**C**) and (**D**) were carried out in the presence of 5% (w/v) PEG-4000.

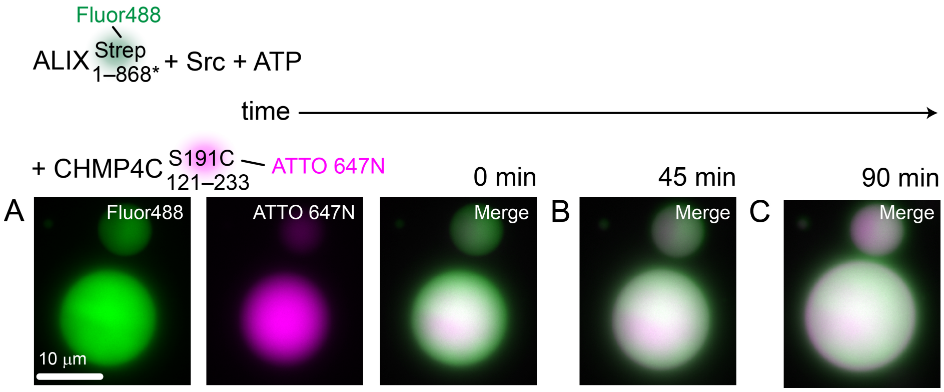

Figure S22. Time-course of the lack of Src-mediated dissolution of ALIX$^{Strep}_{1–868*}$ droplets in the presence of CHMP4C$^{S191C}_{121–233}$. Representative confocal images of Alexa-Fluor488-labeled ALIX$^{Strep}_{1–868*}$ condensates comprising CHMP4C$^{S191C}_{121–233}$ at three time points, namely 0 min (**A**), 45 min (**B**), and 90 min (**C**). The following concentrations were used: 50 µM ALIX$^{Strep}_{1–868*}$, 10 µM CHMP4C$^{S191C}_{121-233}$, 500 nM Src, and 5 mM ATP in the presence of 5% (w/v) PEG-4000.

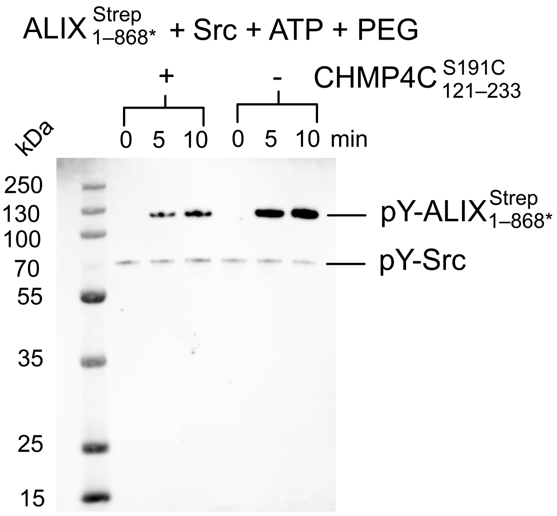

Figure S23. Western blot analysis of the changes in Src-mediated phosphorylation of ALIX$^{\mathrm{Strep}}_{1\text{–}868*}$ droplets with and without CHMP4C$^{\mathrm{S191C}}_{121\text{–}233}$. The primary and secondary antibodies were phospho-tyrosine mouse monoclonal and IRDye 800CW goat anti-mouse IgG, respectively. Blot was visualized using the Odyssey XF imaging system (LICOR Biosciences). The gel-bands for phosphorylated (pY) ALIX$^{\mathrm{Strep}}_{1\text{–}868*}$ and Src are marked. The following protein concentrations were used: 50 μM ALIX$^{\mathrm{Strep}}_{1\text{–}868*}$ with and without 50 μM CHMP4C$^{\mathrm{S191C}}_{121\text{-}233}$, and 5 μM Src. Buffer and experimental conditions were as follows: 50 mM Tris, pH 7.5, 50 mM NaCl, 5 mM $MgCl_2$, 2 mM DTT, 5 mM ATP, 5% (w/v) PEG-4000, and room temperature.

Table S1. Residue-specific NMR parameters of CHMP4 fragments in free form[a].

$CHMP4C^{S191C}_{121\text{-}233}$					$CHMP4B^{S184C}_{121\text{-}224}$				
Res.	α prop.[b]	$^3J_{HN\text{-}H\alpha}$ (Hz)[c] Pred.	Exp.	$^{15}N\text{-}R_2$ (s^{-1})[d]	Res.	α prop.[b]	$^3J_{HN\text{-}H\alpha}$ (Hz)[c] Pred.	Exp.	$^{15}N\text{-}R_2$ (s^{-1})[d]
E121	0.178	6.591	6.138	1.229 ± 0.036	D121	0.128	6.926	6.778	1.147 ± 0.037
N122	0.072	7.037	7.426	1.392 ± 0.041	N122	0.032	7.214	7.306	1.288 ± 0.042
M123	0.054	6.988	7.426	1.613 ± 0.044	M123	0.015	6.988	7.394	1.553 ± 0.037
D124	0.035	6.989	7.258	2.010 ± 0.033	D124	0.027	6.972	7.394	1.705 ± 0.029
L125	0.063	6.902	7.178	1.858 ± 0.021	I125	0.047	7.764	7.482	1.724 ± 0.019
N126	0.151	7.418	7.554	1.975 ± 0.036	D126	0.076	6.897	7.218	1.884 ± 0.022
K127	0.065	6.802	7.346	2.027 ± 0.029	K127	0.025	6.742	7.570	1.926 ± 0.020
I128	0.062	7.528	7.634	2.251 ± 0.020	V128	0.056	7.661	7.570	1.906 ± 0.017
D129	0.206	7.181	7.010	2.828 ± 0.025	D129	0.335	6.927	6.954	2.253 ± 0.023
D130	0.368	7.042	7.178	2.322 ± 0.026	E130	0.331	6.707	6.690	2.112 ± 0.012
L131	0.366	6.727	7.306	3.205 ± 0.028	L131	0.346	6.550	7.218	2.290 ± 0.022
M132	0.247	7.070	7.258	2.801 ± 0.024	M132	0.263	7.070	7.570	2.811 ± 0.022
Q133	0.303	6.958	7.258	2.464 ± 0.031	Q133	0.282	7.101	7.218	2.397 ± 0.022
E134	0.090	6.429	6.930	2.846 ± 0.021	D134	0.179	6.764	7.130	2.218 ± 0.032
I135	0.032	7.441	7.594	2.771 ± 0.022	I135	0.084	7.722	7.570	2.115 ± 0.019
T136	0.038	7.747	7.842	2.816 ± 0.032	A136	0.075	6.119	6.154	2.525 ± 0.026
E137	0.041	6.498	6.890	2.726 ± 0.031	D137	0.046	6.746	6.954	2.140 ± 0.034
Q138	0.046	6.781	7.178	2.665 ± 0.033	Q138	0.034	6.958	7.306	2.094 ± 0.027
Q139	0.063	6.893	7.258	2.632 ± 0.026	Q139	0.071	6.750	7.042	2.149 ± 0.030
D140	0.083	6.764	7.218	2.656 ± 0.024	E140	0.204	6.446	6.690	2.366 ± 0.025
I141	0.306	7.722	7.386	2.693 ± 0.024	L141	0.293	6.771	6.866	2.405 ± 0.020
A142	0.220	5.923	6.010	3.391 ± 0.021	A142	0.146	6.057	6.154	2.312 ± 0.022
Q143	0.283	6.916	6.970	2.977 ± 0.034	E143	0.186	6.464	6.690	2.248 ± 0.030
E144	0.122	6.429	6.842	2.979 ± 0.026	E144	0.070	6.513	6.690	–
I145	0.149	7.517	6.970	3.304 ± 0.025	I145	0.051	7.517	7.570	2.323 ± 0.023
S146	0.143	6.957	6.970	3.417 ± 0.035	S146	0.038	6.954	6.690	2.540 ± 0.042
E147	0.182	6.590	6.426	3.384 ± 0.034	T147	0.038	7.634	7.746	2.326 ± 0.043
A148	0.153	6.091	5.762	3.423 ± 0.037	A148	0.026	5.955	5.978	2.449 ± 0.033
F149	0.129	7.208	7.138	3.256 ± 0.030	I149	0.017	7.599	7.746	2.114 ± 0.030
S150	0.080	7.146	6.762	3.261 ± 0.045	S150	0.017	6.816	6.954	2.248 ± 0.030
Q151	0.097	6.809	7.178	3.333 ± 0.035	K151	0.015	6.772	7.218	2.332 ± 0.034
R152	0.035	6.692	7.514	3.257 ± 0.028	P152	0.003	–	–	–
V153	0.023	7.749	7.722	3.257 ± 0.029	V153	0.017	7.623	7.218	2.175 ± 0.021
G154	0.008	–	–	3.191 ± 0.031	G154	0.000	–	–	2.277 ± 0.026
F155	0.017	7.349	7.554	3.009 ± 0.027	F155	0.019	7.349	7.130	2.122 ± 0.035

Table S1 (cont'd).

$CHMP4C^{S191C}_{121\text{-}233}$					$CHMP4B^{S184C}_{121\text{-}224}$				
Res.	α prop.[b]	$^3J_{HN\text{-}H\alpha}$ (Hz)[c] Pred.	Exp.	^{15}N-R_2 (s^{-1})[d]	Res.	α prop.[b]	$^3J_{HN\text{-}H\alpha}$ (Hz)[c] Pred.	Exp.	^{15}N-R_2 (s^{-1})[d]
G156	0.008	–	–	2.886 ± 0.035	G156	0.009	–	–	2.002 ± 0.037
D157	0.015	7.049	7.218	3.236 ± 0.032	E157	0.023	6.571	7.218	2.140 ± 0.024
D158	0.030	7.330	7.426	3.327 ± 0.037	E158	0.038	6.818	7.042	2.156 ± 0.020
F159	0.024	7.373	7.258	4.193 ± 0.037	F159	0.036	7.196	7.746	2.393 ± 0.020
D160	0.021	7.280	7.258	4.413 ± 0.028	D160	0.006	7.280	7.394	2.561 ± 0.030
E161	0.110	6.702	5.306	4.940 ± 0.027	E161	0.036	6.702	6.514	2.814 ± 0.028
D162	0.451	6.717	7.050	3.836 ± 0.029	D162	0.091	6.717	6.866	3.349 ± 0.018
E163	0.757	6.707	5.642	6.061 ± 0.033	E163	0.319	6.707	6.690	3.191 ± 0.022
L164	0.812	6.550	5.554	6.162 ± 0.012	L164	0.471	6.550	6.602	3.331 ± 0.025
M165	0.718	7.224	6.474	5.720 ± 0.027	M165	0.388	7.224	7.394	2.893 ± 0.023
A166	0.748	5.779	–	–	A166	0.434	5.779	5.626	3.729 ± 0.022
E167	0.756	6.612	5.346	6.018 ± 0.044	E167	0.448	6.612	6.602	3.438 ± 0.029
L168	0.865	6.670	–	–	L168	0.485	6.670	6.954	3.673 ± 0.010
E169	0.865	6.784	4.185	6.490 ± 0.027	E169	0.433	6.784	6.602	3.857 ± 0.016
E170	0.894	6.530	4.265	7.811 ± 0.086	E170	0.487	6.530	6.778	2.422 ± 0.029
L171	0.919	6.670	3.929	6.906 ± 0.039	L171	0.481	6.670	6.602	3.639 ± 0.024
E172	0.888	6.731	4.969	6.333 ± 0.033	E172	0.252	6.731	6.778	3.663 ± 0.021
Q173	0.896	6.834	4.137	7.075 ± 0.053	Q173	0.371	6.834	6.602	2.276 ± 0.022
E174	0.937	6.298	3.513	6.429 ± 0.034	E174	0.446	6.298	6.690	2.473 ± 0.010
E175	0.964	6.530	4.889	6.305 ± 0.040	E175	0.387	6.530	7.042	3.689 ± 0.021
L176	0.934	6.725	3.977	6.176 ± 0.024	L176	0.378	6.813	6.242	3.244 ± 0.027
N177	0.793	7.418	5.386	4.959 ± 0.042	D177	0.131	6.978	–	2.916 ± 0.025
K178	0.692	6.530	5.306	5.760 ± 0.044	K178	0.140	6.779	6.154	3.544 ± 0.035
K179	0.529	6.368	5.890	4.599 ± 0.036	N179	0.221	7.246	6.866	3.429 ± 0.035
M180	0.192	6.659	6.098	5.012 ± 0.030	L180	0.418	6.942	8.282	2.956 ± 0.013
T181	0.070	7.605	7.050	4.275 ± 0.045	L181	0.356	7.072	7.130	2.802 ± 0.020
N182	0.050	7.457	6.594	4.457 ± 0.031	E182	0.176	6.915	6.954	2.680 ± 0.022
I183	0.031	7.436	7.554	3.437 ± 0.029	I183	0.030	7.557	7.842	2.324 ± 0.023
R184	0.009	7.227	8.090	3.914 ± 0.036	C184	0.007	7.294	7.658	2.375 ± 0.038
L185	0.014	6.853	7.218	3.679 ± 0.027	G185	0.005	–	–	2.090 ± 0.044
P186	0.012	–	–	–	P186	0.017	–	–	–
N187	0.020	7.114	7.138	3.257 ± 0.028	E187	0.017	6.318	6.690	2.317 ± 0.032
V188	0.005	7.819	7.970	3.052 ± 0.036	T188	0.017	7.444	7.746	2.270 ± 0.033
P189	0.017	–	–	–	V189	0.007	7.864	7.746	2.567 ± 0.026
S190	0.012	6.688	6.031	3.000 ± 0.039	P190	0.003	–	–	–

Table S1 (cont'd).

$CHMP4C^{S191C}_{121\text{-}233}$					$CHMP4B^{S184C}_{121\text{-}224}$				
Res.	α prop.[b]	$^3J_{HN\text{-}H\alpha}$ (Hz)[c] Pred.	Exp.	^{15}N-R_2 (s^{-1})[d]	Res.	α prop.[b]	$^3J_{HN\text{-}H\alpha}$ (Hz)[c] Pred.	Exp.	^{15}N-R_2 (s^{-1})[d]
C191	0.020	7.046	–	–	L191	0.004	6.727	6.954	2.471 ± 0.010
S192	0.011	7.103	6.542	2.833 ± 0.047	P192	0.009	–	–	–
L193	0.023	6.895	7.218	3.045 ± 0.038	N193	0.019	7.114	7.394	2.266 ± 0.051
P194	0.008	–	–	–	V194	0.006	7.819	7.218	2.950 ± 0.025
A195	0.008	5.541	6.050	3.050 ± 0.043	P195	0.018	–	–	–
Q196	0.001	7.034	7.178	2.883 ± 0.037	S196	0.012	6.706	6.514	2.302 ± 0.036
P197	0.019	–	–	–	I197	0.020	7.652	7.658	2.319 ± 0.034
N198	0.015	6.964	6.858	3.511 ± 0.035	A198	0.010	6.124	6.514	2.566 ± 0.034
R199	0.012	6.741	6.570	3.635 ± 0.028	L199	0.010	6.870	7.218	2.430 ± 0.031
K200	0.008	6.730	6.978	3.042 ± 0.025	P200	0.008	–	–	–
P201	0.018	–	–	–	S201	0.005	6.434	6.602	2.325 ± 0.054
G202	0.015	–	–	3.711 ± 0.035	K202	0.012	6.772	6.866	2.516 ± 0.036
M203	0.022	6.983	5.033	–	P203	0.011	–	–	–
S204	0.019	6.833	4.553	3.392 ± 0.049	A204	0.009	5.453	5.626	2.422 ± 0.037
S205	0.028	6.740	6.330	3.558 ± 0.074	K205	0.012	6.488	6.690	2.609 ± 0.021
T206	0.066	7.634	6.050	3.962 ± 0.061	K206	0.022	6.347	6.242	2.861 ± 0.037
A207	0.283	5.692	4.553	4.416 ± 0.042	K207	0.136	6.488	5.274	2.987 ± 0.038
R208	0.270	6.708	6.170	4.210 ± 0.035	E208	0.291	6.323	5.626	3.171 ± 0.032
R209	0.324	6.896	5.930	4.457 ± 0.042	E209	0.223	6.382	7.042	2.824 ± 0.038
S210	0.103	6.569	5.202	4.304 ± 0.036	E210	0.108	6.525	6.514	2.316 ± 0.025
R211	0.080	6.966	4.385	4.272 ± 0.040	D211	0.041	6.860	7.042	2.398 ± 0.034
A212	0.123	5.821	6.570	3.828 ± 0.037	D212	0.042	7.037	6.514	2.408 ± 0.032
A213	0.083	5.810	4.873	3.195 ± 0.040	D213	0.119	6.774	–	2.916 ± 0.025
S214	0.047	6.791	6.050	2.839 ± 0.049	M214	0.419	6.757	7.130	2.487 ± 0.026
S215	0.044	6.690	5.762	–	K215	–	6.671	–	–
Q216	0.045	6.809	6.938	2.900 ± 0.030	E216	0.472	6.471	–	–
R217	0.064	6.775	7.058	2.761 ± 0.034	L217	0.293	6.670	6.954	2.310 ± 0.023
A218	0.117	5.720	6.050	2.935 ± 0.027	E218	0.088	6.839	6.602	2.138 ± 0.023
E219	0.125	6.464	6.530	2.794 ± 0.028	N219	0.023	7.638	7.482	1.983 ± 0.043
E220	0.098	6.382	7.058	3.179 ± 0.029	W220	0.030	6.863	6.514	1.806 ± 0.033
E221	0.053	6.525	7.138	2.363 ± 0.029	A221	0.031	6.331	6.514	1.768 ± 0.032
D222	0.046	6.860	7.178	2.652 ± 0.023	G222	0.021	–	–	1.387 ± 0.029
D223	0.232	7.037	–	–	S223	0.128	6.705	7.546	1.218 ± 0.040
D224	0.214	7.025	6.898	2.921 ± 0.025	M224	0.333	6.926	7.762	0.826 ± 0.007
I225	0.559	7.480	7.138	2.941 ± 0.022	–	–	–	–	–

Table S1 (cont'd).

$CHMP4C^{S191C}_{121\text{-}233}$					$CHMP4B^{S184C}_{121\text{-}224}$				
Res.	α prop.[b]	$^{3}J_{HN\text{-}H\alpha}$ (Hz)[c] Pred.	Exp.	$^{15}N\text{-}R_2$ (s^{-1})[d]	Res.	α prop.[b]	$^{3}J_{HN\text{-}H\alpha}$ (Hz)[c] Pred.	Exp.	$^{15}N\text{-}R_2$ (s^{-1})[d]
K226	0.529	6.815	6.738	3.117 ± 0.023	–	–	–	–	–
Q227	0.486	6.923	6.938	2.897 ± 0.032	–	–	–	–	–
L228	0.296	6.687	7.258	2.411 ± 0.027	–	–	–	–	–
A229	0.292	6.158	–	–	–	–	–	–	–
A230	0.336	6.218	6.858	2.292 ± 0.029	–	–	–	–	–
W231	0.199	6.821	7.426	1.813 ± 0.029	–	–	–	–	–
A232	0.168	6.221	7.098	1.611 ± 0.025	–	–	–	–	–
T233	0.333	7.606	8.106	0.914 ± 0.010	–	–	–	–	–

a. All NMR data were acquired at 30 ºC in buffer containing 20 mM sodium phosphate, pH 6.5, 1 mM TCEP, and 2 mM EDTA.

b. Helical propensities (α prop.) were calculated using Talos-N (*37*) from assigned backbone chemical shifts, namely $^{13}C_{\alpha}$, $^{13}C_{\beta}$, $^{13}C'$, ^{15}N, and $^{1}H_{N}$.

c. Experimental $^{3}J_{HN\text{-}H\alpha}$ couplings were measured using WATERGATE-optimized 2D TROSY pulse sequence (*67*). Corresponding random coil values were predicted using the nearest-neighbor effects corrected for temperature (*38*). Experimental $^{3}J_{HN\text{-}H\alpha}$ couplings of overlapped residues and glycines were not considered.

d. ^{15}N $R_{1\rho}$ measurements were carried out at 800 MHz on 0.5 mM proteins. Overlapped residues or residues that undergo resonance broadening due to rapid exchange were not considered.

Table S2. Summary of thermodynamic parameters for the interactions between CHMP4 paralogs and $ALIX^{Strep}_{1–868*}$ /Bro1 obtained by fitting ITC data to independent sites model[a,b].

	K_D (μM)	n	ΔH (kcal/mol)	-TΔS (kcal/mol)
$CHMP4C^{S191C}_{121-233}$				
+ $ALIX^{Strep}_{1–868*}$	0.6 ± 0.1	1.00 ± 0.02	-4.8 ± 0.2	-3.7 ± 0.3
+ $ALIX^{Strep}_{1–868*}$ (with 150 mM NaCl)[c]	6.1 ± 0.7	1.09 ± 0.01	-3.0 ± 0.2	-4.1 ± 0.3
+ Bro1	0.9 ± 0.1	1.20 ± 0.10	-5.0 ± 0.1	-3.3 ± 0.1
$CHMP4B^{S184C}_{121–224}$				
+ $ALIX^{Strep}_{1–868*}$	0.9 ± 0.1	1.05 ± 0.04	-6.4 ± 0.8	-1.8 ± 0.9
+ Bro1				
$CHMP4C^{S191C, AAA}_{121-233}$				
+ $ALIX^{Strep}_{1–868*}$ (with 150 mM NaCl)[c]		No binding was detected.		

a. All ITC measurements were performed at 25°C; ΔH = enthalpy and -TΔS = entropy of dissociation.
b. Errors were calculated from two-three independent titrations, performed by varying the concentrations of involved proteins.
c. To determine the impact of salt on ALIX – CHMP4C interactions, additional ITC measurements were performed in the presence of 150 mM sodium chloride; note that all the other ITC measurements were carried out in the absence of sodium chloride. For $ALIX^{Strep}_{1–868*}$ – $CHMP4C^{S191C,AAA}_{121-233}$ interactions, the sample concentrations were 1500 μM $CHMP4C^{S191C,AAA}_{121-233}$ and 300 μM $ALIX^{Strep}_{1–868*}$. All the remaining measurements were performed using 300–500 μM CHMP4 paralogs, $CHMP4C^{S191C}_{121–233}$ and $CHMP4B^{S184C}_{121–224}$, and 30–50 μM $ALIX^{Strep}_{1–868*}$ or Bro1.

Table S3. Primers used for mammalian expression.

Construct	Forward primer	Reverse primer
pmNeonGreenN1	TAAGCTTGGTACCGAGCT CGGATCCATGGTGAGCAA GGGCGAG	ACTGTGCTGGATATCTGCA GAATTCCATCACATCGGTA AAGGC
$ALIX^{mNG}_{1-868*}$	CTGGCTAGCGTTTAAACT TAAGCTTATGCATCACCA TCACCATCATGG	CTGGCTAGCGTTTAAACT TAAGCTTATGCATCACCA TCACCATCATGG
$ALIX^{mNG}_{1-702}$	CTGGCTAGCGTTTAAACT TAAGCTTATGCATCACCA TCACCATCATGG	CCTCGCCCTTGCTCACCA TGGATCCGCGTTCGGTTT TGCG
$ALIX^{mNG}_{1-868}$ (for G801P mutation)	GCCCCCCATATCCGACGTAC	GTCGGATATGGGGGGCCTTG

Movie S1. Src-mediated dissolution of $\mathbf{ALIX^{Strep}_{1\text{–}868*}}$ condensates monitored by fluorescence microscopy. Timelapse of dissolution of $ALIX^{Strep}_{1\text{–}868*}$ (50 μM) condensates in the presence of Src (5 μM) and ATP (5 mM). Microscopy images of the condensates were collected every min (30 min total). Video playback is 10 images/s. Buffer and experimental conditions were as follows: 50 mM Tris, pH 7.5, 50 mM NaCl, 5 mM $MgCl_2$, 2 mM DTT, 5% (w/v) PEG-4000, and 0.02 mg/mL Streptavidin Alexa-Fluor488, at room temperature.

Movie S2. Dissolution of $\mathbf{PRD^{Strep}_{703\text{–}868*}}$ condensates monitored by TIRF microscopy. Condensates of ATTO-488 labeled $PRD^{Strep}_{703\text{–}868*}$ (100 μM) were incubated with Src (10 μM) and ATP (5 mM). TIRF images of the condensates were collected every 15 s (80 min total). Video playback is sped up 450 times. Buffer and experimental conditions were as follows: 50 mM Tris, pH 7.5, 50 mM NaCl, 5 mM $MgCl_2$, 2 mM DTT, and 5% (w/v) PEG-4000, at room temperature.

REFERENCES AND NOTES

1. B. Mierzwa, D. W. Gerlich, Cytokinetic abscission: Molecular mechanisms and temporal control. *Dev. Cell* **31**, 525–538 (2014).

2. M. Vietri, M. Radulovic, H. Stenmark, The many functions of ESCRTs. *Nat. Rev. Mol. Cell Biol.* **21**, 25–42 (2020).

3. J. McCullough, R. D. Fisher, F. G. Whitby, W. I. Sundquist, C. P. Hill, ALIX-CHMP4 interactions in the human ESCRT pathway. *Proc. Natl. Acad. Sci. U.S.A.* **105**, 7687–7691 (2008).

4. S. Tang, N. J. Buchkovich, W. M. Henne, S. Banjade, Y. J. Kim, S. D. Emr, ESCRT-III activation by parallel action of ESCRT-I/II and ESCRT-0/Bro1 during MVB biogenesis. *eLife* **5**, e15507 (2016).

5. P. Steigemann, C. Wurzenberger, M.H.A. Schmitz, M. Held, J. Guizetti, S. Maar, D.W. Gerlich, Aurora B-mediated abscission checkpoint protects against tetraploidization. *Cell* **136**, 473–484 (2009).

6. V. Nähse, L. Christ, H. Stenmark, C. Campsteijn, The abscission checkpoint: Making it to the final cut. *Trends Cell Biol.* **27**, 1–11 (2017).

7. E. Petsalaki, G. Zachos, The abscission checkpoint: A guardian of chromosomal stability. *Cell* **10**, 3350 (2021).

8. J. G. Carlton, A. Caballe, M. Agromayor, M. Kloc, J. Martin-Serrano, ESCRT-III governs the Aurora B-mediated abscission checkpoint through CHMP4C. *Science* **336**, 220–225 (2012).

9. P. D. P. Pharoah, Y.-Y. Tsai, S. J. Ramus, C. M. Phelan, E. L. Goode, K. Lawrenson, M. Buckley, B. L. Fridley, J. P. Tyrer, H. Shen, R. Weber, R. Karevan, M. C. Larson, H. Song, D. C. Tessier, F. Bacot, D. Vincent, J. M. Cunningham, J. Dennis, E. Dicks; Australian Cancer Study; Australian Ovarian Cancer Study Group; K. K. Aben, H. Anton-Culver, N. Antonenkova, S. M. Armasu, L. Baglietto, E. V. Bandera, M. W. Beckmann, M. J. Birrer, G.

Bloom, N. Bogdanova, J. D. Brenton, L. A. Brinton, A. Brooks-Wilson, R. Brown, R. Butzow, I. Campbell, M. E. Carney, R. S. Carvalho, J. Chang-Claude, Y. A. Chen, Z. Chen, W.-H. Chow, M. S. Cicek, G. Coetzee, L. S. Cook, D. W. Cramer, C. Cybulski, A. Dansonka-Mieszkowska, E. Despierre, J. A. Doherty, T. Dörk, A. du Bois, M. Dürst, D. Eccles, R. Edwards, A. B. Ekici, P. A. Fasching, D. Fenstermacher, J. Flanagan, Y.-T. Gao, M. Garcia-Closas, A. Gentry-Maharaj, G. Giles, A. Gjyshi, M. Gore, J. Gronwald, Q. Guo, M. K. Halle, P. Harter, A. Hein, F. Heitz, P. Hillemanns, M. Hoatlin, E. Høgdall, C. K. Høgdall, S. Hosono, A. Jakubowska, A. Jensen, K. R. Kalli, B. Y. Karlan, L. E. Kelemen, L. A. Kiemeney, S. K. Kjaer, G. E. Konecny, C. Krakstad, J. Kupryjanczyk, D. Lambrechts, S. Lambrechts, N. D. Le, N. Lee, J. Lee, A. Leminen, B. K. Lim, J. Lissowska, J. Lubiński, L. Lundvall, G. Lurie, Leon F A G Massuger, K. Matsuo, V. M. Guire, J. R. Mc Laughlin, U. Menon, F. Modugno, K. B. Moysich, T. Nakanishi, S. A. Narod, R. B. Ness, H. Nevanlinna, S. Nickels, H. Noushmehr, K. Odunsi, S. Olson, I. Orlow, J. Paul, T. Pejovic, L. M. Pelttari, J. Permuth-Wey, M. C. Pike, E. M. Poole, X. Qu, H. A. Risch, L. Rodriguez-Rodriguez, M. A. Rossing, A. Rudolph, I. Runnebaum, I. K. Rzepecka, H. B. Salvesen, I. Schwaab, G. Severi, H. Shen, V. Shridhar, X.-O. Shu, W. Sieh, M. C. Southey, P. Spellman, K. Tajima, S.-H. Teo, K. L. Terry, P. J. Thompson, A. Timorek, S. S. Tworoger, Anne M van Altena, D. van den Berg, I. Vergote, R. A. Vierkant, A. F. Vitonis, S. Wang-Gohrke, N. Wentzensen, A. S. Whittemore, E. Wik, B. Winterhoff, Y. L. Woo, A. H. Wu, H. P. Yang, W. Zheng, A. Ziogas, F. Zulkifli, M. T. Goodman, P. Hall, D. F. Easton, C. L. Pearce, A. Berchuck, G. Chenevix-Trench, E. Iversen, A. N. A. Monteiro, S. A. Gayther, J. M. Schildkraut, T. A. Sellers, GWAS meta-analysis and replication identifies three new susceptibility loci for ovarian cancer. *Nat. Genet.* **45**, 362–370 (2013).

10. J. B. A. Sadler, D. M. Wenzel, L. K. Strohacker, M. Guindo-Martínez, S. L. Alam, J. M. Mercader, D. Torrents, K. S. Ullman, W. I. Sundquist, J. Martin-Serrano, A cancer-associated polymorphism in ESCRT-III disrupts the abscission checkpoint and promotes genome instability. *Proc. Natl. Acad. Sci. U.S.A.* **115**, E8900–E8908 (2018).

11. L. K. Strohacker, D. R. Mackay, M. A. Whitney, G. C. Couldwell, W. I. Sundquist, K. S. Ullman, Identification of abscission checkpoint bodies as structures that regulate ESCRT factors to control abscission timing. *eLife* **10**, e63743 (2021).

12. D. L. Spector, A. I. Lamond, Nuclear speckles. *Cold Spring Harb. Perspect. Biol.* **3**, a000646 (2011).

13. Y. Shin, C. P. Brangwynne, Liquid phase condensation in cell physiology and disease. *Science* **357**, eaaf4382 (2017).

14. S. Boeynaems, S. Alberti, N. L. Fawzi, T. Mittag, M. Polymenidou, F. Rousseau, J. Schymkowitz, J. Shorter, B. Wolozin, L. van den Bosch, P. Tompa, M. Fuxreiter, Protein phase separation: A new phase in cell biology. *Trends Cell Biol.* **28**, 420–435 (2018).

15. A. C. Murthy, N. L. Fawzi, The (un)structural biology of biomolecular liquid-liquid phase separation using NMR spectroscopy. *J. Biol. Chem.* **295**, 2375–2384 (2020).

16. Q. Su, S. Mehta, J. Zhang, Liquid-liquid phase separation: Orchestrating cell signaling through time and space. *Mol. Cell* **81**, 4137–4146 (2021).

17. A. K. Rai, J.-X. Chen, M. Selbach, L. Pelkmans, Kinase-controlled phase transition of membraneless organelles in mitosis. *Nature* **559**, 211–216 (2018).

18. A. R. Strom, C. P. Brangwynne, The liquid nucleome–phase transitions in the nucleus at a glance. *J. Cell Sci.* **132**, jcs235093 (2019).

19. M. H. H. Schmidt, I. Dikic, O. Bögler, Src phosphorylation of Alix/AIP1 modulates its interaction with binding partners and antagonizes its activities. *J. Biol. Chem.* **280**, 3414–3425 (2005).

20. K. Kasahara, Y. Nakayama, Y. Nakazato, K. Ikeda, T. Kuga, N. Yamaguchi, Src signaling regulates completion of abscission in cytokinesis through ERK/MAPK activation at the midbody. *J. Biol. Chem.* **282**, 5327–5339 (2007).

21. M. Stuible, J. V. Abella, M. Feldhammer, M. Nossov, V. Sangwan, B. Blagoev, M. Park, M. L. Tremblay, PTP1B targets the endosomal sorting machinery. *J. Biol. Chem.* **285**, 23899–23907 (2010).

22. R. D. Elias, W. Ma, R. Ghirlando, C. D. Schwieters, V. S. Reddy, L. Deshmukh, Proline-rich domain of human ALIX contains multiple TSG101-UEV interaction sites and forms phosphorylation-mediated reversible amyloids. *Proc. Natl. Acad. Sci. U.S.A.* **117**, 24274–24284 (2020).

23. R. D. Elias, B. Ramaraju, L. Deshmukh, Mechanistic roles of tyrosine phosphorylation in reversible amyloids, autoinhibition, and endosomal membrane association of ALIX. *J. Biol. Chem.* **297**, 101328 (2021).

24. C. J. Woolstenhulme, N. R. Guydosh, R. Green, A. R. Buskirk, High-precision analysis of translational pausing by ribosome profiling in bacteria lacking EFP. *Cell Rep.* **11**, 13–21 (2015).

25. H. H. Lee, N. Elia, R. Ghirlando, J. Lippincott-Schwartz, J. H. Hurley, Midbody targeting of the ESCRT machinery by a noncanonical coiled coil in CEP55. *Science* **322**, 576–580 (2008).

26. T. G. M. Schmidt, A. Skerra, The Strep-tag system for one-step purification and high-affinity detection or capturing of proteins. *Nat. Protoc.* **2**, 1528–1535 (2007).

27. Q. Zhai, M. B. Landesman, H.-Y. Chung, A. Dierkers, C. M. Jeffries, J. Trewhella, C. P. Hill, W. I. Sundquist, Activation of the retroviral budding factor ALIX. *J. Virol.* **85**, 9222–9226 (2011).

28. Y. Fezoui, D. M. Hartley, J. D. Harper, R. Khurana, D. M. Walsh, M. M. Condron, D. J. Selkoe, P. T. Lansbury, A. L. Fink, D. B. Teplow, An improved method of preparing the amyloid β-protein for fibrillogenesis and neurotoxicity experiments. *Amyloid* **7**, 166–178 (2000).

29. L. Hou, H. Shao, Y. Zhang, H. Li, N. K. Menon, E. B. Neuhaus, J. M. Brewer, I.-J. L. Byeon, D. G. Ray, M. P. Vitek, T. Iwashita, R. A. Makula, A. B. Przybyla, M. G. Zagorski, Solution NMR studies of the Aβ(1−40) and Aβ(1−42) peptides establish that the Met35 oxidation state affects the mechanism of amyloid formation. *J. Am. Chem. Soc.* **126**, 1992–2005 (2004).

30. N. C. Shaner, G. G. Lambert, A. Chammas, Y. Ni, P. J. Cranfill, M. A. Baird, B. R. Sell, J. R. Allen, R. N. Day, M. Israelsson, M. W. Davidson, J. Wang, A bright monomeric green fluorescent protein derived from Branchiostoma lanceolatum. *Nat. Methods* **10**, 407–409 (2013).

31. E. Gasteiger, C. Hoogland, A. Gattiker, S. Duvaud, M. R. Wilkins, R. D. Appel, A. Bairoch, in *The Proteomics Protocols Handbook*, J. M. Walker, Ed. (Humana Press, 2005), pp. 571–607.

32. M. Bajorek, H. L. Schubert, J. McCullough, C. Langelier, D. M. Eckert, W.-M. B. Stubblefield, N. T. Uter, D. G. Myszka, C. P. Hill, W. I. Sundquist, Structural basis for ESCRT-III protein autoinhibition. *Nat. Struct. Mol. Biol.* **16**, 754–762 (2009).

33. S. Shim, L. A. Kimpler, P. I. Hanson, Structure/function analysis of four core ESCRT-III proteins reveals common regulatory role for extreme C-terminal domain. *Traffic* **8**, 1068–1079 (2007).

34. J. Jumper, R. Evans, A. Pritzel, T. Green, M. Figurnov, O. Ronneberger, K. Tunyasuvunakool, R. Bates, A. Žídek, A. Potapenko, A. Bridgland, C. Meyer, S. A. A. Kohl, A. J. Ballard, A. Cowie, B. Romera-Paredes, S. Nikolov, R. Jain, J. Adler, T. Back, S. Petersen, D. Reiman, E. Clancy, M. Zielinski, M. Steinegger, M. Pacholska, T. Berghammer, S. Bodenstein, D. Silver, O. Vinyals, A. W. Senior, K. Kavukcuoglu, P. Kohli, D. Hassabis, Highly accurate protein structure prediction with AlphaFold. *Nature* **596**, 583–589 (2021).

35. M. Kjaergaard, S. Brander, F. M. Poulsen, Random coil chemical shift for intrinsically disordered proteins: Effects of temperature and pH. *J. Biomol. NMR* **49**, 139–149 (2011).

36. M. Kjaergaard, F. M. Poulsen, Sequence correction of random coil chemical shifts: Correlation between neighbor correction factors and changes in the Ramachandran distribution. *J. Biomol. NMR* **50**, 157–165 (2011).

37. Y. Shen, A. Bax, Protein backbone and sidechain torsion angles predicted from NMR chemical shifts using artificial neural networks. *J. Biomol. NMR* **56**, 227–241 (2013).

38. Y. Shen, J. Roche, A. Grishaev, A. Bax, Prediction of nearest neighbor effects on backbone torsion angles and NMR scalar coupling constants in disordered proteins. *Protein Sci.* **27**, 146–158 (2018).

39. J. R. Gillespie, D. Shortle, Characterization of long-range structure in the denatured state of staphylococcal nuclease. I. Paramagnetic relaxation enhancement by nitroxide spin labels. *J. Mol. Biol.* **268**, 158–169 (1997).

40. M. M. Dedmon, K. Lindorff-Larsen, J. Christodoulou, M. Vendruscolo, C. M. Dobson, Mapping long-range interactions in α-Synuclein using spin-label NMR and ensemble molecular dynamics simulations. *J. Am. Chem. Soc.* **127**, 476–477 (2005).

41. C. W. Bertoncini, Y.-S. Jung, C. O. Fernandez, W. Hoyer, C. Griesinger, T. M. Jovin, M. Zweckstetter, Release of long-range tertiary interactions potentiates aggregation of natively unstructured α-synuclein. *Proc. Natl. Acad. Sci. U.S.A.* **102**, 1430–1435 (2005).

42. S. Tyukhtenko, L. Deshmukh, V. Kumar, J. Lary, J. Cole, V. Lemmon, O. Vinogradova, Characterization of the neuron-specific L1-CAM cytoplasmic tail: Naturally disordered in solution it exercises different binding modes for different adaptor proteins. *Biochemistry* **47**, 4160–4168 (2008).

43. P. E. Wright, H. J. Dyson, Intrinsically disordered proteins in cellular signalling and regulation. *Nat. Rev. Mol. Cell Biol.* **16**, 18–29 (2015).

44. N. L. Fawzi, J. Ying, R. Ghirlando, D. A. Torchia, G. M. Clore, Atomic-resolution dynamics on the surface of amyloid-β protofibrils probed by solution NMR. *Nature* **480**, 268–272 (2011).

45. B. Ramaraju, S. L. Nelson, W. Zheng, R. Ghirlando, L. Deshmukh, Quantitative NMR study of insulin-degrading enzyme using amyloid-β and HIV-1 p6 elucidates its chaperone activity. *Biochemistry* **60**, 2519–2523 (2021).

46. M. Fuxreiter, I. Simon, P. Friedrich, P. Tompa, Preformed structural elements feature in partner recognition by intrinsically unstructured proteins. *J. Mol. Biol.* **338**, 1015–1026 (2004).

47. M. Arai, K. Sugase, H. J. Dyson, P. E. Wright, Conformational propensities of intrinsically disordered proteins influence the mechanism of binding and folding. *Proc. Natl. Acad. Sci. U.S.A.* **112**, 9614–9619 (2015).

48. L. Mollica, L. M. Bessa, X. Hanoulle, M. R. Jensen, M. Blackledge, R. Schneider, Binding mechanisms of intrinsically disordered proteins: Theory, simulation, and experiment. *Front. Mol. Biosci.* **3**, 52 (2016).

49. X. Zhang, M. Vigers, J. McCarty, J. N. Rauch, G. H. Fredrickson, M. Z. Wilson, J.-E. Shea, S. Han, K. S. Kosik, The proline-rich domain promotes Tau liquid-liquid phase separation in cells. *J. Cell Biol.* **219**, e202006054 (2020).

50. H. Jiang, S. Wang, Y. Huang, X. He, H. Cui, X. Zhu, Y. Zheng, Phase transition of spindle-associated protein regulate spindle apparatus assembly. *Cell* **163**, 108–122 (2015).

51. Y. Lin, S. L. Currie, M. K. Rosen, Intrinsically disordered sequences enable modulation of protein phase separation through distributed tyrosine motifs. *J. Biol. Chem.* **292**, 19110–19120 (2017).

52. C. W. Pak, M. Kosno, A. S. Holehouse, S. B. Padrick, A. Mittal, R. Ali, A. A. Yunus, D. R. Liu, R. V. Pappu, M. K. Rosen, Sequence determinants of intracellular phase separation by complex coacervation of a disordered protein. *Mol. Cell* **63**, 72–85 (2016).

53. T. H. Kim, B. J. Payliss, M. L. Nosella, I. T. W. Lee, Y. Toyama, J. D. Forman-Kay, L. E. Kay, Interaction hot spots for phase separation revealed by NMR studies of a CAPRIN1 condensed phase. *Proc. Natl. Acad. Sci. U.S.A.* **118**, e2104897118 (2021).

54. T. H. Kim, B. Tsang, R. M. Vernon, N. Sonenberg, L. E. Kay, J. D. Forman-Kay, Phospho-dependent phase separation of FMRP and CAPRIN1 recapitulates regulation of translation and deadenylation. *Science* **365**, 825–829 (2019).

55. V. H. Ryan, T. M. Perdikari, M. T. Naik, C. F. Saueressig, J. Lins, G. L. Dignon, J. Mittal, A. C. Hart, N. L. Fawzi, Tyrosine phosphorylation regulates hnRNPA2 granule protein partitioning and reduces neurodegeneration. *EMBO J.* **40**, e105001 (2021).

56. M. S. Choy, Y. Li, L. E. S. F. Machado, M. B. A. Kunze, C. R. Connors, X. Wei, K. Lindorff-Larsen, R. Page, W. Peti, Conformational rigidity and protein dynamics at distinct timescales regulate PTP1B activity and allostery. *Mol. Cell* **65**, 644–658.e5 (2017).

57. J. R. Huth, C. A. Bewley, B. M. Jackson, A. G. Hinnebusch, G. M. Clore, A. M. Gronenborn, Design of an expression system for detecting folded protein domains and mapping macromolecular interactions by NMR. *Protein Sci.* **6**, 2359–2364 (1997).

58. P. K. Smith, R. I. Krohn, G. T. Hermanson, A. K. Mallia, F. H. Gartner, M. D. Provenzano, E. K. Fujimoto, N. M. Goeke, B. J. Olson, D. C. Klenk, Measurement of protein using bicinchoninic acid. *Anal. Biochem.* **150**, 76–85 (1985).

59. H. Zhao, C. A. Brautigam, R. Ghirlando, P. Schuck, Overview of current methods in sedimentation velocity and sedimentation equilibrium analytical ultracentrifugation. *Curr. Protoc. Protein Sci.* **20**, Unit20.12 (2013).

60. L. Deshmukh, C. D. Schwieters, A. Grishaev, R. Ghirlando, J. L. Baber, G. M. Clore, Structure and dynamics of full-length HIV-1 capsid protein in solution. *J. Am. Chem. Soc.* **135**, 16133–16147 (2013).

61. L. Deshmukh, R. Ghirlando, G. M. Clore, Investigation of the structure and dynamics of the capsid-spacer peptide 1-nucleocapsid fragment of the HIV-1 Gag polyprotein by solution NMR spectroscopy. *Angew. Chem. Int. Ed. Engl.* **53**, 1025–1028 (2014).

62. L. Deshmukh, J. M. Louis, R. Ghirlando, G. M. Clore, Transient HIV-1 Gag-protease interactions revealed by paramagnetic NMR suggest origins of compensatory drug resistance mutations. *Proc. Natl. Acad. Sci. U.S.A.* **113**, 12456–12461 (2016).

63. J. Schindelin, I. Arganda-Carreras, E. Frise, V. Kaynig, M. Longair, T. Pietzsch, S. Preibisch, C. Rueden, S. Saalfeld, B. Schmid, J.-Y. Tinevez, D. J. White, V. Hartenstein, K. Eliceiri, P.

Tomancak, A. Cardona, Fiji: An open-source platform for biological-image analysis. *Nat. Methods* **9**, 676–682 (2012).

64. F. Delaglio, S. Grzesiek, G. W. Vuister, G. Zhu, J. Pfeifer, A. Bax, NMRPipe: A multidimensional spectral processing system based on UNIX pipes. *J. Biomol. NMR* **6**, 277–293 (1995).

65. W. F. Vranken, W. Boucher, T. J. Stevens, R. H. Fogh, A. Pajon, M. Llinas, E. L. Ulrich, J. L. Markley, J. Ionides, E. D. Laue, The CCPN data model for NMR spectroscopy: Development of a software pipeline. *Proteins* **59**, 687–696 (2005).

66. G. M. Clore, A. M. Gronenborn, Determining the structures of large proteins and protein complexes by NMR. *Trends Biotechnol.* **16**, 22–34 (1998).

67. J. Roche, J. Ying, A. Bax, Accurate measurement of $^{3}J_{HNH\alpha}$ couplings in small or disordered proteins from WATERGATE-optimized TROSY spectra. *J. Biomol. NMR* **64**, 1–7 (2016).

68. N.-A. Lakomek, J. Ying, A. Bax, Measurement of ^{15}N relaxation rates in perdeuterated proteins by TROSY-based methods. *J. Biomol. NMR* **53**, 209–221 (2012).

69. M. Bastidas, E. B. Gibbs, D. Sahu, S. A. Showalter, A primer for carbon-detected NMR applications to intrinsically disordered proteins in solution. *Concepts Magn. Reson. A: Bridg. Educ. Res.* **44**, 54–66 (2015).

70. L. Deshmukh, R. Ghirlando, G. M. Clore, Conformation and dynamics of the Gag polyprotein of the human immunodeficiency virus 1 studied by NMR spectroscopy. *Proc. Natl. Acad. Sci. U.S.A.* **112**, 3374–3379 (2015).

71. Q. Zhai, M. B. Landesman, H. Robinson, W. I. Sundquist, C. P. Hill, Identification and structural characterization of the ALIX-binding late domains of simian immunodeficiency virus SIVmac239 and SIVagmTan-1. *J. Virol.* **85**, 632–637 (2011).

72. D. M. Wenzel, D. R. Mackay, J. J. Skalicky, E. L. Paine, M. S. Miller, K. S. Ullman, W. I. Sundquist, Comprehensive analysis of the human ESCRT-III-MIT domain interactome reveals new cofactors for cytokinetic abscission. *eLife* **11**, e77779 (2022).

73. E. Morita, V. Sandrin, H.-Y. Chung, S. G. Morham, S. P. Gygi, C. K. Rodesch, W. I. Sundquist, Human ESCRT and ALIX proteins interact with proteins of the midbody and function in cytokinesis. *EMBO J.* **26**, 4215–4227 (2007).

74. T. G. Flower, Y. Takahashi, A. Hudait, K. Rose, N. Tjahjono, A. J. Pak, A. L. Yokom, X. Liang, H.-G. Wang, F. Bouamr, G. A. Voth, J. H. Hurley, A helical assembly of human ESCRT-I scaffolds reverse-topology membrane scission. *Nat. Struct. Mol. Biol.* **27**, 570–580 (2020).

75. S. L. Nelson, Y. Li, Y. Chen, L. Deshmukh, Avidity-based method for the efficient generation of monoubiquitinated recombinant proteins. *J. Am. Chem. Soc.* **145**, 7748–7752 (2023).

76. P. Sette, J. A. Jadwin, V. Dussupt, N. F. Bello, F. Bouamr, The ESCRT-associated protein Alix recruits the ubiquitin ligase Nedd4-1 to facilitate HIV-1 release through the $LYPX_nL$ L domain motif. *J. Virol.* **84**, 8181–8192 (2010).

77. B. Korbei, Ubiquitination of the ubiquitin-binding machinery: How early ESCRT components are controlled. *Essays Biochem.* **66**, 169–177 (2022).

78. A. Tedeschi, J. Almagro, M. J. Renshaw, H. A. Messal, A. Behrens, M. Petronczki, Cep55 promotes cytokinesis of neural progenitors but is dispensable for most mammalian cell divisions. *Nat. Commun.* **11**, 1746 (2020).

79. A. Lie-Jensen, K. Ivanauskiene, L. Malerød, A. Jain, K. W. Tan, J. K. Laerdahl, K. Liestøl, H. Stenmark, K. Haglund, Centralspindlin recruits ALIX to the midbody during cytokinetic abscission in *Drosophila* via a mechanism analogous to virus budding. *Curr. Biol.* **29**, 3538–3548.e7 (2019).

80. D. Sharma, K. Rajarathnam, ^{13}C NMR chemical shifts can predict disulfide bond formation. *J. Biomol. NMR* **18**, 165–171 (2000).

81. M. R. Jensen, M. Zweckstetter, J.-R. Huang, M. Blackledge, Exploring free-energy landscapes of intrinsically disordered proteins at atomic resolution using NMR spectroscopy. *Chem. Rev.* **114**, 6632–6660 (2014).

82. T. Kakeshpour, V. Ramanujam, C. A. Barnes, Y. Shen, J. Ying, A. Bax, A lowly populated, transient β-sheet structure in monomeric A$\beta^{1\text{-}42}$ identified by multinuclear NMR of chemical denaturation. *Biophys. Chem.* **270**, 106531 (2021).
